# Loss of grand histone H3 lysine 27 trimethylation domains mediated transcriptional activation in esophageal squamous cell carcinoma

**DOI:** 10.1038/s41525-021-00232-6

**Published:** 2021-08-11

**Authors:** Jian Yuan, Qi Jiang, Tongyang Gong, Dandan Fan, Ji Zhang, Fukun Chen, Xiaolin Zhu, Xinyu Wang, Yunbo Qiao, Hongyan Chen, Zhihua Liu, Jianzhong Su

**Affiliations:** 1 School of Biomedical Engineering, School of Ophthalmology & Optometry and Eye Hospital, Wenzhou Medical University, Wenzhou, China; 2 Institute of Biomedical Big Data, Wenzhou Medical University, Wenzhou, China; 3 State Key Laboratory of Molecular Oncology, National Cancer Center/National Clinical Research Center for Cancer/Cancer Hospital, Chinese Academy of Medical Sciences and Peking Union Medical College, Beijing, China; 4 Oujiang Laboratory, Wenzhou, China; 5 Precise Genome Engineering Center, School of Life Sciences, Guangzhou University, Guangzhou, China

**Keywords:** Oesophageal cancer, Cancer genomics

## Abstract

Trimethylation of histone H3 lysine 27 trimethylation (H3K27me3) may be recruited by repressive Polycomb complexes to mediate gene silencing, which is critical for maintaining embryonic stem cell pluripotency and differentiation. However, the roles of aberrant H3K27me3 patterns in tumorigenesis are not fully understood. Here, we discovered that grand silencer domains (breadth > 50 kb) for H3K27me3 were significantly associated with epithelial cell differentiation and exhibited high gene essentiality and conservation in human esophageal epithelial cells. These grand H3K27me3 domains exhibited high modification signals involved in gene silencing, and preferentially occupied the entirety of topologically associating domains and interact with each other. We found that widespread loss of the grand H3K27me3 domains in of esophageal squamous cell carcinomas (ESCCs) were enriched in genes involved in epithelium and endothelium differentiation, which were significantly associated with overexpression with increase of active modifications of H3K4me3, H3K4me1, and H3K27ac marks, as well as DNA hypermethylation in the gene bodies. A total of 208 activated genes with loss of grand H3K27me3 domains in ESCC were identified, where the higher expression and mutation of T-box transcription factor 20 (TBX20) were associated with worse patients’ outcomes. Our results showed that knockdown of TBX20 may have led to a striking defect in esophageal cancer cell growth and carcinogenesis-related pathway, including cell cycle and homologous recombination. Together, our results reveal that loss of grand H3K27me3 domains represent a catalog of remarkable activating regulators involved in carcinogenesis.

## Introduction

Histone methylation primarily on lysine and arginine residues is governed by multiple positive and negative regulators, to either activate or repress transcription, which are essential for cell-fate determination and embryonic stem cell pluripotency and differentiation^1^. Abnormal expression patterns or genomic alterations in histone methylation can lead to the induction and maintenance of various cancers^2,3^. One of the best examples of deregulated histone methylation resulting in changes of gene expression and genome integrity in cancer is the extensive loss and gain of histone H3 lysine 27 trimethylation (H3K27me3). The recurrent gain-of-function or loss-of-function mutations in the gene encoding enhancer of zeste homolog 2 (EZH2) is the one of the catalytic subunits of Polycomb repressive complex 2 (PRC2) and catalyzes the methylation of H3K27me3^4^. Gain-of-function mutations leading to the overexpression of PRC2 components or enhancement of the catalytic activity of EZH2 lead to an aberrant increase in global levels of H3K27me3^5,6^. However, loss-of-function mutations leading to a decrease in PRC2 activity and affecting the H3K27 substrate of PRC2 have been found in several types of cancer, resulting in a decrease in the global level of H3K27me3^7,8^. Together, aberrant H3K27me3 in different tumor contexts is thought to reflect the crucial roles of the specific cellular transcriptional programme and chromatin environment^9^.

Specific transcription outputs could be contained in the spread of epigenetic modifications over a genomic locus. Active chromatin marks are usually restricted to specific genomic loci but have also been observed in broader deposits^10^. Super-enhancers, have been used to describe large clusters of H3K27ac peaks, were specifically drive the expression of cell identity genes and associated with key oncogenes in many cancer types^11–13^. In addition, the broad H3K4me3 domains have been found to associate not only with increased transcriptional precision of cell-type-specific genes related to cell identity but also with the increased transcriptional elongation and enhancer activity of tumor-suppressor genes^14,15^. More recently, several studies have proposed methods to identity broad H3K27me3 domain in a genome-wide manner and found these broad genic repression domains serve as a strong repressive machinery via chromatin interactions and signify enhanced silencing of oncogenes^16,17^. Taken together, these findings indicated that modification breadth is essential for the regulation of the transcriptional output across multiple cell or tumor types.

Esophageal cancer is the sixth leading cause of cancer-related mortality worldwide. In China, esophageal squamous cell carcinomas (ESCCs) are the predominant histological subtype of esophageal cancer and fourth leading cause of cancer death^18,19^. Aberrant keratinocyte differentiation is considered to be a key mechanism in the initiation of ESCC, which is driven by genomic changes of lineage-specific transcription factors (TFs) such as mutations in Notch1/2/3 and ZNF750 and copy number amplifications of SOX2 and TP63^20^. However, how H3K27me3 and other chromatin modifications influence cell differentiation and development of ESCC remains unclear.

In this study, we generated genome-wide maps of H3K27me3, H3K4me3, H3K27ac H3K4me1, and DNA methylation, as well as gene expression profiles in both normal (NE2) and cancer cell lines (KYSE450) of ESCC to investigate the role of the epigenetic alteration in oncogenesis. We found that these extremely grand H3K27me3 domains are of high density and represses transcriptional activity more strongly than typical domains. Remarkably, genes with loss of grand H3K27me3 domains were preferentially enriched in epithelium/endothelial cell differentiation and blood vessel development involved in carcinogenesis. Using the grand H3K27me3 domains, we discovered the novel carcinogenic genes of T-box TF 20 (TBX20) in ESCC and experimentally validated our findings in vitro and in vivo.

## Results

### Top 5% broadest H3K27me3 domains preferentially mark cell-fate determination in esophageal epithelial cell line

**Fig. 1:**
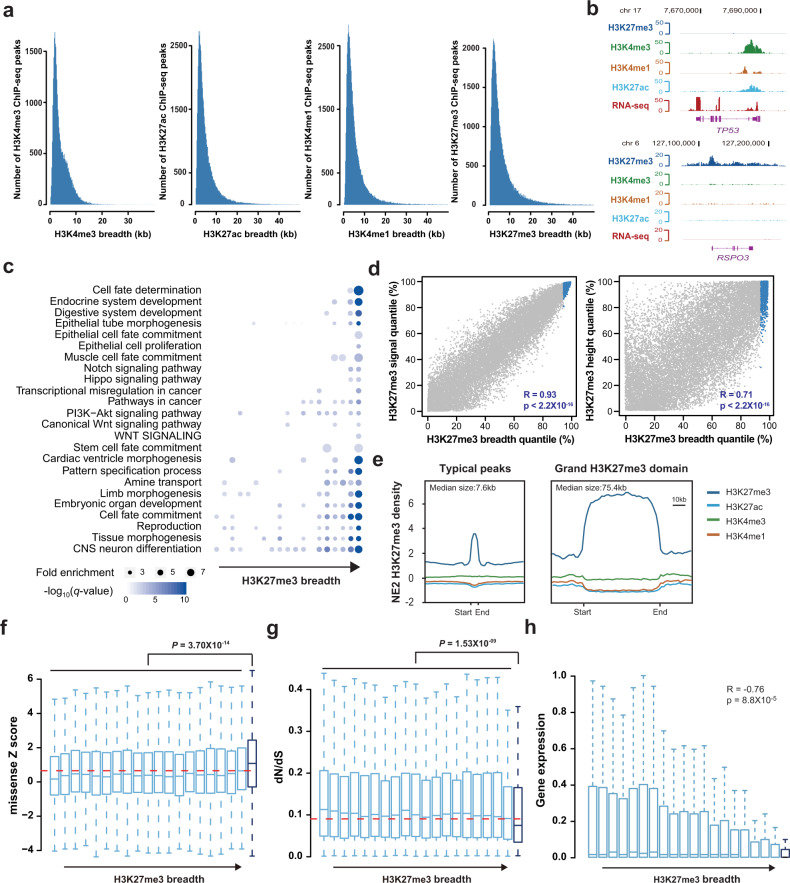
Grand H3K27me3 silencer domains marked subsets of genes in esophageal epithelial cell line. **a** Breadth distribution of H3K27me3, H3K4me3, H3K4me1, and H3K27ac ChIP-seq peaks in human esophageal epithelial cell line (NE2). Inserts example histone modification regions with different width in NE2; black bar represent ChIP-seq peaks called by SICER. **b** H3K27me3, H3K4me3, H3K4me1, and H3K27ac density at the tumor-suppressor *TP53* and oncogene RSPO3 in NE2 cells. **c** Functional enrichment for genes along the H3K27me3 breadth continuum. The color and size of each point represented the −log_10_ (*P* value) values and enrichment scores. **d** The index of H3K27me3 peak signal or peak height (*y*-axis) plotted against peak breadth (*x*-axis). Blue dots indicate the top 5% broadest H3K27me3 domains which we defined “grand H3K27me3 silencer domains.” **e** Metagenes of H3K27me3, H3K4me3, H3K4me1, and H3K27ac ChIP-seq density (reads per kilobase per million mapped reads). Metagenes are centered on the H3K27me3 region (7.6 kb for typical peaks and 75.4 kb for grand H3K27me3 domains), with 3 kb surrounding each peak region. Boxplots representing the missense *Z* scores (**f**), the ratio of substitution rates, denoted dN/dS, between human and mouse (**g**) and gene expression level, and TPM (**h**) associated with various H3K27me3 domain breadths in NE2.

To investigate the importance of epigenomic modification in stabilizing normal cell identity and in carcinogenesis of ESCC, we first generated ChIP-seq datasets for H3K27me3, H3K27ac, H3K4me1, and H3K4me3 and RNA-seq in human esophageal epithelial cell line (NE2). Although the vast majority of H3K27me3 domains was present in 4-5 kb regions, the H3K27me3 domains had a skewness distribution, in which broadest H3K27me3 domains can spanning up to 300 kb in NE2 cells (Fig. 1a). Consistent with the notions that exceptionally broad H3K4me3 signature marks tumor-suppressor genes^15^ and that broad H3K27me3 marks oncogenes^16^, we observed that the ChIP-seq for H3K4me3 can cover up to 10 kb in the well-known tumor-suppressor gene, TP53^21^, and that broad H3K27me3 domains spanned up to 100 kb, which were present in the RSPO3 oncogenes^22^ (Fig. 1b).

To assess whether broad H3K27me3 and other histone modification domains mark specific genes in human esophagus system, we performed gene ontology and pathway enrichment analysis for genes with distinct breadth domains. In NE2 cells, top 5% broadest H3K27me3 domain showed a striking pattern of most enrichment, along with the H3K27me3 breadth continuum, for genes involved in cell-fate determination (false discovery rate (FDR) = 1.0 × 10^−11^), as well as genes involved in epithelial tube morphogenesis (FDR = 2.0 × 10^−10^) (Fig. 1c, Supplementary Table 1). And the pathways in cancer (hsa05200) were also enriched in the broadest H3K27me3 domains (FDR = 1.5 × 10^−04^). In contrast, the broadest domains of other histone marks of H3K4me3, H3K4me1, and H3K27ac were not specifically enriched for epithelium development (Supplementary Fig. 1). As expected, we found that the set of 5% broadest H3K27me3 domains was enriched in oncogenes in NE2 cells (Supplementary Fig. 2). Taking these data together, we conclude that top 5% broadest H3K27me3 peaks in NE2 cells involved in specific gene repression related to cell-fate determination, epithelium development, and carcinogenesis.

These observations motivated us to perform a systematic analysis of the function characterization between top 5% broadest H3K27me3 domains and the remaining typical domains. Notably, we found broad H3K27me3 domains were strongly associated with higher ChIP-seq signal (total reads) and intensities (Fig. 1d). Based on functional specificity and high correlation with signal density, we defined the top 5% broadest peaks which greater than 50 kb in width and totally encompassed 813 H3K27me3 domains as grand H3K27me3 domain (Fig. 1d). The grand H3K27me3 domains span DNA regions whose median length is tenfold wider than the typical domains, and have levels of signal density that are at least a twofold greater than those at the typical domains (Fig. 1e). Consistent with previous reports^23^, H3K27me3 was mostly around located at the transcription start sites (TSSs), whereas grand H3K27me3 domains were consistently found to cover whole genes extending both TSS and TES among species (Supplementary Fig. 3).

To further investigate whether the grand H3K27me3 domains have features that might distinguish them from other H3K27me3 domains, we performed gene essentiality and conservation analyses with genes marked by H3K27me3 width quantile subsets. We found genes with the top 5% grand H3K27me3 domain have significant greater missense *Z* score (Fig. 1f, *P* = 3.70 × 10^−14^, Wilcoxon rank sum test) and lower dN/dS (Fig. 1g, *P* = 1.53 × 10^−09^, Wilcoxon rank sum test) than other genes without grand H3K27me3 domains, which implied that genes marked by grand H3K27me3 domains are more constrained and intolerance to the class of variation. A significant negative correlation was found between H3K27me3 domain breadth and gene expression, which indicated grand H3K27me3 domains thoroughly repressed gene expression in NE2 cells (Fig. 1h, *R* = −0.76, *P* = 8.8 × 10^−5^, Spearman’s rank correlation). Together, we uncovered grand H3K27me3 silence domains (GSDs) that represent a distinct subclass of H3K27me3 domains, which are strongly and uniquely associated with gene constraint and gene silencing in NE2 cells.

### Grand H3K27me3 silence domains coincide with topologically associating domains

**Fig. 2:**
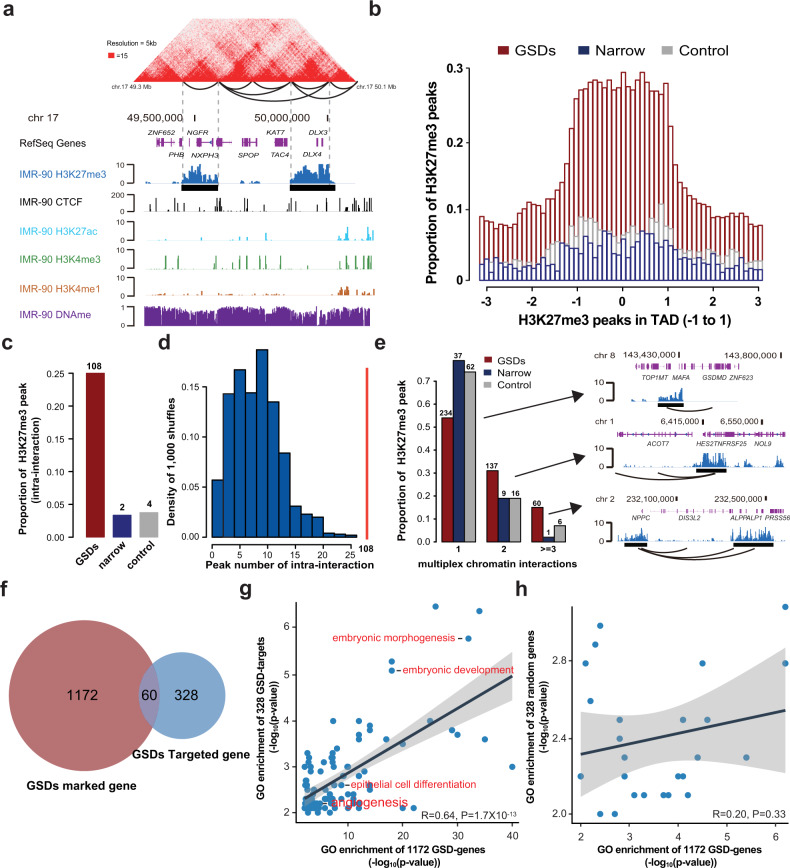
The distribution of grand H3K27me3 silencer domains is correlated with the 3D genome organization. **a** Example of Hi-C contact maps at 15-kb resolution of the genomic region Chr.17 49.3–50.1 Mb in normal lung fibroblast cell line IMR90. Interaction loops are contoured in black, and representative genes within the TADs are shown. H3K27me3, H3K4me3, H3K4me1, H3K27ac, and CTCF ChIP-seq and DNA methylation tracks on Chr.17 49.3–50.1 Mb for IMR90. **b** Position of peaks for grand H3K27me3 domains, narrow peaks, and control peaks across TADs. TADs are normalized to position 0, representing the TAD center, and positions −1 and 1 represent the TAD boundary. **c** Proportion of grand H3K27me3 domains, narrow peaks, or control peaks preferentially associate with each other via chromatin interactions. **d** The observed number of grand H3K27me3 domains that interact with other members (the red line) versus the numbers obtained by random permutation of grand H3K27me3 domain genomic location (the blue histogram, 1000 replications of permutations were performed). **e** Analysis of grand H3K27me3 domains mediated multiplexed interactions by categorizing all interactions into grand H3K27me3 domains to single interaction (1), double interaction (2), and multiplexed interaction (>3). Barplot shows the fractions of the grand H3K27me3 domains, narrow peak, and control peak among categories. Examples of browser view of grand H3K27me3 domains mediated interactions are shown. Contact profiles including the H3K27me3 density map and interactions (or loops) for the multiplexed capture are shown. **f** Venn diagram of gene comparison between GSDs marked and GSDs targeted. **g** Gene ontology and pathway enrichment significance (−log_10_ (*P* value) values) are correlated for GO terms shared between 1172 GSDs marked genes and 388 GSDs targeted genes. (Insert) Spearman correlation coefficients. **h** Gene ontology and pathway enrichment significance (−log_10_ (*P* value) values) are not correlated for GO terms shared between 1172 GSDs marked genes and 388 random selected genes. (Insert) Spearman correlation coefficients.

Although Polycomb-dependent chromatin looping contributes to gene silencing have also been described^24–26^, the relationship between higher-order chromatin structure and GSDs remains unclear. We used publicly available H3K27me3 ChIP-seq and Hi-C datasets from the IMR90 cells and found GSDs in IMR90 cells have a strongly positive correlation of functional enrichments with GSDs in NE2 cells (*R* = 0.84, *P* = 1.6 × 10^−201^ for enrichment score, Pearson’s correlation; Supplementary Fig. 4). By mapping H3K27me3 profiles with the Hi-C data in IMR90 cells, we observed that multiple anchors and topologically associated domains (TAD) often corresponded to regions of GSDs (Fig. 2a). In detail, we found that 643 out of 840 GSDs overlap with the TADs and 431 mark the anchors. To conduct a quantitative comparison of different breadth, we retrieved three subsets of H3K27me3 domain: top 5% broadest H3K27me3 domains (GSDs), top 5% narrowest H3K27me3 domains, and random 5% H3K27me3 domains as a control. Interestingly, we found 30% GSDs across the entirety of TAD in comparison with surrounding regions, which inferred these TADs might lead to the formation of repressed compartments (Fig. 2b). Similar patterns were also observed in fibroblast derived cell line (GM23248) and lymphoblastoid cell line (GM12878) (Supplementary Fig. 5). Compared GSDs with narrow and control domains, interactions between anchors inside GSDs were more likely to interact with each other (Fig. 2c). Alternatively, we calculated the expected number of interactions for shuffle GSDs under random simulations. The observed number of GSDs interacted with each other is significantly higher than the results obtained by randomly shuffling GSD assignments (*P* < 0.001, Fig. 2d). In addition, GSDs tend to interact with more anchors to form multiplex chromatin interactions (Fig. 2e), suggesting that they tend to cosegregate with multiple anchors simultaneously for a consequence of phase separation consistent with previous studies^27,28^.

To infer the target genes of GSDs and potential regulatory function, we defined putative target genes as genes that mapped within, or in distance (≤3 kb) to, a downstream interaction region. A total of 388 putative target genes were defined to the 430 anchor-related GSDs, and 60 genes were overlapped with 1172 GSD-marked genes (Fig. 2f). To understand the function of the putative GSD target genes, we analyzed the biological processes and pathways of the genes targeted by GSDs. Excluding 60 genes marked by GSDs from domain target genes, it was nominally enriched (*P* < 0.01) for 235 GO terms. Of these, 106 terms were enriched in both GSD-targeted genes and GSD-marked genes (Supplementary Table 2). Furthermore, we observed a significant positive correlation in enrichment *P* value for shared terms (*R* = 0.67, *P* = 8.9 × 10^−16^, Fig. 2g). GO term analysis of the GSD-targeted genes indicated enrichment of terms related to development and differentiation processes. In contrast, there was no correlation for function enrichment between GSD-marked genes and random selected genes (Fig. 2h). Together, our results demonstrate that the biological nature of GSDs tend to be chromatin looping and relevance to the coordinately regulation of functionally related genes.

### Widespread loss of GSDs induced gene activity in ESCC

**Fig. 3:**
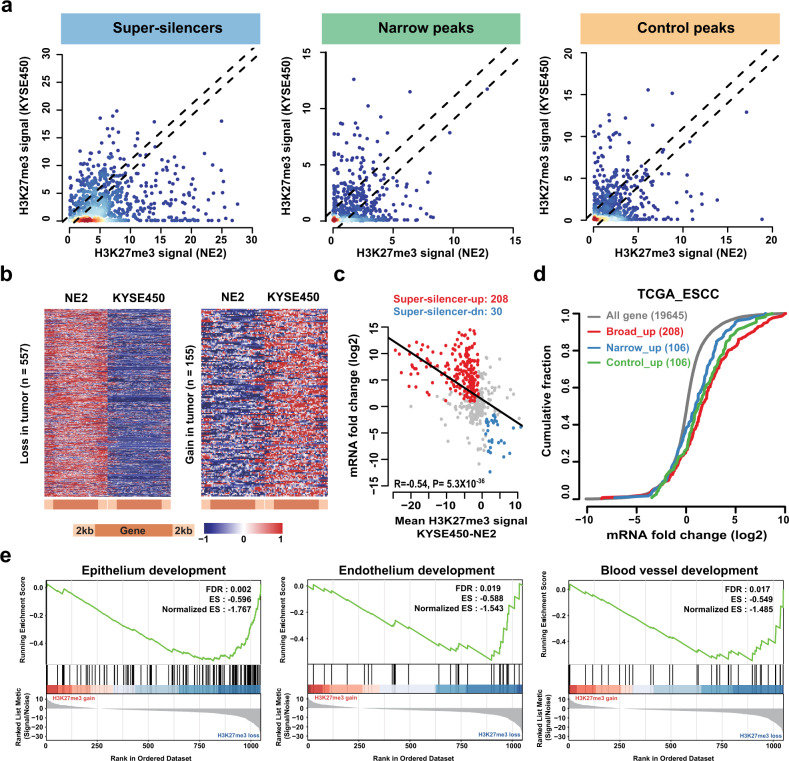
Widespread loss of grand H3K27me3 silencer domains in esophageal squamous cell carcinoma. **a** Scatterplot showing average H3K27me3 signal of indicated peak-related genes in KYSE450 (*y*-axis) and NE2 (*x*-axis). H3K27me3 gains and losses in KYSE450 cells, compared to NE2 cells (determined using > 1-fold), are outside the dotted lines, respectively. **b** In grand H3K27me3 domains marked gene, heatmaps showing H3K27me3 distribution in NE2 and KYSE450 cells. H3K27me3 on gene bodies (50 equal-sized bins) ± 2 kb (ten equal-sized bins) is represented on the brown scale. Each row represents the same gene in NE2 and KYSE450 cells. **c** H3K27me3 difference for GSDs marked gene (mean values; *x*-axis) plotted against expression of the corresponding genes (*y*-axis; log_2_ values) in KYSE450 cells versus NE2 cells (along axes): red, selected H3K27me3 losses and upregulated genes; blue, H3K27me3 gains and downregulated genes; and black diagonal line, linear regression. **d** Cumulative distribution of gene expression changes in TCGA ESCC. The *x*-axis is log_2_ (fold change) in TCGA tumor versus normal. The *y*-axis represents the cumulative fraction of genes: red, genes with grand H3K27me3 domains loss-induced upregulation; blue, narrow peak loss-induced gene upregulation 3; green, control peak loss-induced gene upregulation; and gray, entire mRNA. **e** GSEA of H3K27me3 losses in GSDs marked genes between NE2 and KYSE450 (normalized enrichment score (NES); false discovery rate-adjusted *P* value (FDR)).

To find out how loss or gain of GSDs affects gene expression levels in ESCC, we first compared their H3K27me3 levels and expression changes between cancer KYSE450 cells and normal cells (NE2) (Fig. 3a). We found that average H3K27me3 levels of GSD-marked genes but not narrow and control group were decreased in KYSE450 cells (Supplementary Fig. 6). Specifically, 557 of 844 (66.0%) GSDs associated genes and 155 (18.4%) GSDs associated genes showed H3K27me3 signal loss and gain in KYSE450 cells compared with NE2 cells, respectively (Fig. 3b). Next, we investigated the relationship between change in H3K27me3 signal and change in gene expression. As expected, H3K27me3 signal loss in KYSE450 cells was correlated with transcription upregulation, whereas gain of H3K27me3 signal was correlated with downregulated transcription (Fig. 3c). Only 30 of 155 (19.4%) genes that were gained H3K27me3 signal showed significantly downregulated than in KYSE450 cells. Conversely, 208 of the 557 genes (37.3%) that showed less H3K27me3 are highly expression in KYSE450 cells (Fig. 3c, Supplementary Table 3). Although narrow and random H3K27me3 peaks were also found to be correlated with differential gene expression, the strength of these correlation was smaller than GSDs (*R* = −0.54 for GSDs, −0.46 for narrow and random domains, Spearman’s correlation; Fig. 3c, Supplementary Fig. 7). To test the effect of H3K27me3 breadth loss on transcriptional consistency, we used H3K27me3 ChIP-seq dataset and expression RNA-seq obtained in another ESCC cell lines, KYSE510, and found the increased transcriptional expression associated with the loss of H3K27me3 signal (Supplementary Fig. 8).

We next examined whether the gene expression upregulated by H3K27me3 loss in ESCC cells also exist in human tumors. In the ESCC dataset from The Cancer Genome Atlas (TCGA), the 208 GSDs activated genes were also significantly upregulated compared to the background genes (*P* = 2.0 × 10^−36^, Wilcoxon rank sum test, Fig. 3d). Although narrow and random H3K27me3 peaks loss also results in gene activation, we observed stronger activity on GSDs associated genes than genes harboring narrow or control H3K27me3 peaks. The total 208 differentially expressed GSDs associated genes were mainly enriched in cell-fate commitment (FDR = 2.0 × 10^−08^) and blood vessel development (FDR = 4.0 × 10^−04^, Supplementary Fig. 9). Furthermore, gene set enrichment analysis (GSEA) of representative gene set in KYSE450 and KYSE510 cells on epithelium development, endothelium development, and blood vessel development is shown in Fig. 3e and Supplementary Fig. 10, which also indicated a significant correlation between upregulated epithelium and endothelium development programs and GSDs loss in ESCC.

### GSDs loss activated genes with acquired active promoter and hypermethylated gene body

**Fig. 4:**
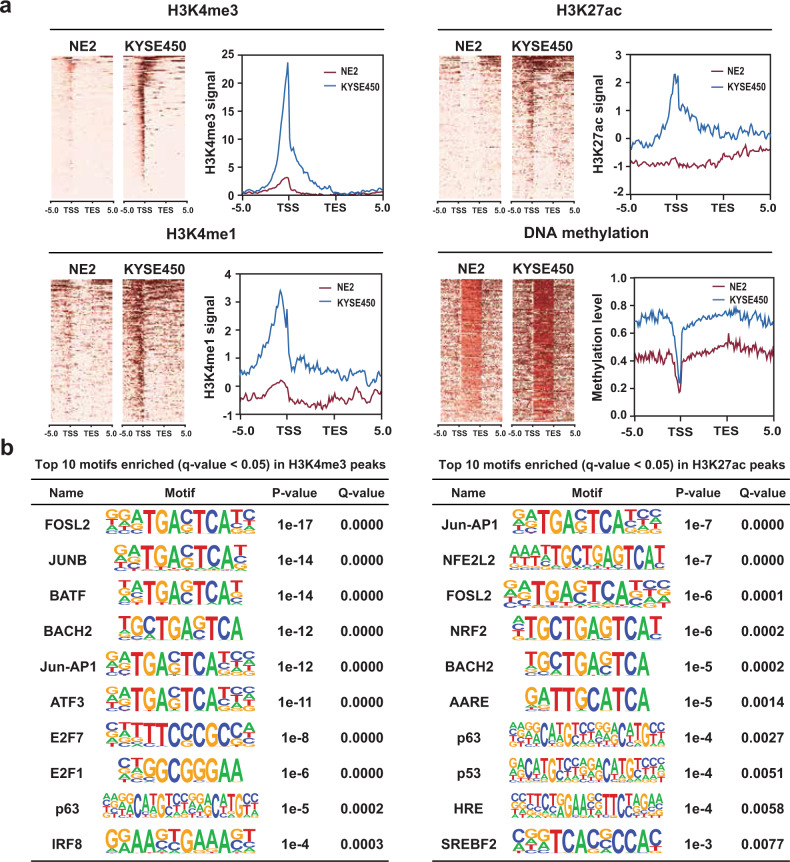
Grand H3K27me3 silencer domain breadth dynamics encodes epigenomic signature and cancer-associated TF binding. **a** H3K4me3, H3K4me1, H3K27ac, and DNA methylation density plots (left) and average density (right) across all modified regions in KYSE450 versus NE2 cell line. Columns represent the region from −5 kb with respect to the transcription start sites (TSSs) to +10 kb with respect to the transcription end site (TES). **b** Top ten TF-binding motifs enriched in H3K4me3, H3K4me1, and H3K27ac peaks of upregulated GSDs marked genes. Motif and statistical analysis were performed in HOMER software.

To determine the molecular basis of GSDs mediated gene activity, we performed integrated analyses of ChIP-seq and whole-genome bisulfite sequencing (WGBS) data in NE2 and KYSE450 cells. For 208 activated genes in cancer found associated with GSD loss, a significant increase was observed in H3K4me3, H3K4me1, and H3K27ac levels in KYSE450 cells (Fig. 4a). Notably, this was accompanied with an increase of DNA methylation level at gene body but not hypomethylation at promoters (Fig. 4a). In our previous study, we discovered a strong correlation between gene-body hypermethylation of DNA methylation canyons (large domains of low DNA methylation) and overexpression of homeobox genes^29^. It indicates that GSDs loss could induce de novo DNA methylation in gene body to maintain transcriptional stability.

To further explore genomic architecture of GSDs activated genes, we searched for TF-binding motifs enriched in the H3K4me3 and H3K27ac peaks of 208 upregulated genes using a motif analysis algorithm. In KYSE450 cells, TF-binding motifs were only enriched (Fisher’s exact test FDR < 0.05) in upregulated genes when using 50,000 background sequence regions as a control. The top scoring TF motifs were activator protein 1 (AP1) family, including FOSL2, JunB, BATF, ATF3, and Jun, in H3K4me3 and H3K27ac peaks (Fig. 4b), which suggests that the TF AP1 likely contribute to regulation of GSDs associated genes.

### RNA-seq analysis reveals novel gene signatures regulated by TBX20 in ESCC

**Fig. 5:**
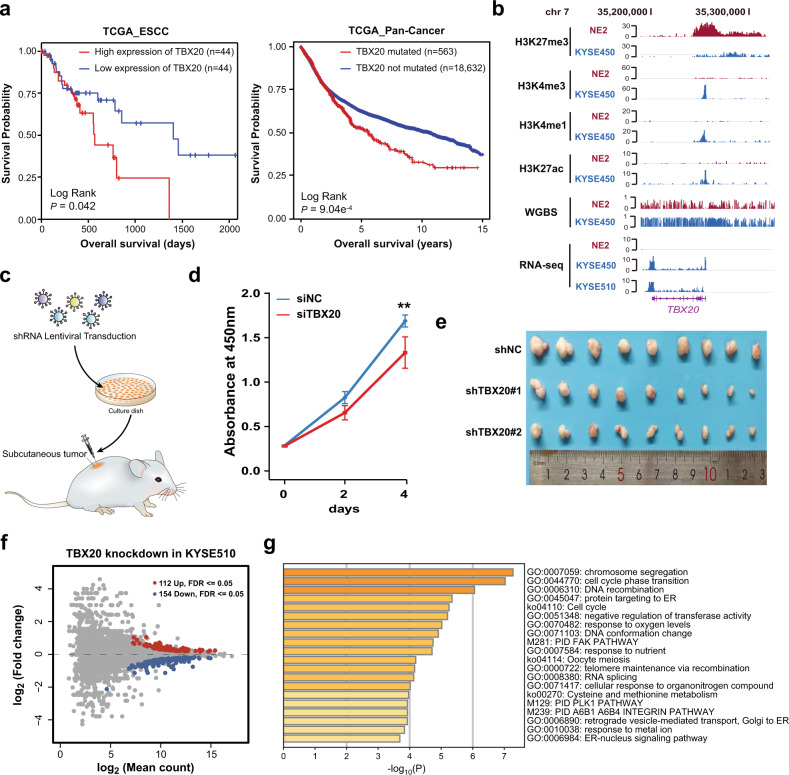
RNA-seq analysis reveals genes and pathways targeted by TBX20. **a** Kaplan–Meier estimates of overall survival of patients with low- or high-expression level of TBX20 in TCGA ESCC patients (left) and with mutated or not mutated TBX20 in TCGA Pan-Cancer (right). Statistical analysis was performed using the log-rank test and univariate Cox analysis. **b** Genomic snapshot of H3K27me3, H3K4me3, H3K4me1, H3K27ac, and RNA-seq in NE2, KYSE450, and KYSE510 at the TBX20 loci. **c** Experimental design to test the role of GSDs mediated oncogenesis candidates in cell proliferation and subcutaneous tumor growth. **d** Cell proliferation was measured using the CCK-8 assay. Error bars, s.d. calculated from five replicates. ***P < 0.001, **P < 0.01, **P < 0.05, compared to control siRNA in Wilcoxon rank sum test. **e** TBX20 silencing inhibited tumor growth as measured by tumor weight. Error bars, s.d. calculated from night replicates. ***P < 0.001, compared to control in Wilcoxon rank sum test. **f** MA plot comparing control and TBX20 knockdown KYSE510 cells. **g** Heat map of Metascape-enriched clusters. Each cluster contains multiple gene sets to eliminate redundancy. Analysis used genes meeting an FDR *q* < 0.05 threshold.

To understand how the GSDs activated genes defined above are controlled, we focused on TFs because of their broad role in initiating programs key to cell fate and identity in cancer^30^. Among 24 TFs of 208 activated genes with no known role in ESCC, we found expression of TBX20 was associated with TCGA ESCC patients’ outcome in univariate log-rank analysis (*P* = 0.042, log-rank test, Fig. 5a, Supplementary Table 4). We also found that the overall survival of 563 patients with TBX20 mutation was worse than 18,632 patients without TBX20 mutation in TCGA Pan-Cancer Resource (*P* = 9.04 × 10^−4^, log-rank test, Fig. 5a). TBX20 expression was low in normal esophageal epithelial cells, but TBX20 was highly expressed in KYSE450 and KYSE510 cells and TCGA patients’ samples (Fig. 5b, Supplementary Fig. 11). Using a lentiviral-based RNA interference (short hairpin RNA (shRNA)) approach, we knocked down TBX20 in KYSE510 cells and quantified cell growth in vivo (Fig. 5c). As expected, we found that TBX20 depletion significantly suppressed cell growth in vitro (Fig. 5d) and in vivo (Fig. 5e, Supplementary Fig. 12).

To identify the molecules and pathways responsible for the biological effects of TBX20, we performed RNA-seq analysis after knockdown of TBX20 and obtained high correlations between the biological replicates in each experiment (Supplementary Fig. 13). We next performed differential gene expression analysis, which at an FDR of 5%, identified 266 significantly differentially expressed transcripts after TBX20 knockdown in KYSE510. Expression was significantly higher for 112 transcripts (“upregulated abundance”) and significantly lower for 154 transcripts (“downregulated abundance”) in TBX20-depleted KYSE510 cells (Fig. 5f, Supplementary Table 5). To determine enriched DNA motifs at TBX20 regulated genes, motif analysis was performed on differentially expressed transcripts. As expected, the most significantly enriched motif at differentially expressed genes was the TBX20 (Supplementary Fig. 14). Gene ontology and pathway enrichment analysis revealed a significant association between TBX20 and important cancer-related pathways; downregulation was associated with cell cycle (*P* = 5.3 × 10^−08^) and double-strand break repair via homologous recombination (*P* = 9.2 × 10^−07^) in TBX20-deletion KYSE510s (Fig. 5g, Supplementary Table 6). These results indicate that loss of GSDs associated TBX20 and the pathways it controls represent an important hub of tumor control in ESCC.

## Discussion

ESCC is a common malignancy without effective therapy. The hundreds of whole exomes and genomes of ESCCs have been sequenced in the past few years, and numerous key aberrations have been identified^21,31–40^. Although epigenomic alterations, such as changes in DNA methylation^41,42^ and histone modifications^43,44^, also have been observed in ESCCs, it is not clear how they contribute to tumor development. The increasing knowledge on how epigenetic modifications such as H3K27me3 influence patterns of gene expression in cancer holds great promises for understanding biological processes, and developing a novel molecular prognosis biomarker, patient stratifications approaches, and therapeutic intervention strategies.

In this study, we have demonstrated the existence of GSDs was associated with highly conserved and functional essential genes in human esophageal epithelial cells. GSD is mutually exclusive with H3K4me3, H3K4me1, and H3K27ac marks and is linked to gene expression repression. We characterized the GSDs landscape of NE2 cells, and identified that GSDs associated genes are enriched in the processes exclusive to squamous cell carcinoma biology, such as epithelium differentiation and development, as well as cell-fate determination and carcinogenesis. In addition, these GSDs differ from typical silencers, which are preferentially segregated by TAD boundaries, associated with each other via chromatin interactions, and their anchors form links at arbitrary distances and construct multiplex interactions.

In addition to peak breadth encoding information on gene function, we showed that its breadth dysregulation is also a link between H3K27me3 and changes to gene expression. These GSDs are more sensitive to perturbation than typical silencers, and the expression of GSDs associated genes is preferentially affected. In addition, GSDs dysregulation is linked to enhancer activity and gene activation via coordination of H3K4me3 and H3K27ac deposition and DNA hypermethylation of gene bodies. In the human genome, promoter hypermethylation preferentially leads to the silencing of gene expression^45,46^, whereas hypomethylation of high-CpG dense promoters showed the most profound increase in gene expression^47,48^. As we know, most gene bodies have low CpG density and are heavily methylated and its methylation is involved in maintaining gene transcription efficiency^49,50^. Recently, we found gene-body hypermethylation of canyon genes is strongly associated with increased expression of homeobox oncogenes^29^. In this study, we found that loss of H3K27me3 in parallel with hypermethylation of gene body but not promoter within GSDs marked genes is associated with increased gene expression. Therefore, we unveiled the more extent role of epigenetic mechanism we discovered in homeobox oncogenes to GSDs medicated gene-body hypermethylation in tumorigenesis. In addition, we found that GSDs loss upregulated a transcriptional program driven by the TFs of AP1 family. As per previous study, AP1 can exert its oncogenic and antioncogenic effects by regulating genes involved in cell proliferation, differentiation, apoptosis, angiogenesis, and tumor invasion^51^. In esophageal adenocarcinoma, AP1 was identified as a molecular switch to alter gene expression and hence cause cells to adopt a cancer fate^52^.

The biological associations with H3K27me3 dysregulation may be attributed to the function of the tissue and the pathophysiological status of the model in relation to the ongoing gene expression programs. Here, a major new discovery driven by the GSDs dysregulation was the identification of TBX20 as a key regulator in ESCC. We have not only found that high expression of TBX20 is related to the poor prognosis of patients but also we verified the critical role of TBX20 in tumor growth in vivo. Notably, RNA-seq analysis revealed that TBX20 regulates several pathways that are important for tumor progression, including cell cycle^33,53^ and homologous recombination repair^54,55^, which opening a path to identifying new targets for therapy of aggressive cancers and giving support to the relevance of TBX20 network to ESCC.

In conclusion, by exploring epigenetic changes associated with tumorigenesis in ESCC cell line, we discovered that for carcinogenic sets, characterized by loss of grand H3K27me3 domains, their expression levels are raised in cancer. Establishing a catalog of these *cis*-regulatory elements and *trans*-regulatory factors would greatly increase our understanding of the molecular factors that regulate the transcriptional programs in ESCC. The increasing knowledge on how epigenetic modifications such as loss of grand H3K27me3 domains influence patterns of gene expression in cancer holds great promises for understanding biological processes, as well as for their use in prognosis, patient stratifications, and therapeutic intervention.

## Methods

### Cell cultures and siRNA transfection

The human ESCC cell lines KYSE450 and KYSE510 were provided by Dr. Yutaka Shimada (Kyoto University, Kyoto, Japan). The immortalized normal esophageal epithelium NE2 cells were a gift from Dr. Enmin Li (Medical College of Shantou University, Guangdong, China). HEK293T cells were purchased from the ATCC. The ESCC cells were cultured in RPMI-1640 medium with 10% FBS. NE2 cells were cultured in in a 1:1 mixture of defined K-SFM (10744-019, Gibco) and EpiLife medium (MEP1500CA, Gibco). HEK293T cells were cultured in DMEM with 10% FBS. All cells were cultured in cell incubator with 5% CO_2_ at 37 °C. All of the cells were authenticated by short tandem repeat analysis and regularly tested for mycoplasma contamination. siRNAs were purchased from Dharmacon and were transfected following the standard protocol of Lipofectamine 2000 Transfection Reagent. siRNAs used in this study are listed in Supplementary Information, Supplementary Table 7.

### ChIP-seq and ChIP-chip data analysis pipeline

The ChIP experiments were performed following the manufacturer’s protocol (#9003, CST). Briefly, 4 × 10^6^ cells were seed in 10 cm dishes and cultured overnight. Cells were cross-linked with 1% formaldehyde at RT for 10 min, followed by the addition of glycine to terminate cross-linking reaction. After washing with cold PBS, cells were harvested into 1.5 mL EP tubes and fragmented by micrococcal nuclease and ultrasonic wave. The cleared chromatin samples were incubated with antibody overnight at 4 °C, and then incubated with magnetic beads for 2 h. Beads bound with chromatin were then extensively washed and eluted. Eluted chromatin was digested at 65 °C by proteinase K and DNA was purified using columns provided in this kit. The purified ChIP DNA samples and their input DNA were diluted for library construction using Ultra^TM^ II DNA Library Prep Kit (#E7645, NEB). The libraries were sequenced by Illumina HiSeq X Ten Sequencer. ChIP-seq raw reads were aligned to the human reference genome sequence version hg38 using Bowtie2 (version 2.3.4)^56^. For IMR90 cell datasets downloaded in BAM format from the ENCODE databases (https://www.encodeproject.org/), the reads were mapped to the human reference genome version hg38. Significant H3K27me3 peaks were called by using SICER2^57^ with default parameter values, while H3K27ac, H3K4me3, and H3K4me1 were analyzed with all default parameters except -w 200 and -g 400. H3K27me3 peaks were merged within a distance of 2 kb. Identified peaks were annotated by intersection with the reference using BEDTools v2.25.0^58^. We then used the deepTools^59^ bamCompare function to calculate ChIP-seq signal, subtract corresponding background input signal, normalize the number of reads per bin in reads per kilobases per million (RPKM) method, and generate BigWig format files. The input-subtracted peak signal within a region was measured as a RPKM value using bigWigAverageOverBed. The ChIP-seq signal values at each base pair across individual genes were submitted to deepTools to draw profiles and heatmaps.

### RNA-seq and data analysis

Total RNA was extracted with TRIzol reagent (Life Technologies). Complementary DNA libraries were constructed using an Illumina TruSeq RNA Sample Prep kit according to the manufacturer’s protocol. A total of 150 base paired-end reads were sequenced using the Illumina HiSeq X-Ten platform. RNA-seq raw reads were mapped to the human genome version hg38 using STAR-2.6.1^60^ with default parameter values, used the htseq-count function in HTSeq^61^ to calculate read counts for each gene. Transcript abundances at the gene level were calculated as TPM values using RSEM^62^. We utilized the function to generate a BigWig file that contains RNA-seq signal (read density) at each base pair across the genome. The BigWig file was then submitted to IGV^63^ to visualize RNA-seq signal at individual genes. Differential gene expression analysis between groups was performed with the DESeq2 R package^64,65^. The genes with a log_2_ fold change value of >1 and an FDR of <0.05 were considered to be significantly differentially expressed.

### WGBS and data analysis

DNA was extracted using Qiagen DNeasy & Blood Tissue Kit. Fifty nanograms of genomic DNA was used for library preparation. DNA was bisulfite converted with the EZ DNA Methylation Gold Kit (Zymo Research) for use with the TruSeq library preparation methods. A total of 150 base paired-end reads were sequenced using the Illumina HiSeq X-Ten platform. All of the WGBS reads were first processed using Skewer (v0.2.2)^66^ to trim adapter and low-quality reads. Adapter-trimmed reads were mapped to the human genome version hg38 using Bismark v0.20.1^67^ with default parameter values. Methylation BigWig file was generated by iBSTools v1.3.0^29^.

### Hi-C data analysis

We accessed publicly available human IMR90 cell line sequencing reads in FASTQ format from the ENCODE databases, the reads were mapped to the human reference genome version hg38 using Juicer^68^ with default parameter values. TADs and long-range chromatin interactions which contain the genomic location that has been mapped to the human genome version hg19. We converted these data from the hg19 to the hg38 version of the human genome using the tool liftOver (https://genome.ucsc.edu/util.html). The Hi-C maps of regions were visualized using Juicebox v1.11.08^69^.

### Functional enrichment analysis

We used Metascape^70^ for pathway analysis. Pathway with a P-value (accumulative hypergeometric distribution) smaller than 0.01, and an enrichment factor > 1.5 (the enrichment factor is the ratio between the observed counts and the counts expected by chance) are shown. GSEA was conducted through the Bioconductor package “clusterProfiler”^71^.

### Motif analysis

To detect enriched sequence motifs in H3K27ac, H3K4me3, and H3K4me1 peaks associated with altered super silencers, we performed motif analysis using HOMER^72^ with default parameters except size given and mask. The ranks of the most highly enriched TFs are presented according to the *Q*-value.

### Survival analysis

Kaplan–Meier survival curves and log-rank tests were used to compare the survival distribution between the high-expression and low-expression groups through the platform DrBioRight^73^. The survival difference between two groups with or without mutations was visualized using TCGA (https://portal.gdc.cancer.gov).

### Cell proliferation assay

Cell proliferation was measured using CCK-8 reagent at days 0, 2, and 4. Specifically, CCK-8 reagent (Dojindo) was added to the cell culture medium at a ratio of 1:10, and the absorbance was measured at 450 nm after incubation for 1 h at 37 °C.

### Plasmid construction, lentivirus package, and stable cell line generation

shRNA sequences were cloned into pSIH1-puro vector (Addgene). The shRNA sequences targeting TBX20 were as follows: sh1, ACAACAAGAGGTACCGCTA and sh2, TCAAACAGATGGTGTCTTT. Lentivirus was produced using HEK293T cells with the second-generation packaging system psPAX2 (#12260, Addgene) and pMD2.G plasmid (#12259, Addgene). KYSE510 cells were infected with lentivirus in medium supplemented with 8 mg/mL polybrene (Sigma-Aldrich) for 48 h and were then selected with 1 mg/mL puromycin (Sangon Biotech) for 2 weeks.

### Animal experiments

Balb/c-nude mice were purchased from Charles River (USA). All animal protocols were approved by the Animal Care and Use Committee of the Chinese Academy of Medical Sciences Cancer Hospital (Beijing, China). For subcutaneous xenograft, 5 × 10^6^ ESCC cells were injected subcutaneously into 6-week-old female BALB/c-nude mice. Tumor growth was monitored weekly by caliper measurements and calculated individually as: tumor volume = *a* × *b*^2^/2 (*a* represents tumor length and *b* represents tumor width). After 5 weeks, tumors were harvested. Tumor weights were measured and analyzed by Wilcoxon rank sum test.

### Reporting summary

Further information on research design is available in the Nature Research Reporting Summary linked to this article.

## Supplementary information

Supplementary Information

Reporting Summary

## Data Availability

The sequence data obtained in this study have been submitted to the NCBI Sequence Read Archive (SRA) under SRA accession number PRJNA679744. The accession numbers for raw data from public database or the source of processed dataset are indicated in the Supplementary Data (Supplementary Tables 8–10). Human reference genome sequence version hg38 was downloaded from the UCSC Genome Browser website (https://genome.ucsc.edu). All the data used are publicly available at https://github.com/JiangQi1996/H3K27me3. The datasets used and analyzed during the current study are available from the corresponding author on reasonable request.

## Code availability

All the codes used are publicly available at https://github.com/JiangQi1996/H3K27me3.
