# Author Correction: Loss of grand histone H3 lysine 27 trimethylation domains mediated transcriptional activation in esophageal squamous cell carcinoma

**DOI:** 10.1038/s41525-021-00240-6

**Published:** 2021-09-20

**Authors:** Jian Yuan, Qi Jiang, Tongyang Gong, Dandan Fan, Ji Zhang, Fukun Chen, Xiaolin Zhu, Xinyu Wang, Yunbo Qiao, Hongyan Chen, Zhihua Liu, Jianzhong Su

**Affiliations:** 1 School of Biomedical Engineering, School of Ophthalmology & Optometry and Eye Hospital, Wenzhou Medical University, Wenzhou, China; 2 Institute of Biomedical Big Data, Wenzhou Medical University, Wenzhou, China; 3 State Key Laboratory of Molecular Oncology, National Cancer Center/National Clinical Research Center for Cancer/Cancer Hospital, Chinese Academy of Medical Sciences and Peking Union Medical College, Beijing, China; 4 Oujiang Laboratory, Wenzhou, China; 5 Precise Genome Engineering Center, School of Life Sciences, Guangzhou University, Guangzhou, China

**Keywords:** Oesophageal cancer, Cancer genomics

Correction to: *npj Genomic Medicine* 10.1038/s41525-021-00232-6, published online 11 August 2021

The original version of this Article contained errors in the author affiliations.

Tongyang Gong, Hongyan Chen, and Zhihua Liu were incorrectly associated with Oujiang Laboratory, Wenzhou, China.

This has now been corrected in both the PDF and HTML versions of the Article.

