# Supplementary material for: Loss of grand histone H3 lysine 27 trimethylation domains mediated transcriptional activation in esophageal squamous cell carcinoma
Source: NPJ Genom Med. 2021 Aug 11;6:65. doi: 10.1038/s41525-021-00232-6 (PMC8358006; doi:10.1038/s41525-021-00232-6)
Supplement: Supplementary file 1 — Supplementary Information [file 41525_2021_232_MOESM1_ESM.pdf]

Supplementary Table 1 Enrichments from the Metascape annotation tool for human NE2 cells

Description	GO	LogP	Enrichment
pattern specification process	GO:0007389	-42	5.4
embryonic organ development	GO:0048568	-35	4.9
cell fate commitment	GO:0045165	-29	5.8
endocrine system development	GO:0035270	-18	6.7
tissue morphogenesis	GO:0048729	-17	3
developmental process involved in reproduction	GO:0003006	-15	2.7
cardiac ventricle morphogenesis	GO:0003208	-14	7.8
cell fate determination	GO:0001709	-13	10
cell fate commitment	GO:0045165	-13	4
tissue morphogenesis	GO:0048729	-12	2.6
digestive system development	GO:0055123	-11	4.6
epithelial tube morphogenesis	GO:0060562	-11	3.3
embryonic organ development	GO:0048568	-9.5	2.8
pattern specification process	GO:0007389	-8.9	2.7
cell fate commitment	GO:0045165	-6.9	2.9
tissue morphogenesis	GO:0048729	-6.2	2.1
amine transport	GO:0015837	-5.9	4.2
tissue morphogenesis	GO:0048729	-5.8	2
Transcriptional misregulation in cancer	hsa05202	-5.7	3
amine transport	GO:0015837	-5.7	4
cell fate commitment	GO:0045165	-5.7	2.7
epithelial tube morphogenesis	GO:0060562	-5.5	2.5
Pathways in cancer	hsa05200	-5.5	2.3
pattern specification process	GO:0007389	-5.5	2.2
embryonic organ development	GO:0048568	-5.5	2.2
epithelial cell proliferation	GO:0050673	-5.4	2.2
PI3K-Akt signaling pathway	hsa04151	-5.4	2.3
PI3K-Akt signaling pathway	hsa04151	-5.3	2.4
PI3K-Akt signaling pathway	hsa04151	-5.1	2.3
amine transport	GO:0015837	-5.1	3.8
Notch signaling pathway	GO:0007219	-5	2.8
Pathways in cancer	hsa05200	-4.8	2
PI3K-Akt signaling pathway	hsa04151	-4.8	2.2
developmental process involved in reproduction	GO:0003006	-4.8	1.9
endocrine system development	GO:0035270	-4.7	3.4
PI3K-Akt signaling pathway	hsa04151	-4.7	2.3

cell fate commitment	GO:0045165	-4.6	2.5
PI3K-Akt signaling pathway	hsa04151	-4.5	2.2
Notch signaling pathway	GO:0007219	-4.3	2.7
Wnt signaling pathway	GO:0016055	-4.3	1.9
muscle cell fate commitment	GO:0042693	-4.2	8
PI3K-Akt signaling pathway	hsa04151	-4.2	2.2
embryonic organ development	GO:0048568	-4.2	2.1
tissue morphogenesis	GO:0048729	-4.2	1.8
Hippo signaling pathway	hsa04390	-4	2.8
pattern specification process	GO:0007389	-4	2
developmental process involved in reproduction	GO:0003006	-4	1.8
embryonic organ development	GO:0048568	-3.9	2
cell fate commitment	GO:0045165	-3.9	2.3
developmental process involved in reproduction	GO:0003006	-3.9	1.8
embryonic organ development	GO:0048568	-3.8	2
developmental process involved in reproduction	GO:0003006	-3.7	1.8
developmental process involved in reproduction	GO:0003006	-3.7	1.7
cardiac ventricle morphogenesis	GO:0003208	-3.6	3.9
tissue morphogenesis	GO:0048729	-3.6	1.7
endocrine system development	GO:0035270	-3.5	2.9
developmental process involved in reproduction	GO:0003006	-3.5	1.8
digestive system development	GO:0055123	-3.4	2.7
PI3K-Akt signaling pathway	hsa04151	-3.4	2
amine transport	GO:0015837	-3.4	3.2
tissue morphogenesis	GO:0048729	-3.4	1.7
tissue morphogenesis	GO:0048729	-3.4	1.8
tissue morphogenesis	GO:0048729	-3.4	1.8
epithelial tube morphogenesis	GO:0060562	-3.3	2.1
canonical Wnt signaling pathway	GO:0060070	-3.3	2
cardiac ventricle morphogenesis	GO:0003208	-3.3	3.5
amine transport	GO:0015837	-3.3	3.1
cell fate commitment	GO:0045165	-3.3	2.2
epithelial tube morphogenesis	GO:0060562	-3.2	2.1
epithelial tube morphogenesis	GO:0060562	-3.2	2.1
Hippo signaling pathway	hsa04390	-3.2	2.5
Transcriptional misregulation in cancer	hsa05202	-3.2	2.4

PI3K-Akt signaling pathway	hsa04151	-3.2	2
developmental process involved in reproduction	GO:0003006	-3.2	1.7
tissue morphogenesis	GO:0048729	-3.2	1.7
canonical Wnt signaling pathway	GO:0060070	-3.1	2
cell fate commitment	GO:0045165	-3	2.1
developmental process involved in reproduction	GO:0003006	-3	1.7
epithelial tube morphogenesis	GO:0060562	-2.9	2
Pathways in cancer	hsa05200	-2.9	1.7
canonical Wnt signaling pathway	GO:0060070	-2.9	2
pattern specification process	GO:0007389	-2.9	1.8
digestive system development	GO:0055123	-2.8	2.5
Pathways in cancer	hsa05200	-2.8	1.7
cardiac ventricle morphogenesis	GO:0003208	-2.8	3.3
embryonic organ development	GO:0048568	-2.8	1.8
cell fate commitment	GO:0045165	-2.8	2.1
developmental process involved in reproduction	GO:0003006	-2.8	1.6
developmental process involved in reproduction	GO:0003006	-2.8	1.6
epithelial cell fate commitment	GO:0072148	-2.6	6.7
PI3K-Akt signaling pathway	hsa04151	-2.6	1.8
pattern specification process	GO:0007389	-2.6	1.8
epithelial tube morphogenesis	GO:0060562	-2.5	1.9
pattern specification process	GO:0007389	-2.5	1.7
amine transport	GO:0015837	-2.5	2.7
Pathways in cancer	hsa05200	-2.4	1.6
PI3K-Akt signaling pathway	hsa04151	-2.4	1.8
stem cell fate commitment	GO:0048865	-2.4	8.6
stem cell fate commitment	GO:0048865	-2.4	8.4
cell fate determination	GO:0001709	-2.3	3.8
muscle cell fate commitment	GO:0042693	-2.3	5.6
muscle cell fate commitment	GO:0042693	-2.3	5.5
Pathways in cancer	hsa05200	-2.3	1.6
PI3K-Akt signaling pathway	hsa04151	-2.3	1.8
PI3K-Akt signaling pathway	hsa04151	-2.3	1.8
embryonic organ development	GO:0048568	-2.3	1.7
tissue morphogenesis	GO:0048729	-2.3	1.6
epithelial tube morphogenesis	GO:0060562	-2.2	1.8
PI3K-Akt signaling pathway	hsa04151	-2.2	1.7

developmental process involved in reproduction	GO:0003006	-2.1	1.5
Pathways in cancer	hsa05200	-2	1.6

Supplementary Table 2. Enriched GO terms of GSD targeted genes and GSD marked genes

GO	Description	-log10(p_GSD)	enrichment_GSD	zscore_GSD	-log10(Q_GSD)	-log10(p_target)	enrichment_target	zscore_target	-log10(Q_target)
GO:0000904	cell morphogenesis involved in differentiation	10	2.3	7.4	7.6	3	2.1	3.7	0.33
GO:0001501	skeletal system development	34	4.5	16	30	6.4	3.4	6.4	2.4
GO:0001525	angiogenesis	3.1	1.7	3.5	1.7	2.2	2	2.9	0.074
GO:0001568	blood vessel development	4	1.7	4.2	2.4	2.1	1.8	2.8	0.056
GO:0001701	in utero embryonic development	3.1	1.9	3.6	1.7	2.3	2.4	3.2	0.083
GO:0001963	synaptic transmission, dopaminergic	2.4	4.7	3.9	1.1	2.3	8.5	4.5	0.083
GO:0002009	morphogenesis of an epithelium	7.1	2.3	6.1	5.1	2.2	2.1	2.9	0.074
GO:0002062	chondrocyte differentiation	7.3	4.4	7.2	5.2	4	5.6	5.6	0.68
GO:0002063	chondrocyte development	2.8	4.7	4.3	1.5	3.1	9.6	5.6	0.4
GO:0002065	columnar/cuboidal epithelial cell differentiation	7.2	4.2	7.1	5.2	2.3	3.8	3.6	0.083
GO:0003002	regionalization	35	5.5	17	31	3.6	3	4.4	0.61
GO:0003014	renal system process	3.3	2.8	4.2	1.9	2.2	3.7	3.5	0.078

GO:0003073	regulation of systemic arterial blood pressure	2.9	2.9	3.8	1.5	2.1	4	3.4	0.057
GO:0006873	cellular ion homeostasis	6.7	2.1	5.8	4.7	2.4	2	3.2	0.11
GO:0006875	cellular metal ion homeostasis	6.4	2.2	5.7	4.5	2.3	2.1	3.1	0.083
GO:0007264	small GTPase mediated signal transduction	2.6	1.7	3.2	1.3	2.5	2.3	3.3	0.13
GO:0007389	pattern specification process	40	5.2	18	36	3	2.5	3.8	0.33
GO:0007409	axonogenesis	9.2	2.6	7.3	6.9	2.8	2.4	3.5	0.2
GO:0007519	skeletal muscle tissue development	5.9	3.4	6	4	2.3	3.4	3.5	0.083
GO:0007626	locomotory behavior	8.8	3.7	7.7	6.6	2.3	3.1	3.4	0.083
GO:0009792	embryo development ending in birth or egg hatching	18	3	11	15	5.1	2.8	5.3	1.5
GO:0009952	anterior/posterior pattern specification	29	6.4	16	25	3.8	3.9	4.9	0.68
GO:0009991	response to extracellular stimulus	2.1	1.6	2.7	0.91	3	2.4	3.8	0.33
GO:0010769	regulation of cell morphogenesis	2.2	1.8	2.8	0.95	2.6	2.7	3.5	0.14

	involved in differentiation								
GO:0010810	regulation of cell-substrate adhesion	3.8	2.4	4.3	2.2	2.1	2.8	3.1	0.057
GO:0010817	regulation of hormone levels	9.9	2.6	7.5	7.6	3	2.4	3.7	0.33
GO:0015698	inorganic anion transport	2.4	2.2	3.1	1.1	2.7	3.5	3.8	0.15
GO:0015850	organic hydroxy compound transport	3.3	2.2	3.9	1.8	2.1	2.6	3	0.047
GO:0016055	Wnt signaling pathway	3.6	1.8	3.9	2.1	2.4	2.2	3.2	0.11
GO:0021545	cranial nerve development	7.2	6.4	7.9	5.1	3.3	7.5	5.4	0.43
GO:0021554	optic nerve development	2.9	7.8	5	1.5	3.2	18	6.9	0.42
GO:0021602	cranial nerve morphogenesis	7	8.7	8.4	5	2.2	8	4.3	0.076
GO:0021675	nerve development	7.2	5.1	7.4	5.1	3.2	5.8	4.9	0.42
GO:0021915	neural tube development	2.3	2.2	3	1	2.3	3.3	3.4	0.083
GO:0022604	regulation of cell morphogenesis	2.5	1.7	3.1	1.2	2.6	2.3	3.4	0.14
GO:0023061	signal release	11	2.9	8.3	8.9	2.4	2.3	3.2	0.1
GO:0030003	cellular cation homeostasis	5.9	2	5.4	4	2.5	2.1	3.2	0.13
GO:0030198	extracellular matrix organization	2.3	1.7	2.9	1	2.6	2.5	3.5	0.14
GO:0030326	embryonic limb morphogenesis	13	5.6	10	10	2.9	4.2	4.2	0.25

GO:0030510	regulation of BMP signaling pathway	4.1	3.5	5	2.5	2.1	4.1	3.5	0.061
GO:0031069	hair follicle morphogenesis	2.4	4.5	3.8	1.1	2.3	8.2	4.4	0.083
GO:0031589	cell-substrate adhesion	4.7	2.2	4.8	3	2.1	2.3	3	0.057
GO:0031667	response to nutrient levels	2.6	1.7	3.1	1.2	3.3	2.5	4	0.45
GO:0032386	regulation of intracellular transport	2.2	1.6	2.8	0.96	2.2	2.3	3.1	0.08
GO:0035107	appendage morphogenesis	14	5.2	11	11	3.8	4.6	5.1	0.68
GO:0035108	limb morphogenesis	14	5.2	11	11	3.8	4.6	5.1	0.68
GO:0035113	embryonic appendage morphogenesis	13	5.6	10	10	2.9	4.2	4.2	0.25
GO:0043009	chordate embryonic development	18	3	11	15	5.3	2.9	5.5	1.6
GO:0043408	regulation of MAPK cascade	5.8	1.9	5.3	3.9	2.3	1.9	3	0.083
GO:0044057	regulation of system process	22	3.4	12	19	2.1	2	2.8	0.056
GO:0045761	regulation of adenylate cyclase activity	2.1	3.9	3.4	0.87	2.1	7.2	4	0.057
GO:0046879	hormone secretion	6.7	2.7	6.1	4.7	2	2.4	2.9	0.034
GO:0048514	blood vessel morphogenesis	3.4	1.7	3.7	1.9	2.3	2	3	0.083

GO:0048536	spleen development	2.7	4.5	4.1	1.4	2	6.8	3.9	0.034
GO:0048562	embryonic organ morphogenesis	30	5.5	16	26	3.7	3.3	4.6	0.65
GO:0048568	embryonic organ development	25	4.2	14	22	4	2.9	4.6	0.68
GO:0048589	developmental growth	12	2.6	8.3	9.3	2.1	2	2.9	0.07
GO:0048598	embryonic morphogenesis	32	4.1	16	29	5.8	3.1	5.9	2
GO:0048667	cell morphogenesis involved in neuron differentiation	8.8	2.4	7	6.6	2.6	2.2	3.3	0.14
GO:0048704	embryonic skeletal system morphogenesis	17	7.5	13	14	3.6	5.5	5.2	0.61
GO:0048705	skeletal system morphogenesis	26	6	15	23	6.5	5.1	7.1	2.4
GO:0048706	embryonic skeletal system development	20	7	14	17	3.5	4.7	4.9	0.58
GO:0048729	tissue morphogenesis	12	2.6	8.5	9.8	2.2	2	2.9	0.074
GO:0048730	epidermis morphogenesis	2.1	3.9	3.4	0.87	2.1	7.2	4	0.057
GO:0048736	appendage development	14	4.9	11	12	3.9	4.2	5	0.68
GO:0048812	neuron projection morphogenesis	11	2.5	7.7	8.2	2.2	2	2.9	0.074
GO:0048858	cell projection morphogenesis	10	2.4	7.7	8.1	2.1	1.9	2.8	0.047

GO:0048863	stem cell differentiation	5.6	2.7	5.5	3.8	3.1	3.2	4.1	0.4
GO:0050804	modulation of chemical synaptic transmission	14	3.2	9.7	12	2.6	2.4	3.4	0.14
GO:0050886	endocrine process	2.7	3	3.7	1.4	2.3	4.6	3.8	0.083
GO:0051047	positive regulation of secretion	3.6	2	4	2.1	2.4	2.5	3.3	0.1
GO:0051056	regulation of small GTPase mediated signal transduction	3.2	2	3.8	1.8	2.9	2.8	3.8	0.28
GO:0051146	striated muscle cell differentiation	2.7	2	3.4	1.4	2.2	2.6	3.2	0.08
GO:0051216	cartilage development	12	4.4	9.6	9.8	3.6	3.9	4.7	0.61
GO:0051899	membrane depolarization	4.9	3.9	5.6	3.1	2.2	4.2	3.5	0.074
GO:0055065	metal ion homeostasis	7.5	2.2	6.3	5.4	2.6	2.1	3.3	0.14
GO:0055080	cation homeostasis	7.2	2.1	6.1	5.2	3.1	2.2	3.7	0.4
GO:0060173	limb development	14	4.9	11	12	3.9	4.2	5	0.68
GO:0060348	bone development	8	3.5	7.2	5.8	3.4	3.8	4.6	0.53
GO:0060349	bone morphogenesis	7.6	4.8	7.6	5.5	2.1	4.1	3.4	0.057
GO:0060350	endochondral bone morphogenesis	4.3	4.6	5.4	2.6	2.2	5.6	3.9	0.083
GO:0060538	skeletal muscle organ development	7.2	3.6	6.9	5.2	2.2	3.2	3.3	0.074
GO:0060986	endocrine hormone secretion	2.6	3.8	3.8	1.3	2.5	6.5	4.4	0.13

GO:0061448	connective tissue development	12	3.7	9	9.3	3.7	3.5	4.7	0.64
GO:0061564	axon development	10	2.6	7.6	7.7	2.4	2.2	3.1	0.092
GO:0071407	cellular response to organic cyclic compound	5	2.1	4.9	3.3	2.9	2.4	3.7	0.27
GO:0072507	divalent inorganic cation homeostasis	5.8	2.2	5.4	4	2.1	2.1	2.9	0.057
GO:0090257	regulation of muscle system process	6.9	3	6.4	4.9	2.2	2.7	3.1	0.074
GO:0090287	regulation of cellular response to growth factor stimulus	2.8	1.9	3.4	1.4	2.1	2.5	3	0.057
GO:0098771	inorganic ion homeostasis	7.4	2.1	6.1	5.3	3	2.2	3.7	0.34
GO:0099177	regulation of trans-synaptic signaling	14	3.2	9.7	12	2.6	2.4	3.4	0.14
GO:0099536	synaptic signaling	20	3	11	17	2.1	1.9	2.7	0.047
GO:0120039	plasma membrane bounded cell projection morphogenesis	10	2.4	7.6	7.8	2.1	1.9	2.8	0.056
GO:0198738	cell-cell signaling by wnt	3.6	1.8	3.9	2	2.4	2.2	3.2	0.11
GO:1901568	fatty acid derivative metabolic process	2.4	2.2	3.1	1.1	2.1	3.1	3.2	0.057
GO:1903305	regulation of regulated secretory pathway	4	2.8	4.6	2.4	2.2	3.3	3.3	0.078

GO:1903530	regulation of secretion by cell	7.1	2.1	6	5.1	2.3	2	3.1	0.085
GO:1903532	positive regulation of secretion by cell	2.3	1.8	2.9	1	2.7	2.7	3.6	0.19
GO:1905114	cell surface receptor signaling pathway involved in cell-cell signaling	7	2.2	6	5	3.2	2.3	3.9	0.42
M5884	NABA CORE MATRISOME	3.2	2.1	3.8	1.7	3	3.1	4	0.33
R-HSA-112314	Neurotransmitter receptors and postsynaptic signal transmission	9.1	3.7	7.9	6.9	2.8	3.4	3.9	0.23
R-HSA-112315	Transmission across Chemical Synapses	14	4	10	11	2.5	2.8	3.5	0.13
R-HSA-375165	NCAM signaling for neurite out-growth	4.5	4.4	5.5	2.8	2	4.9	3.5	0.037
R-HSA-382551	Transport of small molecules	12	2.5	8.5	9.8	4	2.4	4.5	0.68
R-HSA-425366	Transport of bile salts and organic acids, metal ions and amine compounds	11	6.2	9.8	8.5	2.3	4.5	3.7	0.083
R-HSA-425407	SLC-mediated transmembrane transport	12	3.8	9.1	9.4	2.8	3.1	3.8	0.2
R-HSA-983712	Ion channel transport	3.8	2.6	4.4	2.2	2.5	3.3	3.6	0.13

Supplementary Table 3. GSDs activated gene in ESCC

gene	symbol	LFC_KYSE450	padj_KYSE450	Signal_NE2	Signal_KYSE450	length_NE2
ENSG00000004846	ABCB5	3.666905993	0.038851885	6.22539	0.1	103.8
ENSG00000153093	ACOXL	5.360972992	3.34E-09	6.8972	0.0686602	98.2
ENSG00000077522	ACTN2	8.859038703	3.04E-08	3.74561	0.1	62.4
ENSG00000008277	ADAM22	5.955799199	9.93E-45	3.81285	0.1	74.4
ENSG00000114948	ADAM23	8.871021467	5.77E-10	3.51169	0.1	58.2
ENSG00000173157	ADAMTS20	11.1591661	5.46E-15	2.91208	0.1	57
ENSG00000164742	ADCY1	6.626539949	1.64E-128	5.76464	0.1	59
ENSG00000075340	ADD2	3.02832669	1.10E-67	4.96407	0.1	148.4
ENSG00000164199	ADGRV1	7.896319425	1.68E-44	1.35562	0.1	56.4
ENSG00000165092	ALDH1A1	4.844695784	4.52E-47	4.30643	0.918915	69.8
ENSG00000180318	ALX1	5.361355424	6.93E-42	9.21158	0.1	79.2
ENSG00000052850	ALX4	2.669926203	0.001751399	4.66411	0.559585	93.6
ENSG00000131620	ANO1	11.39539269	9.45E-16	2.86519	0.1	60.8
ENSG00000136250	AOAH	7.503175186	6.63E-06	4.50322	0.1	81.6
ENSG00000103723	AP3B2	3.020344418	3.04E-38	2.18277	0.1	57.2
ENSG00000165309	ARMC3	6.034845302	0.009412113	5.42357	0.1	59.2
ENSG00000169126	ARMC4	2.111492961	0.000780103	2.84843	0.1	129.8
ENSG00000064270	ATP2C2	10.61179321	8.86E-14	4.49212	0.1	60.4
ENSG00000107518	ATRNL1	4.378047964	4.79E-21	2.86442	0.1	56.8
ENSG00000043039	BARX2	9.389295408	6.10E-11	5.48976	1.09337	67.4
ENSG00000064787	BCAS1	5.708137033	1.65E-11	2.95467	0.1	57.2
ENSG00000101144	BMP7	13.39037985	3.76E-38	5.04941	0.1	93
ENSG00000125999	BPIFB1	4.460678251	0.012844206	4.12022	2.92915	109.6
ENSG00000125462	C1orf61	8.618737518	2.39E-16	18.5668	3.36634	74.4
ENSG00000173557	C2orf70	4.982913261	1.80E-06	10.9895	8.56664	179.6
ENSG00000154274	C4orf19	5.703962027	0.000193988	1.46301	0.335407	60.6
ENSG00000157388	CACNA1D	9.642189232	1.90E-11	4.77164	0.1	169.6
ENSG00000153956	CACNA2D1	13.52050203	5.99E-20	4.81029	0.1	222.8
ENSG00000075461	CACNG4	11.31308337	2.97E-14	6.64083	0.1	72.4
ENSG00000152495	CAMK4	4.678772802	1.10E-49	3.71942	0.1	115.4
ENSG00000101331	CCM2L	7.619870038	5.64E-06	12.0197	1.77655	151.2
ENSG00000113361	CDH6	5.123285868	0.014446369	2.9526	0.0113392	141.6
ENSG00000197748	CFAP43	2.910871787	1.65E-10	5.04234	0.1	57.8
ENSG00000016391	CHDH	5.104302022	2.35E-60	4.5006	0.1	169.6
ENSG00000090539	CHRD	7.522772803	1.97E-05	8.76655	1.88072	60.8
ENSG00000175344	CHRNA7	7.152373654	5.50E-06	4.20418	1.04169	74.2
ENSG00000170293	CMTM8	3.419991053	5.45E-26	2.61629	0.1	101.8

ENSG00000119946	CNNM1	11.15194438	7.13E-14	4.15524	0.592812	55.4
ENSG00000018236	CNTN1	14.27005723	1.34E-83	2.40765	0.1	63.2
ENSG00000188517	COL25A1	4.78950833	0.02037732	2.80495	0.1	84.4
ENSG00000198756	COLGALT2	3.47809713	3.05E-05	6.36001	0.1	57.4
ENSG00000214575	CPEB1	5.546154926	3.16E-12	7.00235	0.1	57.2
ENSG00000117322	CR2	11.26044494	6.04E-14	15.3345	0.1	69.8
ENSG00000077063	CTTNBP2	5.607250797	1.21E-07	2.42178	1.19598	72
ENSG00000168772	CXXC4	8.351137367	1.58E-07	5.23272	0.1	61.6
ENSG00000170959	DCDC1	5.063432308	1.56E-06	2.49311	0.0208379	80.4
ENSG00000133083	DCLK1	5.768890106	1.20E-43	1.56516	0.1	102.8
ENSG00000105880	DLX5	9.607650038	3.48E-10	26.6935	0.1	115.8
ENSG00000006377	DLX6	10.63722833	1.28E-12	24.4724	0.1	115.8
ENSG00000137090	DMRT1	7.725236801	1.38E-06	5.84998	0.979067	160.6
ENSG00000173253	DMRT2	11.8704776	5.16E-17	2.25237	0.1	160.6
ENSG00000064218	DMRT3	8.789287409	2.71E-08	8.96019	1.44817	160.6
ENSG00000105877	DNAH11	1.178255281	0.000639741	2.8575	0.1	60.6
ENSG00000157851	DPYSL5	8.473031843	2.02E-08	9.30384	3.90021	64.8
ENSG00000158560	DYNC1I1	6.077413865	1.60E-42	7.22825	0.1	171.8
ENSG00000164176	EDIL3	8.638868221	1.27E-38	2.18794	0.1	70.6
ENSG00000155849	ELMO1	4.120221768	0.030773008	8.33251	0.321443	207.6
ENSG00000110675	ELMOD1	5.956977382	0.008498214	8.51655	1.23342	63
ENSG00000170370	EMX2	8.15874517	1.29E-06	19.5471	4.99699	127
ENSG00000163064	EN1	11.65297665	6.04E-15	16.8185	0.1	101.8
ENSG00000164778	EN2	9.907838443	7.83E-11	23.3478	0.1	68.6
ENSG00000001561	ENPP4	7.005582654	1.50E-26	8.32564	0.863162	70.8
ENSG00000112796	ENPP5	3.351362332	3.78E-08	9.00866	0.1	70.8
ENSG00000086289	EPDR1	8.435475976	3.90E-42	3.07103	1.81287	73
ENSG00000070886	EPHA8	9.573711147	6.71E-10	6.46042	2.39861	91.6
ENSG00000157554	ERG	4.078851486	1.47E-32	2.97325	0.1	65.4
ENSG00000204334	ERICH2	2.991176062	0.001046763	7.56993	0.1	137.8
ENSG00000196482	ESRRG	6.443402871	9.08E-05	1.89938	0.1	130.4
ENSG00000112319	EYA4	6.646848347	1.93E-19	4.69153	0.1	184.2
ENSG00000150510	FAM124A	4.15323992	8.68E-09	6.71638	0.1	101.8
ENSG00000135842	FAM129A	5.703989906	0	7.8376	0.1	70.6
ENSG00000139438	FAM222A	3.70455679	6.37E-12	4.13033	0.1	70.6
ENSG00000128610	FEZF1	6.397955738	0.002841704	22.796	0.1	88.8
ENSG00000137441	FGFBP2	8.686207304	3.42E-09	2.73099	0.1	125.2
ENSG00000261308	FIGNL2	3.857122422	0.000628303	9.53233	7.16799	72
ENSG00000155816	FMN2	7.733228438	6.15E-06	5.19508	0.897976	163.4
ENSG00000103241	FOXF1	5.229186163	0.013132687	16.0272	9.16131	140.4

ENSG00000176165	FOXG1	7.644470331	1.90E-17	14.5236	0.1	69.6
ENSG00000139445	FOXN4	5.491553254	0.025001997	6.41151	1.19811	75.8
ENSG00000128573	FOXP2	8.454800375	5.02E-22	3.39464	0.1	199.8
ENSG00000164946	FREM1	7.23805498	3.51E-05	3.25345	0.940423	85.8
ENSG00000150893	FREM2	9.85791832	1.02E-10	4.75032	0.1	117.6
ENSG00000128683	GAD1	9.906691933	3.57E-11	8.44352	0.1	137.8
ENSG00000179348	GATA2	2.667962767	0.003059309	15.5239	0.1	81.2
ENSG00000107485	GATA3	7.069949595	9.68E-06	11.6749	2.3508	63.4
ENSG00000171766	GATM	5.346630995	3.49E-67	10.4835	0.853031	77.4
ENSG00000115271	GCA	1.744773431	1.17E-24	4.11756	0.1	75.2
ENSG00000151892	GFRA1	6.716686099	0.000440083	4.96206	1.84622	171.6
ENSG00000170837	GPR27	11.51594939	9.35E-15	11.4362	1.23085	130.8
ENSG00000155974	GRIP1	2.363028484	1.01E-36	4.4138	0.1	121.6
ENSG00000164107	HAND2	7.475739811	2.94E-05	16.323	0.665803	68.8
ENSG00000135547	HEY2	7.153054056	8.91E-06	12.4001	5.25581	62.2
ENSG00000188816	HMX2	7.497296135	2.67E-05	19.6538	0.1	74.8
ENSG00000253293	HOXA10	3.869352642	1.89E-130	2.38442	0.1	103.8
ENSG00000005073	HOXA11	2.387453391	4.25E-36	6.27155	0.1	103.8
ENSG00000106031	HOXA13	11.46817256	1.19E-14	20.5866	0.1	103.8
ENSG00000078399	HOXA9	8.574500595	1.58E-66	2.29693	0.1	103.8
ENSG00000108511	HOXB6	1.10993328	0.049255925	17.7332	7.5141	111
ENSG00000260027	HOXB7	2.210839321	1.15E-57	20.6069	5.69643	111
ENSG00000170689	HOXB9	7.408923122	1.11E-06	23.4882	10.0961	111
ENSG00000123364	HOXC13	3.775171209	7.37E-41	9.5133	1.6835	94
ENSG00000128710	HOXD10	7.141094595	5.60E-06	18.9637	0.420487	156.6
ENSG00000128713	HOXD11	4.48850437	1.04E-07	14.0434	0.1	156.6
ENSG00000128714	HOXD13	9.90048397	1.17E-10	20.4714	0.1	156.6
ENSG00000170166	HOXD4	6.104264145	0.007557655	20.2917	0.289308	156.6
ENSG00000175879	HOXD8	10.29594437	1.14E-11	14.0269	0.189888	156.6
ENSG00000128709	HOXD9	3.791163261	0.038109532	20.6217	0.1	156.6
ENSG00000172201	ID4	11.65636772	3.59E-15	7.66613	0.1	107
ENSG00000163083	INHBB	6.186757699	1.06E-07	9.51213	3.16144	88.4
ENSG00000109944	JHY	2.028826246	0.000965847	5.30527	0.470239	95
ENSG00000169282	KCNAB1	4.043139388	0.001511274	1.46819	0.1	96.8
ENSG00000109265	KIAA1211	8.022446822	1.13E-13	4.49871	0.1	70.4
ENSG00000157404	KIT	9.954496789	8.54E-11	6.52294	2.31533	147.4
ENSG00000266265	KLF14	3.699507162	0.034884087	23.6262	5.02616	60
ENSG00000167768	KRT1	14.48021513	8.22E-21	5.62388	2.2241	62.4
ENSG00000189182	KRT77	11.89693275	1.56E-15	6.99709	1.11712	62.4
ENSG00000136167	LCP1	12.65372207	1.07E-130	7.06562	0.1	136.4

ENSG00000138795	LEF1	7.692078424	1.34E-12	3.11367	0.1	61.6
ENSG00000153012	LGI2	5.00570766	1.46E-07	6.48254	3.03214	57.4
ENSG00000162624	LHX8	6.906018872	0.000385206	6.70087	0.1	60.2
ENSG00000136944	LMX1B	7.204361582	2.56E-06	10.0075	3.06158	154.6
ENSG00000203782	LOR	9.811181619	2.12E-10	25.252	7.1169	78.4
ENSG00000175445	LPL	12.87578827	3.47E-18	4.97055	0.945382	69.2
ENSG00000081479	LRP2	6.436006192	6.34E-09	4.74514	0.3094	107
ENSG00000033122	LRRC7	8.590280203	6.12E-09	1.30405	0.1	63.2
ENSG00000170382	LRRN2	4.654267273	0.005146965	13.5086	5.00373	123
ENSG00000144893	MED12L	2.075975	1.04E-19	1.31171	0.1	103
ENSG00000081189	MEF2C	7.00311405	1.46E-68	3.90768	0.1	120.4
ENSG00000157890	MEGF11	3.870775488	0.002797841	5.34727	1.98129	102.6
ENSG00000153208	MERTK	3.053573363	2.29E-10	3.28101	0.39778	55.8
ENSG00000150051	MKX	6.959558757	0.000209604	2.68376	0.1	129.8
ENSG00000066382	MPPED2	13.97105914	2.73E-21	6.58602	0.1	208.4
ENSG00000120149	MSX2	2.790825969	1.55E-34	2.17842	0.1	57
ENSG00000169550	MUC15	9.372226358	1.41E-09	5.20098	1.01095	84.6
ENSG00000101306	MYLK2	1.621883768	0.018906878	3.38075	0.1	63
ENSG00000167306	MYO5B	4.830959718	8.35E-187	5.40846	0.1	218.8
ENSG00000172915	NBEA	2.376142621	7.53E-07	1.08919	0.0335245	77.6
ENSG00000136352	NKX2-1	12.1476027	2.88E-16	18.5157	0.1	190.2
ENSG00000183072	NKX2-5	7.836978506	4.16E-31	19.811	0.1	72
ENSG00000136327	NKX2-8	3.573257267	1.47E-19	3.3123	0.1	190.2
ENSG00000167034	NKX3-1	1.057685682	1.38E-05	2.26024	0.1	68.6
ENSG00000163623	NKX6-1	4.439217384	7.09E-11	2.87623	0.1	56.4
ENSG00000112333	NR2E1	10.03167381	2.56E-11	11.6594	0.1	54.8
ENSG00000151623	NR3C2	5.030767965	1.54E-12	2.04427	0.1	132
ENSG00000148053	NTRK2	13.73767602	1.25E-20	2.169	0.1	104.4
ENSG00000205927	OLIG2	7.153817889	0.000128285	22.7876	3.81825	230.4
ENSG00000169856	ONECUT1	4.359871877	0.034066044	8.56603	5.20506	66.4
ENSG00000007372	PAX6	6.346455979	0.002930214	12.9665	2.49475	115.2
ENSG00000009709	PAX7	10.66618792	1.14E-12	10.0693	2.46958	82.8
ENSG00000186472	PCLO	4.035365376	4.39E-17	3.431	0.1	100.4
ENSG00000172572	PDE3A	9.697388004	2.37E-10	3.95229	0.1	95.2
ENSG00000155629	PIK3AP1	6.244935514	3.14E-157	2.33995	0.1	60
ENSG00000164093	PITX2	8.718021814	1.99E-16	17.2621	0.1	97.4
ENSG00000120278	PLEKHG1	3.280750253	3.73E-25	4.19905	0.1	114
ENSG00000184486	POU3F2	6.131118946	0.00183968	15.1691	11.0401	84.8
ENSG00000106536	POU6F2	9.94708438	6.34E-11	8.47239	0.1	247.4
ENSG00000143847	PPFIA4	1.49989355	2.76E-10	5.21132	0.1	68.2

ENSG00000158528	PPP1R9A	6.883683556	2.42E-33	6.04327	0.1	105.6
ENSG00000061455	PRDM6	3.773672464	1.72E-08	7.16197	0.1	78
ENSG00000185532	PRKG1	3.348034911	5.31E-09	3.22782	0.1	131.8
ENSG00000203783	PRR9	8.190225706	1.08E-06	14.9131	5.14976	78.4
ENSG00000166450	PRTG	1.849610953	1.40E-07	4.37298	0.1	60.2
ENSG00000204179	PTPN20	9.839547602	8.96E-21	1.3901	0.1	149.2
ENSG00000154917	RAB6B	1.651692711	0.007621684	3.5169	1.45955	68
ENSG00000157927	RADIL	8.655855028	2.59E-09	3.43049	0.1	58.2
ENSG00000113319	RASGRF2	6.969141594	1.48E-33	3.84857	0.1	72.8
ENSG00000068615	REEP1	10.85849309	4.09E-13	3.74813	0.1	86
ENSG00000182175	RGMA	6.949509936	1.32E-59	7.00705	0.1	60.4
ENSG00000182901	RGS7	5.631885233	0.004780135	3.62984	0.1	103.4
ENSG00000079841	RIMS1	5.422909574	0.001853635	2.75855	0.204057	99.6
ENSG00000113269	RNF130	2.407878008	3.02E-117	3.05284	0.1	61.4
ENSG00000101282	RSPO4	4.332419019	0.023824854	8.45699	3.44646	154.4
ENSG00000198626	RYR2	2.748472036	3.13E-13	2.29691	0.1	59.4
ENSG00000182568	SATB1	1.020879589	6.41E-06	3.51319	0.1	57.6
ENSG00000136155	SCEL	13.52057437	9.66E-22	5.7932	0.1	95.6
ENSG00000136531	SCN2A	3.126708503	4.70E-55	1.54419	0.1	56
ENSG00000196876	SCN8A	2.030421551	2.56E-10	4.11535	2.98366	72
ENSG00000153993	SEMA3D	6.396655115	0.000270668	3.22681	0.1	182.4
ENSG00000198879	SFMBT2	5.387894765	1.90E-11	4.89294	0.1	130.4
ENSG00000229415	SFTA3	6.034845302	0.009412113	11.4754	0.1	190.2
ENSG00000196660	SLC30A10	5.308139373	0.003013829	3.02296	0.1	56.6
ENSG00000183780	SLC35F3	6.701722804	1.54E-12	5.18045	0.1	83.2
ENSG00000163817	SLC6A20	7.805611765	3.26E-35	4.94071	0.1	55.4
ENSG00000187122	SLIT1	6.401296074	0.000384037	5.42776	2.39365	70.2
ENSG00000184347	SLIT3	4.927348749	4.75E-17	2.35098	0.1	171.4
ENSG00000064692	SNCAIP	5.741720262	2.41E-63	4.2239	0.1	90.6
ENSG00000112320	SOBP	4.793058081	0.00018846	3.04082	0.584244	76.2
ENSG00000134532	SOX5	8.742544913	2.44E-09	1.27875	0.1	70.8
ENSG00000164651	SP8	6.220375029	0.000457079	18.1607	0.1	103.8
ENSG00000174015	SPERT	7.994608517	9.59E-07	6.85764	2.68388	82.4
ENSG00000164266	SPINK1	8.014297171	1.26E-06	6.05673	0.0530902	60.6
ENSG00000097096	SYDE2	9.44619991	3.12E-19	6.12467	0.1	70.8
ENSG00000165025	SYK	10.43672667	5.53E-89	4.42804	0.1	62.2
ENSG00000164532	TBX20	10.19612376	1.78E-11	4.36934	0.0112573	53.6
ENSG00000133863	TEX15	9.859807384	2.87E-30	6.37279	0.1	55
ENSG00000095587	TLL2	1.951129361	0.002214942	4.72509	2.14627	123.2
ENSG00000137648	TMPRSS4	10.98617099	4.13E-38	4.93755	0.555597	129

ENSG00000138741	TRPC3	5.054926967	0.003296976	7.41822	0.1	120.4
ENSG00000133107	TRPC4	4.451015655	0.013380102	2.41199	0.1	62.2
ENSG00000165125	TRPV6	4.521402293	0.00791306	11.2164	8.54968	56
ENSG00000148704	VAX1	7.698407233	1.99E-12	12.673	0.1	61
ENSG00000122574	WIPF3	4.509184613	0.016800889	10.7716	0.775694	113
ENSG00000165238	WNK2	8.523753963	1.01E-15	4.18549	0.1	60.4
ENSG00000184937	WT1	10.89886142	4.68E-13	8.26546	0.1	92.2
ENSG00000152977	ZIC1	8.350915556	4.03E-22	3.54321	0.1	111.8
ENSG00000043355	ZIC2	10.72174017	7.13E-13	17.2059	0.1	65.4
ENSG00000174963	ZIC4	7.849963581	1.93E-06	9.65029	0.1	111.8
ENSG00000139800	ZIC5	9.002121673	1.51E-17	15.5894	0.1	65.4

Supplementary Table 4. Overall survival analysis of TFs

Disease	Survival.type	Gene	Num.of.samples	coef	Exp.coef.	Coxp	CoxpFDR	LRp	LRpFDR
ESCA	overall survival	FOXP2	173	-0.1245	0.8829	0.00222	0.00888	0.003959	0.01584
ESCA	overall survival	NKX6-1	182	-0.07249	0.9301	0.1887	0.3774	0.0206	0.04608
ESCA	overall survival	TBX20	88	0.09022	1.094	0.094	0.1253	0.04281	0.05708
ESCA	overall survival	FOXF1	184	0.04711	1.048	0.5687	0.6253	0.1336	0.5267
ESCA	overall survival	NR3C2	184	-0.0269	0.9735	0.5873	0.6184	0.1391	0.1472
ESCA	overall survival	HOXA10	184	0.01126	1.011	0.8912	0.9047	0.1635	0.327
ESCA	overall survival	LMX1B	178	0.02606	1.026	0.4888	0.6707	0.1639	0.3636
ESCA	overall survival	MEF2C	184	0.06159	1.064	0.5078	0.6771	0.2005	0.2673
ESCA	overall survival	MSX2	184	0.0234	1.024	0.7692	0.9545	0.2623	0.4228
ESCA	overall survival	BARX2	184	-0.06519	0.9369	0.2113	0.2113	0.2703	0.4834
ESCA	overall survival	NKX2-5	95	0.0197	1.02	0.6092	0.7318	0.3198	0.7902
ESCA	overall survival	DMRT1	184	-0.0007924	0.9992	0.9858	0.9858	0.3899	0.9314
ESCA	overall survival	PRDM6	181	-0.0476	0.9535	0.4815	0.8411	0.5374	0.8911
ESCA	overall survival	HOXB6	184	0.01059	1.011	0.8326	0.9966	0.6299	0.6299
ESCA	overall survival	NKX3-1	184	-0.03813	0.9626	0.5849	0.9107	0.7229	0.9224
ESCA	overall survival	ALX4	73	-0.03654	0.9641	0.5269	0.5269	0.7333	0.8299
ESCA	overall survival	NKX2-8	138	0.01261	1.013	0.8186	0.8186	0.7966	0.8632
ESCA	overall survival	HOXB7	184	0.05306	1.054	0.5794	0.7896	0.8124	0.8883
ESCA	overall survival	POU3F2	136	0.04125	1.042	0.2755	0.5293	0.8133	0.9128
ESCA	overall survival	DMRT2	149	0.01273	1.013	0.7205	0.7205	0.8656	0.8656
ESCA	overall survival	ZIC2	182	-0.07282	0.9298	0.07962	0.1592	0.9093	0.9231
ESCA	overall survival	ID4	184	0.08384	1.087	0.2482	0.2487	0.9302	0.9302
ESCA	overall survival	DLX6	154	0.03887	1.04	0.2863	0.4232	0.9445	0.9869
ESCA	overall survival	GRIP1	184	-0.02176	0.9785	0.7186	0.8784	0.9898	0.9898

Supplementary Table 5. Differential gene expression of TBX20 knockdown

gene	count(mean)	LFC	pvalue	padj	FDR	symbol	label
ENSG00000006125	5373.06887	0.18637934	0.00020876	0.00813306	0.01791542	AP2B1	KYSE510_up
ENSG00000006327	1225.12197	0.38464632	4.16E-06	0.00040254	0.00088889	TNFRSF12A	KYSE510_up
ENSG00000013588	995.941451	0.33254146	4.43E-05	0.002344	0.00522316	GPRC5A	KYSE510_up
ENSG00000014216	5297.67353	0.18586998	0.00068463	0.022139	0.04818297	CAPN1	KYSE510_up
ENSG00000025772	1368.84987	0.33554866	6.29E-06	0.00053435	0.00118475	TOMM34	KYSE510_up
ENSG00000027697	951.468878	0.36587889	5.88E-06	0.0005143	0.0011396	IFNGR1	KYSE510_up
ENSG00000038382	5073.16562	0.27962319	5.04E-06	0.00047169	0.00104275	TRIO	KYSE510_up
ENSG00000065361	652.371707	0.36542625	0.00020698	0.00810105	0.01784194	ERBB3	KYSE510_up
ENSG00000067057	2566.90463	0.25173933	8.32E-05	0.00387629	0.00861687	PFKP	KYSE510_up
ENSG00000071127	3185.61108	0.24178006	4.10E-05	0.00218138	0.00486004	WDR1	KYSE510_up
ENSG00000072110	3669.94259	0.22987247	0.0001165	0.00515989	0.01148487	ACTN1	KYSE510_up
ENSG00000073060	2880.13477	0.26469875	1.29E-05	0.0009312	0.00205688	SCARB1	KYSE510_up
ENSG00000073282	4190.72024	0.22270238	0.00019074	0.00758992	0.01670774	TP63	KYSE510_up
ENSG00000075142	918.425288	0.32794477	0.00020023	0.00791067	0.01741677	SRI	KYSE510_up
ENSG00000077157	329.036177	0.55945267	5.03E-05	0.00257852	0.00575006	PPP1R12B	KYSE510_up
ENSG00000100320	1410.91924	0.27126061	0.00068712	0.022139	0.04818297	RBFOX2	KYSE510_up
ENSG00000100345	18059.1284	0.2660811	8.62E-09	1.96E-06	4.48E-06	MYH9	KYSE510_up
ENSG00000100906	1870.15352	0.46345434	1.26E-10	4.07E-08	9.30E-08	NFKBIA	KYSE510_up
ENSG00000101608	3165.5071	0.19806172	0.00057198	0.01940826	0.04228944	MYL12A	KYSE510_up
ENSG00000103257	43605.8901	0.23914141	7.12E-08	1.25E-05	2.85E-05	SLC7A5	KYSE510_up
ENSG00000105953	1937.00906	0.24714042	0.00050113	0.01757083	0.03853279	OGDH	KYSE510_up
ENSG00000106211	4070.72155	0.31353173	5.23E-05	0.00266679	0.0059486	HSPB1	KYSE510_up
ENSG00000106771	918.339713	0.32333061	0.00013034	0.00571256	0.01265417	TMEM245	KYSE510_up
ENSG00000107263	1296.56668	0.31437058	0.00010271	0.00457301	0.01017722	RAPGEF1	KYSE510_up
ENSG00000109790	2034.11472	0.24905528	0.00062219	0.02054693	0.04482734	KLHL5	KYSE510_up
ENSG00000110047	668.486134	0.45589377	2.64E-05	0.0014827	0.00329906	EHD1	KYSE510_up
ENSG00000111859	237.829726	0.66937927	1.88E-05	0.00116955	0.0025948	NEDD9	KYSE510_up
ENSG00000112096	1975.95833	0.27209218	0.00010067	0.00453017	0.01007909	SOD2	KYSE510_up
ENSG00000112378	6476.30258	0.19804633	1.66E-05	0.00108178	0.00239687	PERP	KYSE510_up
ENSG00000113140	1590.20904	0.50032688	3.75E-12	1.97E-09	4.51E-09	SPARC	KYSE510_up
ENSG00000114019	904.428609	0.42536679	1.57E-06	0.00018626	0.00041383	AMOTL2	KYSE510_up
ENSG00000115009	197.537049	0.88469804	1.01E-05	NA	0.00173069	CCL20	KYSE510_up
ENSG00000116016	1027.80864	0.31180797	7.41E-05	0.00352319	0.00782717	EPAS1	KYSE510_up
ENSG00000116133	6932.13841	0.29683801	2.42E-10	7.03E-08	1.61E-07	DHCR24	KYSE510_up
ENSG00000116717	602.104497	0.40932992	0.00035753	0.01308077	0.02875612	GADD45A	KYSE510_up
ENSG00000116729	1150.05988	0.33634017	0.0002157	0.00829016	0.01827041	WLS	KYSE510_up
ENSG00000117318	664.424763	0.51191467	4.59E-08	8.40E-06	1.92E-05	ID3	KYSE510_up

ENSG00000117410	904.766745	0.3040236	0.00052643	0.01830525	0.04015666	ATP6V0B	KYSE510_up
ENSG00000117448	1290.84287	0.3003351	0.00013497	0.00585439	0.01297239	AKR1A1	KYSE510_up
ENSG00000117868	1459.51778	0.2862144	6.64E-05	0.00326923	0.00725597	ESYT2	KYSE510_up
ENSG00000118503	999.119042	0.65161266	1.59E-13	1.12E-10	2.55E-10	TNFAIP3	KYSE510_up
ENSG00000118508	414.424789	0.49640386	0.00027924	0.01035159	0.02274511	RAB32	KYSE510_up
ENSG00000120885	1967.65691	0.47866059	1.89E-13	1.23E-10	2.80E-10	CLU	KYSE510_up
ENSG00000120889	1191.33485	0.42937655	4.68E-07	6.91E-05	0.00015522	TNFRSF10B	KYSE510_up
ENSG00000127561	165.891242	0.64980664	0.00065771	NA	0.04665349	SYNGR3	KYSE510_up
ENSG00000128602	551.379864	0.48848403	2.43E-05	0.00140632	0.00312747	SMO	KYSE510_up
ENSG00000129116	1356.5111	0.28986852	7.91E-05	0.00373761	0.00830481	PALLD	KYSE510_up
ENSG00000131473	4091.19845	0.22665322	4.03E-05	0.00216065	0.00481311	ACLY	KYSE510_up
ENSG00000131981	1096.11573	0.32446845	8.60E-05	0.00395532	0.00879512	LGALS3	KYSE510_up
ENSG00000134871	1943.41779	0.3214507	0.00034184	0.01256148	0.02761	COL4A2	KYSE510_up
ENSG00000135046	3523.92444	0.19750132	0.00062788	0.02055894	0.04486924	ANXA1	KYSE510_up
ENSG00000135480	15024.2317	0.28894714	1.89E-11	7.58E-09	1.73E-08	KRT7	KYSE510_up
ENSG00000135821	2563.65461	0.27749141	0.00037992	0.01372104	0.0301783	GLUL	KYSE510_up
ENSG00000136205	2506.43229	0.32484272	7.31E-07	0.00010247	0.00023025	TNS3	KYSE510_up
ENSG00000137673	150.994864	0.70959993	0.00013015	NA	0.01265417	MMP7	KYSE510_up
ENSG00000137801	2226.78117	0.30856837	1.09E-05	0.00082694	0.00182334	THBS1	KYSE510_up
ENSG00000140416	621.543185	0.45687675	7.81E-06	0.00064588	0.00143328	TPM1	KYSE510_up
ENSG00000142910	1519.47758	0.45328985	3.71E-10	9.76E-08	2.23E-07	TINAGL1	KYSE510_up
ENSG00000143061	1265.00271	0.33075339	4.53E-05	0.00238233	0.0053094	IGSF3	KYSE510_up
ENSG00000143878	422.024926	0.5404463	1.79E-05	0.00113041	0.00250688	RHOB	KYSE510_up
ENSG00000145860	1700.87738	0.27125786	4.92E-05	0.00255796	0.00570254	RNF145	KYSE510_up
ENSG00000145901	1660.65773	0.31334213	2.63E-05	0.0014827	0.00329906	TNIP1	KYSE510_up
ENSG00000146411	159.267208	1.03957218	3.73E-06	NA	0.00081439	SLC2A12	KYSE510_up
ENSG00000148175	1475.62712	0.28179527	5.85E-05	0.00292872	0.00653471	STOM	KYSE510_up
ENSG00000151726	840.793016	0.3603517	4.92E-05	0.00255796	0.00570254	ACSL1	KYSE510_up
ENSG00000152104	1324.92056	0.27450149	0.00056448	0.01934776	0.04214181	PTPN14	KYSE510_up
ENSG00000152492	705.628515	0.40257774	0.00014057	0.00602115	0.01328717	CCDC50	KYSE510_up
ENSG00000153395	2459.23969	0.31569538	2.90E-06	0.00030044	0.00066999	LPCAT1	KYSE510_up
ENSG00000161091	977.778463	0.36402799	1.25E-05	0.00091452	0.00201888	MFSD12	KYSE510_up
ENSG00000161921	703.339326	0.3630493	8.77E-05	0.00400961	0.00891713	CXCL16	KYSE510_up
ENSG00000162337	1053.9434	0.3008191	0.00047927	0.01694556	0.03714909	LRP5	KYSE510_up
ENSG00000162434	1460.71682	0.31850127	2.52E-05	0.00143545	0.0031928	JAK1	KYSE510_up
ENSG00000162493	376.984533	1.04865369	1.85E-05	0.00116288	0.00257944	PDPN	KYSE510_up
ENSG00000163347	745.091908	0.47413523	1.53E-05	0.00102709	0.00227406	CLDN1	KYSE510_up
ENSG00000163430	628.176418	0.44837001	3.49E-05	0.0018827	0.00419327	FSTL1	KYSE510_up
ENSG00000163739	974.393259	0.55168989	1.26E-08	2.64E-06	6.04E-06	CXCL1	KYSE510_up
ENSG00000163874	629.902992	0.40316541	7.37E-05	0.00352319	0.00782717	ZC3H12A	KYSE510_up

ENSG00000164236	829.033514	0.51021694	2.13E-08	4.36E-06	9.97E-06	ANKRD33B	KYSE510_up
ENSG00000164733	5825.16471	0.23626042	3.15E-06	0.0003158	0.00070463	CTSB	KYSE510_up
ENSG00000165802	1379.82085	0.32564769	1.50E-05	0.00102459	0.00226741	NSMF	KYSE510_up
ENSG00000166750	692.73936	0.4648988	5.33E-07	7.60E-05	0.00017064	SLFN5	KYSE510_up
ENSG00000166949	926.984504	0.35989789	6.03E-05	0.00300022	0.00665668	SMAD3	KYSE510_up
ENSG00000167601	380.680686	0.53778822	1.59E-05	0.00104846	0.0023225	AXL	KYSE510_up
ENSG00000167996	30301.6362	0.23077418	1.12E-08	2.41E-06	5.51E-06	FTH1	KYSE510_up
ENSG00000168003	11308.8983	0.21285123	7.83E-06	0.00064588	0.00143328	SLC3A2	KYSE510_up
ENSG00000168610	2844.43926	0.28939863	1.22E-06	0.00015136	0.00034074	STAT3	KYSE510_up
ENSG00000169242	390.320052	0.49968556	3.19E-05	0.00174336	0.00388167	EFNA1	KYSE510_up
ENSG00000169429	931.337151	0.72459678	6.16E-13	3.46E-10	7.90E-10	CXCL8	KYSE510_up
ENSG00000169908	1078.53718	0.41568218	3.96E-06	0.00038789	0.00085623	TM4SF1	KYSE510_up
ENSG00000172893	15661.2511	0.17291488	0.00032037	0.01182415	0.02598505	DHCR7	KYSE510_up
ENSG00000173391	840.69887	0.54351519	2.19E-10	6.59E-08	1.51E-07	OLR1	KYSE510_up
ENSG00000177469	2757.19811	0.2223011	0.00025117	0.0093937	0.02072194	CAVIN1	KYSE510_up
ENSG00000177606	712.54673	0.64810646	2.87E-11	1.10E-08	2.51E-08	JUN	KYSE510_up
ENSG00000184009	27333.1916	0.17194071	0.00060075	0.01998132	0.04357785	ACTG1	KYSE510_up
ENSG00000184254	6320.47484	0.2654779	3.54E-08	6.78E-06	1.55E-05	ALDH1A3	KYSE510_up
ENSG00000184292	4786.29934	0.40321027	5.76E-13	3.46E-10	7.90E-10	TACSTD2	KYSE510_up
ENSG00000185585	374.529235	0.63717186	1.50E-05	0.00102459	0.00226741	OLFML2A	KYSE510_up
ENSG00000187244	639.480866	0.37508718	0.00015511	0.00649399	0.01433545	BCAM	KYSE510_up
ENSG00000188064	2436.75408	0.28586937	6.12E-05	0.00302778	0.00671896	WNT7B	KYSE510_up
ENSG00000188229	6608.16718	0.1991824	0.00017524	0.00712403	0.01574161	TUBB4B	KYSE510_up
ENSG00000197386	1624.71875	0.28800769	8.59E-05	0.00395532	0.00879512	HTT	KYSE510_up
ENSG00000197747	2783.73609	0.20380341	0.00045168	0.0161054	0.03529519	S100A10	KYSE510_up
ENSG00000198719	471.212772	0.58946589	2.41E-05	0.00140632	0.00312747	DLL1	KYSE510_up
ENSG00000198911	2930.52687	0.23349787	6.78E-05	0.00331546	0.0073598	SREBF2	KYSE510_up
ENSG00000204388	1507.71215	0.29848966	7.29E-05	0.00350717	0.00778914	HSPA1B	KYSE510_up
ENSG00000204389	1537.68743	0.37141414	1.29E-05	0.0009312	0.00205688	HSPA1A	KYSE510_up
ENSG00000204525	2122.68482	0.24745496	7.29E-05	0.00350717	0.00778914	HLA-C	KYSE510_up
ENSG00000204580	5877.09751	0.34311081	6.09E-12	3.02E-09	6.89E-09	DDR1	KYSE510_up
ENSG00000205336	1839.56447	0.30214033	1.96E-05	0.00120298	0.0026701	ADGRG1	KYSE510_up
ENSG00000206503	4205.95311	0.25016846	2.85E-05	0.00157623	0.00350836	HLA-A	KYSE510_up
ENSG00000234745	1141.61737	0.38003053	2.06E-06	0.00022263	0.00049585	HLA-B	KYSE510_up
ENSG00000272398	2268.80141	0.26218115	1.94E-05	0.00120135	0.00266593	CD24	KYSE510_up
ENSG00000004700	882.664293	-0.3402339	0.00037235	0.01355363	0.02980531	RECQL	KYSE510_dc
ENSG00000012048	1214.12074	-0.3383292	2.24E-05	0.0013444	0.0029858	BRCA1	KYSE510_dc
ENSG00000012983	769.22876	-0.4114495	9.42E-06	0.00074766	0.00166093	MAP4K5	KYSE510_dc
ENSG00000035403	2111.46263	-0.7734844	1.67E-32	4.68E-29	1.07E-28	VCL	KYSE510_dc
ENSG00000035928	1737.88914	-0.2599497	0.00070195	0.02237468	0.04871335	RFC1	KYSE510_dc

ENSG00000044574	10328.7577	-0.1689604	0.00062263	0.02054693	0.04482734	HSPA5	KYSE510_dc
ENSG00000047634	169.021127	-1.1351139	4.46E-07	NA	0.00015308	SCML1	KYSE510_dc
ENSG00000051341	653.68091	-0.3557403	0.00058064	0.01962289	0.04276504	POLQ	KYSE510_dc
ENSG00000053770	616.650379	-0.3587975	0.0005656	0.01934776	0.04214181	AP5M1	KYSE510_dc
ENSG00000057149	270.219947	-0.7510698	1.00E-07	1.69E-05	3.85E-05	SERPINB3	KYSE510_dc
ENSG00000060982	1383.68377	-0.3153823	0.00016455	0.0068213	0.01506299	BCAT1	KYSE510_dc
ENSG00000065308	713.586283	-0.4634501	1.34E-05	0.00095449	0.00210893	TRAM2	KYSE510_dc
ENSG00000065615	414.269819	-0.6155372	1.65E-06	0.00019073	0.00042408	CYB5R4	KYSE510_dc
ENSG00000065618	4132.8112	-0.441013	0.00037367	0.01355363	0.02980531	COL17A1	KYSE510_dc
ENSG00000066279	1710.58446	-0.3267138	9.30E-05	0.00420769	0.00936031	ASPM	KYSE510_dc
ENSG00000068796	631.939629	-0.4595725	5.89E-06	0.0005143	0.0011396	KIF2A	KYSE510_dc
ENSG00000071537	855.65305	-0.4181263	8.00E-06	0.00065333	0.00145022	SEL1L	KYSE510_dc
ENSG00000072803	644.889643	-0.4409556	2.96E-05	0.00162586	0.00361945	FBXW11	KYSE510_dc
ENSG00000074800	31856.7592	-0.2331401	1.19E-06	0.00014923	0.00033589	ENO1	KYSE510_dc
ENSG00000078177	274.29189	-0.6081571	0.00020344	0.00799968	0.01761572	N4BP2	KYSE510_dc
ENSG00000081154	1400.85337	-0.3096554	2.02E-05	0.00122928	0.00272902	PCNP	KYSE510_dc
ENSG00000085719	1102.51625	-0.3388673	2.67E-05	0.00148933	0.00331439	CPNE3	KYSE510_dc
ENSG00000087053	1150.19549	-0.3489476	1.12E-05	0.00084413	0.00186181	MTMR2	KYSE510_dc
ENSG00000089159	1895.74286	-0.289784	8.13E-06	0.00065817	0.00146136	PXN	KYSE510_dc
ENSG00000091039	866.478465	-0.4455812	1.03E-05	0.00079752	0.00175734	OSBPL8	KYSE510_dc
ENSG00000092201	4077.38149	-0.253284	1.96E-06	0.00021379	0.00047602	SUPT16H	KYSE510_dc
ENSG00000096433	2504.03124	-0.9067026	5.11E-44	4.30E-40	9.83E-40	ITPR3	KYSE510_dc
ENSG00000100504	2412.79484	-0.678001	1.29E-30	2.71E-27	6.18E-27	PYGL	KYSE510_dc
ENSG00000100580	583.361645	-0.6174682	7.51E-09	1.75E-06	4.01E-06	TMED8	KYSE510_dc
ENSG00000100644	4445.01805	-0.2307657	0.0001712	0.00704239	0.01555626	HIF1A	KYSE510_dc
ENSG00000100941	2214.1775	-0.2606662	0.00017156	0.00704239	0.01555626	PNN	KYSE510_dc
ENSG00000101412	321.151386	-0.5988687	5.55E-06	0.00050189	0.00111068	E2F1	KYSE510_dc
ENSG00000104738	4845.92297	-0.2316764	1.72E-06	0.00019298	0.0004294	MCM4	KYSE510_dc
ENSG00000105202	2887.56123	-0.3689268	1.07E-09	2.74E-07	6.26E-07	FBL	KYSE510_dc
ENSG00000105698	1167.3968	-0.5367555	2.25E-08	4.52E-06	1.03E-05	USF2	KYSE510_dc
ENSG00000106683	617.552579	-0.467647	3.59E-06	0.00035512	0.00079258	LIMK1	KYSE510_dc
ENSG00000107731	361.244958	-0.7905252	1.28E-09	3.18E-07	7.26E-07	UNC5B	KYSE510_dc
ENSG00000108055	1294.35213	-0.3810651	7.34E-06	0.00061771	0.00136999	SMC3	KYSE510_dc
ENSG00000108602	14648.1836	-0.2541657	2.92E-06	0.00030044	0.00066999	ALDH3A1	KYSE510_dc
ENSG00000108946	1208.19283	-0.5152094	1.23E-10	4.07E-08	9.30E-08	PRKAR1A	KYSE510_dc
ENSG00000109814	2631.08725	-0.2358547	0.00013718	0.00591992	0.01311963	UGDH	KYSE510_dc
ENSG00000110092	7816.62596	-0.2312366	1.38E-06	0.00016814	0.00037328	CCND1	KYSE510_dc
ENSG00000110400	3450.94403	-0.335636	1.13E-07	1.83E-05	4.19E-05	NECTIN1	KYSE510_dc
ENSG00000110435	906.000361	-0.34261	0.00024477	0.00927813	0.02045751	PDHX	KYSE510_dc
ENSG00000111331	977.348307	-0.3764087	3.25E-05	0.00176299	0.003926	OAS3	KYSE510_dc

ENSG00000113387	1759.6829	-0.3547641	8.14E-07	0.00010995	0.00024725	SUB1	KYSE510_dc
ENSG00000113575	1140.53634	-0.3331299	1.55E-05	0.00102926	0.00227943	PPP2CA	KYSE510_dc
ENSG00000113810	2689.60594	-0.3026099	8.34E-05	0.00387629	0.00861687	SMC4	KYSE510_dc
ENSG00000114346	1337.95446	-0.327815	0.00012912	0.00568874	0.01265417	ECT2	KYSE510_dc
ENSG00000116539	1318.83262	-0.3801165	4.72E-07	6.91E-05	0.00015522	ASH1L	KYSE510_dc
ENSG00000117500	643.693259	-0.4299027	1.76E-05	0.0011214	0.00248636	TMED5	KYSE510_dc
ENSG00000117724	3173.6753	-0.2627752	0.0005984	0.01998132	0.04357201	CENPF	KYSE510_dc
ENSG00000117899	415.629755	-1.1284623	2.43E-17	2.93E-14	6.68E-14	MESD	KYSE510_dc
ENSG00000117906	958.836089	-0.4686886	5.93E-06	0.0005143	0.0011396	RCN2	KYSE510_dc
ENSG00000119402	1052.5579	-0.3142876	0.00014143	0.00602115	0.01328717	FBXW2	KYSE510_dc
ENSG00000119446	378.696333	-0.4986915	0.00014239	0.00602115	0.01328717	RBM18	KYSE510_dc
ENSG00000120137	1078.04405	-0.3987584	5.04E-06	0.00047169	0.00104275	PANK3	KYSE510_dc
ENSG00000121579	5153.7055	-0.448955	1.28E-16	1.35E-13	3.08E-13	NAA50	KYSE510_dc
ENSG00000122482	523.550769	-0.5520072	2.44E-06	0.00025946	0.00057807	ZNF644	KYSE510_dc
ENSG00000123178	174.649463	-0.7997701	0.00013839	NA	0.01317001	SPRYD7	KYSE510_dc
ENSG00000123384	659.082751	-0.465871	5.26E-05	0.00266679	0.0059486	LRP1	KYSE510_dc
ENSG00000124767	2261.38207	-0.3234837	1.71E-06	0.00019298	0.0004294	GLO1	KYSE510_dc
ENSG00000125743	1365.88285	-0.4088392	8.23E-07	0.00010995	0.00024725	SNRPD2	KYSE510_dc
ENSG00000127914	941.28117	-0.3782956	0.00017982	0.00727499	0.01607772	AKAP9	KYSE510_dc
ENSG00000129083	1433.98621	-0.2941352	0.00026404	0.00983157	0.02159884	COPB1	KYSE510_dc
ENSG00000130821	1681.61566	-0.3085557	2.42E-05	0.00140632	0.00312747	SLC6A8	KYSE510_dc
ENSG00000131747	5097.80751	-0.3189109	3.14E-08	6.15E-06	1.41E-05	TOP2A	KYSE510_dc
ENSG00000132485	1131.83124	-0.303918	0.00048903	0.01721822	0.03775321	ZRANB2	KYSE510_dc
ENSG00000134333	14140.8378	-0.2099399	6.06E-06	0.00052011	0.00115284	LDHA	KYSE510_dc
ENSG00000134371	621.99963	-0.3631203	0.00057129	0.01940826	0.04228944	CDC73	KYSE510_dc
ENSG00000134709	469.88635	-0.4155675	0.00065305	0.02130008	0.04649477	HOOK1	KYSE510_dc
ENSG00000135090	391.870216	-0.5281831	1.06E-05	0.00080821	0.00178147	TAOK3	KYSE510_dc
ENSG00000136824	1226.50101	-0.3663269	9.29E-05	0.00420769	0.00936031	SMC2	KYSE510_dc
ENSG00000137288	585.637387	-0.4048914	0.00024402	0.00927813	0.02045751	UQCC2	KYSE510_dc
ENSG00000137807	1201.72481	-0.2775321	0.0002483	0.00932786	0.02057354	KIF23	KYSE510_dc
ENSG00000137955	722.443074	-0.3722943	7.96E-05	0.00374189	0.0083156	RABGGTB	KYSE510_dc
ENSG00000137975	129.019302	-0.8057522	5.99E-05	NA	0.00665356	CLCA2	KYSE510_dc
ENSG00000138138	610.099495	-0.7399774	6.97E-12	3.26E-09	7.45E-09	ATAD1	KYSE510_dc
ENSG00000138398	850.017561	-0.4572502	3.09E-06	0.00031311	0.00069843	PPIG	KYSE510_dc
ENSG00000138448	1541.88508	-0.3293209	2.44E-05	0.00140632	0.00312747	ITGAV	KYSE510_dc
ENSG00000140465	867.268698	-0.546024	0.00018469	0.00743607	0.01636051	CYP1A1	KYSE510_dc
ENSG00000142949	3806.98948	-0.2441441	1.74E-05	0.00112035	0.00248346	PTPRF	KYSE510_dc
ENSG00000143314	719.355186	-0.4635327	4.87E-06	0.00046585	0.0010291	MRPL24	KYSE510_dc
ENSG00000143437	477.971159	-0.4093983	0.00056479	0.01934776	0.04214181	ARNT	KYSE510_dc
ENSG00000143801	134.845166	-0.7785566	0.00018181	NA	0.01617987	PSEN2	KYSE510_dc

ENSG00000144354	1098.14307	-0.3247542	0.00010257	0.00457301	0.01017722	CDCA7	KYSE510_dc
ENSG00000144579	432.980265	-0.5527195	5.14E-06	0.00047434	0.00104935	CTDSP1	KYSE510_dc
ENSG00000145604	1118.12484	-0.2746597	0.00069311	0.02217696	0.04827427	SKP2	KYSE510_dc
ENSG00000145907	3518.5614	-0.2025442	0.0001876	0.00751734	0.01654223	G3BP1	KYSE510_dc
ENSG00000146373	475.37919	-0.5310524	2.12E-05	0.00128382	0.0028507	RNF217	KYSE510_dc
ENSG00000147403	7675.01131	-0.2267067	2.46E-05	0.00140632	0.00312747	RPL10	KYSE510_dc
ENSG00000147604	9819.7219	-0.2208741	1.54E-05	0.00102709	0.00227406	RPL7	KYSE510_dc
ENSG00000147689	391.378361	-0.5238997	1.22E-05	0.0008977	0.00198116	FAM83A	KYSE510_dc
ENSG00000150093	2256.63073	-0.3621859	1.31E-07	2.08E-05	4.76E-05	ITGB1	KYSE510_dc
ENSG00000152291	2461.29337	-0.2922686	1.16E-06	0.0001478	0.0003326	TGOLN2	KYSE510_dc
ENSG00000152332	1773.93356	-0.6446204	1.80E-18	3.03E-15	6.92E-15	UHMK1	KYSE510_dc
ENSG00000152558	8657.46691	-0.2377144	5.32E-05	0.00268131	0.00598183	TMEM123	KYSE510_dc
ENSG00000152601	2044.29266	-0.4030184	3.25E-07	4.97E-05	0.00011347	MBNL1	KYSE510_dc
ENSG00000153914	980.268337	-0.2989417	0.00068425	0.022139	0.04818297	SREK1	KYSE510_dc
ENSG00000153922	880.947357	-0.5172881	5.03E-08	9.01E-06	2.06E-05	CHD1	KYSE510_dc
ENSG00000156504	587.738976	-0.4496774	2.29E-05	0.00136761	0.00303795	FAM122B	KYSE510_dc
ENSG00000156535	3114.25171	-0.2199227	0.00014175	0.00602115	0.01328717	CD109	KYSE510_dc
ENSG00000156976	2720.74739	-0.2163181	0.00058948	0.01978809	0.04314087	EIF4A2	KYSE510_dc
ENSG00000158417	2283.86597	-0.3261994	0.00013343	0.00581755	0.01288876	EIF5B	KYSE510_dc
ENSG00000160767	752.948249	-0.5133272	1.08E-07	1.78E-05	4.06E-05	FAM189B	KYSE510_dc
ENSG00000163029	758.723905	-0.4196952	1.16E-05	0.00086554	0.00190963	SMC6	KYSE510_dc
ENSG00000163428	1394.21107	-1.1326031	7.39E-42	3.11E-38	7.10E-38	LRRC58	KYSE510_dc
ENSG00000163605	480.775382	-0.4344791	0.00042099	0.0151393	0.03316644	PPP4R2	KYSE510_dc
ENSG00000163694	720.577124	-0.5216199	1.06E-08	2.35E-06	5.36E-06	RBM47	KYSE510_dc
ENSG00000164221	115.349822	-0.8659957	0.0002625	NA	0.021564	CCDC112	KYSE510_dc
ENSG00000164754	7964.53394	-0.2127087	8.41E-06	0.00067372	0.00149628	RAD21	KYSE510_dc
ENSG00000164985	1883.00281	-0.2968686	0.00016076	0.00669692	0.01478588	PSIP1	KYSE510_dc
ENSG00000165272	672.537092	-0.542363	9.86E-08	1.69E-05	3.85E-05	AQP3	KYSE510_dc
ENSG00000165525	627.475578	-0.3757301	0.00014481	0.00609298	0.01344797	NEMF	KYSE510_dc
ENSG00000165983	391.130733	-1.0026124	9.40E-11	3.29E-08	7.53E-08	PTER	KYSE510_dc
ENSG00000166598	7201.66822	-0.2774445	2.67E-09	6.42E-07	1.47E-06	HSP90B1	KYSE510_dc
ENSG00000167005	2348.90291	-0.261105	4.97E-05	0.00256629	0.00572195	NUDT21	KYSE510_dc
ENSG00000167797	361.303633	-0.6767949	1.82E-06	0.00020149	0.00044847	CDK2AP2	KYSE510_dc
ENSG00000167977	856.104069	-0.3943504	1.72E-05	0.00111524	0.00247157	KCTD5	KYSE510_dc
ENSG00000168209	6664.92425	-0.3173289	4.76E-07	6.91E-05	0.00015522	DDIT4	KYSE510_dc
ENSG00000168439	4192.11404	-0.3553315	9.81E-12	4.13E-09	9.43E-09	STIP1	KYSE510_dc
ENSG00000168453	346.443883	-0.4751981	0.000212	0.00822117	0.01811247	HR	KYSE510_dc
ENSG00000168906	3787.87317	-0.2575095	7.79E-07	0.00010753	0.00024168	MAT2A	KYSE510_dc
ENSG00000169045	5348.48847	-0.1822192	0.00024751	0.00932786	0.02057354	HNRNPH1	KYSE510_dc
ENSG00000169860	311.05202	-0.7068202	7.16E-05	0.00348285	0.00773263	P2RY1	KYSE510_dc

ENSG00000170017	3553.61391	-0.267187	9.56E-06	0.00075176	0.00167046	ALCAM	KYSE510_dc
ENSG00000170027	4914.78809	-0.189572	0.00021575	0.00829016	0.01827041	YWHAG	KYSE510_dc
ENSG00000171208	960.112837	-0.2958384	0.00047811	0.01694556	0.03714909	NETO2	KYSE510_dc
ENSG00000171346	2984.95804	-0.4272852	9.59E-12	4.13E-09	9.43E-09	KRT15	KYSE510_dc
ENSG00000172336	373.273186	-0.8366129	3.34E-10	9.06E-08	2.07E-07	POP7	KYSE510_dc
ENSG00000174437	6166.06809	-0.1760432	0.00019121	0.00758992	0.01670774	ATP2A2	KYSE510_dc
ENSG00000174748	7117.50371	-0.4435975	8.46E-15	7.12E-12	1.63E-11	RPL15	KYSE510_dc
ENSG00000174903	1922.822	-0.4431346	6.13E-11	2.24E-08	5.12E-08	RAB1B	KYSE510_dc
ENSG00000175595	216.506366	-0.5995108	0.00059023	0.01978809	0.04314087	ERCC4	KYSE510_dc
ENSG00000176153	1576.56337	-0.4210308	2.62E-10	7.35E-08	1.68E-07	GPX2	KYSE510_dc
ENSG00000177189	877.146876	-0.6878817	1.40E-14	1.07E-11	2.44E-11	RPS6KA3	KYSE510_dc
ENSG00000182551	698.016874	-0.3292842	0.0006893	0.022139	0.04818297	ADI1	KYSE510_dc
ENSG00000182934	947.210809	-0.3209241	0.0005406	0.01872067	0.04091298	SRPRA	KYSE510_dc
ENSG00000185515	392.333057	-0.4503053	0.00021986	0.00840968	0.01853677	BRCC3	KYSE510_dc
ENSG00000186081	27728.7001	-0.2134011	1.48E-05	0.00102459	0.00226741	KRT5	KYSE510_dc
ENSG00000186350	1728.78406	-0.2355042	0.00062696	0.02055894	0.04486924	RXRA	KYSE510_dc
ENSG00000187837	233.786133	-0.7130314	1.42E-05	0.00100372	0.00221831	HIST1H1C	KYSE510_dc
ENSG00000188994	556.522852	-0.5712083	1.13E-06	0.00014691	0.00033052	ZNF292	KYSE510_dc
ENSG00000196396	763.866417	-0.5218025	4.42E-08	8.26E-06	1.89E-05	PTPN1	KYSE510_dc
ENSG00000198363	2938.57944	-0.5217989	1.55E-17	2.18E-14	4.97E-14	ASPH	KYSE510_dc
ENSG00000198478	146.98145	-0.6908939	0.00053772	NA	0.04085627	SH3BGRL2	KYSE510_dc
ENSG00000198887	744.284354	-0.4887681	1.51E-05	0.00102459	0.00226741	SMC5	KYSE510_dc
ENSG00000204516	139.958369	-0.6723964	0.00055904	NA	0.04214181	MICB	KYSE510_dc
ENSG00000205420	9374.91257	-0.7564428	3.97E-15	3.71E-12	8.48E-12	KRT6A	KYSE510_dc
ENSG00000229117	3891.82294	-0.3522463	2.60E-07	4.05E-05	9.24E-05	RPL41	KYSE510_dc
ENSG00000249992	24.7132379	-2.1219377	0.00038357	NA	0.03034282	TMEM158	KYSE510_dc
ENSG00000265808	329.169502	-0.6877547	1.63E-06	0.00019073	0.00042397	SEC22B	KYSE510_dc
ENSG00000275700	1202.75856	-0.3843558	1.49E-06	0.00017859	0.00039663	AATF	KYSE510_dc
ENSG00000276293	680.932547	-0.5062333	9.51E-07	0.00012504	0.00028124	PIP4K2B	KYSE510_dc

Supplementary Table 6. Functional enriched terms of Metascape analysis

GO	Description	LogP	Enrichment
GO:0007059	chromosome segregation	-7.276512447	6.98489305
GO:0044770	cell cycle phase transition	-7.029103504	4.84491561
GO:0044772	mitotic cell cycle phase transition	-6.721891519	4.91245225
GO:0098813	nuclear chromosome segregation	-6.412927072	7.2807971
GO:0006310	DNA recombination	-6.056146856	6.69833333
GO:0000724	double-strand break repair via homologous recombination	-6.03423159	10.6675514
GO:0000725	recombinational repair	-5.986554262	10.5140615
GO:0051301	cell division	-5.935191008	4.56704545
GO:0000819	sister chromatid segregation	-5.737442042	8.13928893
GO:0006281	DNA repair	-5.50900389	4.51066218
GO:0044839	cell cycle G2/M phase transition	-5.506500929	6.61890646
GO:0000280	nuclear division	-5.386797026	5.17024014
GO:0045047	protein targeting to ER	-5.341579817	10.6564394
GO:0030261	chromosome condensation	-5.278463856	19.8567194
GO:0072599	establishment of protein localization to endoplasmic reticulum	-5.247275566	10.3126833
ko04110	Cell cycle	-5.247275566	10.3126833
GO:0051348	negative regulation of transferase activity	-5.199773201	6.10975981
ko05222	Small cell lung cancer	-5.156452432	13.0487013
GO:0010564	regulation of cell cycle process	-5.132856727	3.70457426
hsa04110	Cell cycle	-5.068432893	9.68767218
GO:0070482	response to oxygen levels	-5.018684811	5.21948052
GO:0048285	organelle fission	-4.918299829	4.644453
GO:0071103	DNA conformation change	-4.907966633	5.65578384
GO:0000086	G2/M transition of mitotic cell cycle	-4.876423969	6.37262156
GO:1901987	regulation of cell cycle phase transition	-4.863868959	4.58615443
GO:0007346	regulation of mitotic cell cycle	-4.801148131	3.90464955
GO:0006302	double-strand break repair	-4.758398455	6.1578141
GO:1901988	negative regulation of cell cycle phase transition	-4.745572758	6.13483718
hsa05222	Small cell lung cancer	-4.745348675	11.0716253
M281	PID FAK PATHWAY	-4.740760012	15.48151
GO:0007584	response to nutrient	-4.709177332	7.06016689
GO:0070972	protein localization to endoplasmic reticulum	-4.669731287	8.41297847
GO:0006090	pyruvate metabolic process	-4.651369928	8.35799168
GO:0001666	response to hypoxia	-4.630231342	5.24947753

GO:0006614	SRP-dependent cotranslational protein targeting to membrane	-4.599825493	10.438961
GO:0140014	mitotic nuclear division	-4.57171163	5.83027079
GO:0032508	DNA duplex unwinding	-4.553356922	10.2438403
M279	PID RB 1PATHWAY	-4.534773171	14.0524476
GO:1902749	regulation of cell cycle G2/M phase transition	-4.534038339	6.67330843
GO:0010948	negative regulation of cell cycle process	-4.515523282	5.08863003
GO:0036293	response to decreased oxygen levels	-4.505306434	5.07449495
GO:1901990	regulation of mitotic cell cycle phase transition	-4.493213878	4.57744875
GO:0006613	cotranslational protein targeting to membrane	-4.485433189	9.96446281
GO:0000070	mitotic sister chromatid segregation	-4.407727709	7.65732172
GO:0032392	DNA geometric change	-4.398007529	9.61483254
GO:0090068	positive regulation of cell cycle process	-4.329218754	5.42619262
hsa04066	HIF-1 signaling pathway	-4.293417153	9.21084798
ko04114	Oocyte meiosis	-4.193630462	8.83944282
GO:0000722	telomere maintenance via recombination	-4.156530378	36.5363636
GO:0072657	protein localization to membrane	-4.14573549	3.60921525
GO:0051188	cofactor biosynthetic process	-4.136833776	5.12192014
GO:0008380	RNA splicing	-4.128887882	4.16908714
GO:1901991	negative regulation of mitotic cell cycle phase transition	-4.128848595	5.84581818
GO:0051321	meiotic cell cycle	-4.116773879	5.82252807
M186	PID PDGFRB PATHWAY	-4.098259346	8.49682875
GO:0080135	regulation of cellular response to stress	-4.096112868	3.36076932
hsa04114	Oocyte meiosis	-4.0796841	8.43146853
GO:0046683	response to organophosphorus	-4.06126909	8.36710618
GO:0009108	coenzyme biosynthetic process	-4.045499531	5.68659356
GO:0045930	negative regulation of mitotic cell cycle	-4.045456499	4.9822314
GO:0071417	cellular response to organonitrogen compound	-4.017033767	3.74731935
GO:0006413	translational initiation	-4.011633762	6.62576543
hsa00010	Glycolysis / Gluconeogenesis	-3.998433892	10.8739177
ko00270	Cysteine and methionine metabolism	-3.966460458	16.2383838
M129	PID PLK1 PATHWAY	-3.928796955	15.8853755
M239	PID A6B1 A6B4 INTEGRIN PATHWAY	-3.928796955	15.8853755
ko04151	PI3K-Akt signaling pathway	-3.928221648	4.80741627
GO:0006890	retrograde vesicle-mediated transport, Golgi to ER	-3.926103312	10.4989551

GO:0010389	regulation of G2/M transition of mitotic cell cycle	-3.875406009	6.29937304
GO:0045132	meiotic chromosome segregation	-3.856479762	10.1489899
GO:0000082	G1/S transition of mitotic cell cycle	-3.842992874	5.31438017
GO:0010038	response to metal ion	-3.834300297	4.67084194
GO:0044088	regulation of vacuole organization	-3.820928787	14.9128015
GO:0014074	response to purine-containing compound	-3.818987782	7.55924765
GO:0006260	DNA replication	-3.810781878	5.25703074
GO:0051592	response to calcium ion	-3.802706984	7.50747198
GO:0090150	establishment of protein localization to membrane	-3.797644567	4.61836057
GO:0000075	cell cycle checkpoint	-3.771931889	6.06053425
GO:0006612	protein targeting to membrane	-3.771931889	6.06053425
GO:0045786	negative regulation of cell cycle	-3.737505125	3.49630274
GO:0048010	vascular endothelial growth factor receptor signaling pathway	-3.724644251	9.51467803
GO:2001251	negative regulation of chromosome organization	-3.723165279	7.25888019
GO:0006984	ER-nucleus signaling pathway	-3.687776115	13.787307
hsa04910	Insulin signaling pathway	-3.661667533	7.07155425
GO:0051383	kinetochore organization	-3.639240091	24.911157
GO:0007169	transmembrane receptor protein tyrosine kinase signaling pathway	-3.637734817	3.20494418
GO:0031570	DNA integrity checkpoint	-3.616725016	6.93728423
GO:2001234	negative regulation of apoptotic signaling pathway	-3.613225254	5.70880682
GO:0044843	cell cycle G1/S phase transition	-3.606215053	4.90420988
GO:0006303	double-strand break repair via nonhomologous end joining	-3.601672448	8.95499109
hsa00270	Cysteine and methionine metabolism	-3.59490236	13.0487013
GO:1901699	cellular response to nitrogen compound	-3.594724281	3.37258741
hsa04151	PI3K-Akt signaling pathway	-3.587908659	4.32667464
GO:0007093	mitotic cell cycle checkpoint	-3.572751522	6.80801807
GO:0046390	ribose phosphate biosynthetic process	-3.567294382	4.83925346
hsa00520	Amino sugar and nucleotide sugar metabolism	-3.565145183	12.8197767
GO:0046394	carboxylic acid biosynthetic process	-3.541535491	3.8703775
GO:0016053	organic acid biosynthetic process	-3.534268056	3.86219489
GO:0006312	mitotic recombination	-3.524037576	22.8352273
GO:2001233	regulation of apoptotic signaling pathway	-3.489136068	4.19422542
GO:0042326	negative regulation of phosphorylation	-3.469739443	3.79007922

GO:0045787	positive regulation of cell cycle	-3.465034963	4.16237054
hsa05160	Hepatitis C	-3.459955713	6.48574502
M58	PID AR PATHWAY	-3.4515677	11.9791356
GO:0000726	non-recombinational repair	-3.449734279	8.30371901
GO:0007062	sister chromatid cohesion	-3.424444331	11.7859238
GO:0051983	regulation of chromosome segregation	-3.413683698	8.15543831
GO:0140013	meiotic nuclear division	-3.37933676	6.26337662
GO:0006096	glycolytic process	-3.378350582	8.01236045
GO:0007064	mitotic sister chromatid cohesion	-3.369349874	20.2979798
GO:0006757	ATP generation from ADP	-3.360945068	7.94268775
GO:0006470	protein dephosphorylation	-3.346293647	4.4829894
GO:0090407	organophosphate biosynthetic process	-3.345217978	3.16332153
GO:0010039	response to iron ion	-3.321873537	19.5730519
GO:0010575	positive regulation of vascular endothelial growth factor production	-3.321873537	19.5730519
M255	PID HIF1 TFPATHWAY	-3.32054963	11.0716253
GO:0019083	viral transcription	-3.314569437	6.08939394
GO:0042866	pyruvate biosynthetic process	-3.309736295	7.74075501
GO:0000184	nuclear-transcribed mRNA catabolic process, nonsense-mediated decay	-3.276408132	7.61174242
GO:0022613	ribonucleoprotein complex biogenesis	-3.27200407	3.57498666
GO:0006091	generation of precursor metabolites and energy	-3.265448577	3.56800426
M237	PID VEGFR1 2 PATHWAY	-3.247066312	10.5902503
M121	PID MTOR 4PATHWAY	-3.247066312	10.5902503
GO:0043161	proteasome-mediated ubiquitin-dependent protein catabolic process	-3.228822909	3.85947503
hsa04919	thyroid hormone signaling pathway	-3.22757809	7.42609017
GO:0046031	ADP metabolic process	-3.195769793	7.30727273
GO:1903578	regulation of ATP metabolic process	-3.195769793	7.30727273
GO:1903046	meiotic cell cycle process	-3.17919347	5.73869586
ko04520	Adherens junction	-3.177017984	10.1489899
GO:0046700	heterocycle catabolic process	-3.172902863	3.02369906
GO:0051640	organelle localization	-3.172902863	3.02369906
GO:0030968	endoplasmic reticulum unfolded protein response	-3.164535055	7.19219757
GO:1905477	positive regulation of protein localization to membrane	-3.164535055	7.19219757
GO:0019362	pyridine nucleotide metabolic process	-3.155562974	5.67922751
GO:0046496	nicotinamide nucleotide metabolic process	-3.155562974	5.67922751

GO:0010638	positive regulation of organelle organization	-3.148181416	3.19984076
GO:0033673	negative regulation of kinase activity	-3.138265595	4.75380196
M160	PID AVB3 INTEGRIN PATHWAY	-3.132084301	9.87469287
ko04918	Thyroid hormone synthesis	-3.132084301	9.87469287
GO:0031400	negative regulation of protein modification process	-3.131285294	3.18462758
GO:0051052	regulation of DNA metabolic process	-3.129239657	3.73667355
GO:0001933	negative regulation of protein phosphorylation	-3.129239657	3.73667355
GO:0070848	response to growth factor	-3.116092202	2.97850791
GO:0030947	regulation of vascular endothelial growth factor receptor signaling pathway	-3.109044959	16.607438
M277	PID INTEGRIN A4B1 PATHWAY	-3.109044959	16.607438
GO:0019439	aromatic compound catabolic process	-3.105890639	2.97043607
GO:0019080	viral gene expression	-3.09772789	5.53581267
GO:0006165	nucleoside diphosphate phosphorylation	-3.088839879	6.91976584
GO:0016052	carbohydrate catabolic process	-3.075075077	5.48045455
GO:0072524	pyridine-containing compound metabolic process	-3.075075077	5.48045455
GO:0046939	nucleotide phosphorylation	-3.074093954	6.86773753
GO:0009615	response to virus	-3.073297153	4.07090403
GO:0010035	response to inorganic substance	-3.07025106	3.36430604
GO:0006979	response to oxidative stress	-3.053827517	3.64553517
GO:0043687	post-translational protein modification	-3.050048839	4.03716725
hsa05230	Central carbon metabolism in cancer	-3.046113448	9.36829837
ko05205	Proteoglycans in cancer	-3.041594127	5.39946261
GO:0006397	mRNA processing	-3.034181281	3.32753767
GO:0051984	positive regulation of chromosome segregation	-3.033487368	15.6584416
M66	PID MYC ACTIV PATHWAY	-3.025382839	9.24971231
hsa04918	Thyroid hormone synthesis	-3.025382839	9.24971231
GO:0009152	purine ribonucleotide biosynthetic process	-3.018722205	4.53465506
GO:0009135	purine nucleoside diphosphate metabolic process	-3.002222195	6.61890646
GO:0009179	purine ribonucleoside diphosphate metabolic process	-3.002222195	6.61890646
GO:0031572	G2 DNA damage checkpoint	-2.997436999	15.2234848
GO:2001171	positive regulation of ATP biosynthetic process	-2.997436999	15.2234848
GO:0072594	establishment of protein localization to organelle	-2.986903541	3.27974539

hsa04520	Adherens junction	-2.984781677	9.02132435
GO:0009185	ribonucleoside diphosphate metabolic process	-2.960520342	6.47807866
GO:0006733	oxidoreduction coenzyme metabolic process	-2.955111307	5.19474364
GO:0043506	regulation of JUN kinase activity	-2.945279734	8.80394304
ko04810	Regulation of actin cytoskeleton	-2.944576222	5.17024014
GO:1901361	organic cyclic compound catabolic process	-2.923933133	2.8286217
GO:0010212	response to ionizing radiation	-2.91982855	6.34311869
ko04514	Cell adhesion molecules (CAMs)	-2.91982855	6.34311869
GO:0034655	nucleobase-containing compound catabolic process	-2.916058553	2.9947839
GO:0009260	ribonucleotide biosynthetic process	-2.905766152	4.3348228
GO:0034976	response to endoplasmic reticulum stress	-2.897333036	4.32017813
M14	PID AURORA B PATHWAY	-2.895448473	14.0524476
GO:2000785	regulation of autophagosome assembly	-2.895448473	14.0524476
GO:0071901	negative regulation of protein serine/threonine kinase activity	-2.893240815	6.25622665
GO:0019359	nicotinamide nucleotide biosynthetic process	-2.893240815	6.25622665
GO:0019363	pyridine nucleotide biosynthetic process	-2.893240815	6.25622665
GO:1902850	microtubule cytoskeleton organization involved in mitosis	-2.893240815	6.25622665
GO:0051129	negative regulation of cellular component organization	-2.890896001	2.80330156
hsa05205	Proteoglycans in cancer	-2.882589829	5.02793995
GO:1900542	regulation of purine nucleotide metabolic process	-2.880104479	6.21366729
GO:0000077	DNA damage checkpoint	-2.867071231	6.17168305
M176	PID FOXM1 PATHWAY	-2.863326639	13.7011364
GO:0072525	pyridine-containing compound biosynthetic process	-2.854139662	6.13026236
GO:0034620	cellular response to unfolded protein	-2.854139662	6.13026236
GO:0010498	proteasomal protein catabolic process	-2.847459589	3.40400903
M5493	WNT SIGNALING	-2.832853887	8.21041879
GO:0006164	purine nucleotide biosynthetic process	-2.831118606	4.20648923
GO:0006140	regulation of nucleotide metabolic process	-2.828576058	6.04906683
GO:0097190	apoptotic signaling pathway	-2.82840755	3.12276612
GO:0051701	interaction with host	-2.822618546	4.89326299
GO:0090305	nucleic acid phosphodiester bond hydrolysis	-2.814903861	4.17899584
GO:0031667	response to nutrient levels	-2.804717261	3.35538033

ko03010	Ribosome	-2.803402936	5.96999406
hsa04514	Cell adhesion molecules	-2.803402936	5.96999406
M174	PID UPA UPAR PATHWAY	-2.801613238	13.0487013
GO:0030260	entry into host cell	-2.79095957	5.93122786
GO:0044409	entry into host	-2.79095957	5.93122786
GO:0051806	entry into cell of other organism involved in symbiotic interaction	-2.79095957	5.93122786
GO:0051828	entry into other organism involved in symbiotic interaction	-2.778609993	5.89296188
GO:0051259	protein complex oligomerization	-2.774103533	4.78642318
GO:0046677	response to antibiotic	-2.767044456	4.09863054
GO:0009611	response to wounding	-2.766032477	2.86661912
GO:0007160	cell-matrix adhesion	-2.755055158	4.74498229
GO:0051591	response to cAMP	-2.74546045	7.77369439
GO:0072522	purine-containing compound biosynthetic process	-2.743545729	4.05959596
GO:0045936	negative regulation of phosphate metabolic process	-2.742421025	3.03963092
GO:0010563	negative regulation of phosphorus metabolic process	-2.737151881	3.0345817
GO:0071363	cellular response to growth factor stimulus	-2.737112797	2.84229137
GO:0006732	coenzyme metabolic process	-2.7349766	3.59964174
GO:0009132	nucleoside diphosphate metabolic process	-2.730125408	5.74471126
M12	PID RHOA PATHWAY	-2.71477673	12.1787879
hsa04810	Regulation of actin cytoskeleton	-2.708297271	4.644453
GO:0051304	chromosome separation	-2.695496057	7.53327085
GO:0000226	microtubule cytoskeleton organization	-2.695431087	2.9947839
GO:0030810	positive regulation of nucleotide biosynthetic process	-2.687221135	11.9140316
GO:1900373	positive regulation of purine nucleotide biosynthetic process	-2.687221135	11.9140316
hsa00500	Starch and sucrose metabolism	-2.687221135	11.9140316
hsa03010	Ribosome	-2.683047534	5.60373675
GO:0006754	ATP biosynthetic process	-2.683047534	5.60373675
GO:0051186	cofactor metabolic process	-2.679981317	2.98012754
GO:2001169	regulation of ATP biosynthetic process	-2.679227218	7.45640074
GO:1903580	positive regulation of ATP metabolic process	-2.66030929	11.6605416
M236	PID DELTA NP63 PATHWAY	-2.66030929	11.6605416
GO:0051100	negative regulation of binding	-2.660014269	5.53581267
GO:0009991	response to extracellular stimulus	-2.658497814	3.19249779

GO:0050804	modulation of chemical synaptic transmission	-2.656640412	3.49630274
GO:0006412	translation	-2.652414171	2.77172414
GO:0010639	negative regulation of organelle organization	-2.650245561	3.48795834
GO:0099177	regulation of trans-synaptic signaling	-2.650245561	3.48795834
GO:0044270	cellular nitrogen compound catabolic process	-2.647797188	2.76790634
M124	PID CXCR4 PATHWAY	-2.647244696	7.30727273
GO:0007044	cell-substrate junction assembly	-2.647244696	7.30727273
M288	PID HES HEY PATHWAY	-2.634013459	11.4176136
GO:0006469	negative regulation of protein kinase activity	-2.627100946	4.47384045
GO:0035967	cellular response to topologically incorrect protein	-2.626073341	5.43695887
GO:0043112	receptor metabolic process	-2.614918166	5.40478752
hsa04218	cellular senescence	-2.571064799	5.27982133
GO:2000045	regulation of G1/S transition of mitotic cell cycle	-2.560288788	5.24947753
GO:0009206	purine ribonucleoside triphosphate biosynthetic process	-2.560288788	5.24947753
M7955	SIG INSULIN RECEPTOR PATHWAY IN CARDIAC MYOCYTES	-2.558570132	10.7459893
GO:0005975	carbohydrate metabolic process	-2.550373581	2.85887039
GO:0009145	purine nucleoside triphosphate biosynthetic process	-2.549585962	5.21948052
GO:0007033	vacuole organization	-2.538955475	5.18982438
GO:0043043	peptide biosynthetic process	-2.535278711	2.67576565
GO:0044774	mitotic DNA integrity checkpoint	-2.526193846	6.76599327
GO:0051653	spindle localization	-2.510918776	10.3404803
GO:0006605	protein targeting	-2.508501722	3.30645825
GO:0007369	gastrulation	-2.507489796	5.10284408
GO:0009201	ribonucleoside triphosphate biosynthetic process	-2.497140483	5.07449495
GO:0071466	cellular response to xenobiotic stimulus	-2.497140483	5.07449495
GO:0072330	monocarboxylic acid biosynthetic process	-2.495586718	3.66410524
GO:0019058	viral life cycle	-2.495586718	3.66410524
GO:1902750	negative regulation of cell cycle G2/M phase transition	-2.483407511	6.58312858
GO:0043409	negative regulation of MAPK cascade	-2.476646043	5.01873127
GO:0031623	receptor internalization	-2.469438241	6.52435065
hsa04931	insulin resistance	-2.455610977	6.46661303

GO:0033044	regulation of chromosome organization	-2.455038668	3.6021767
GO:0006986	response to unfolded protein	-2.446403576	4.93734644
GO:1902808	positive regulation of cell cycle G1/S phase transition	-2.443012994	9.78652597
GO:0048675	axon extension	-2.441923176	6.40988836
GO:0043620	regulation of DNA-templated transcription in response to stress	-2.428372362	6.3541502
GO:0001704	formation of primary germ layer	-2.428372362	6.3541502
GO:0009168	purine ribonucleoside monophosphate biosynthetic process	-2.426566242	4.88454059
GO:0045981	positive regulation of nucleotide metabolic process	-2.421260769	9.61483254
GO:1900544	positive regulation of purine nucleotide metabolic process	-2.421260769	9.61483254
GO:0046434	organophosphate catabolic process	-2.419559431	4.05959596
GO:0009127	purine nucleoside monophosphate biosynthetic process	-2.416742758	4.85855899
GO:0051098	regulation of binding	-2.408840878	3.53252135
GO:0030855	epithelial cell differentiation	-2.407269626	2.57298335
GO:0007131	reciprocal meiotic recombination	-2.399923887	9.44905956
ko05223	Non-small cell lung cancer	-2.399923887	9.44905956
GO:0010574	regulation of vascular endothelial growth factor production	-2.399923887	9.44905956
hsa04930	Type II diabetes mellitus	-2.399923887	9.44905956
GO:0009896	positive regulation of catabolic process	-2.398628917	3.1701834
GO:0009142	nucleoside triphosphate biosynthetic process	-2.387644898	4.78224655
GO:0009166	nucleotide catabolic process	-2.387644898	4.78224655
GO:0010469	regulation of signaling receptor activity	-2.387644898	4.78224655
GO:0035825	homologous recombination	-2.378987875	9.28890601
M17	PID NOTCH PATHWAY	-2.378987875	9.28890601
GO:0016569	covalent chromatin modification	-2.37627366	3.142913
GO:2000134	negative regulation of G1/S transition of mitotic cell cycle	-2.375491717	6.14056532
GO:1900371	regulation of purine nucleotide biosynthetic process	-2.375491717	6.14056532
GO:0022618	ribonucleoprotein complex assembly	-2.373293497	3.97134387
GO:0031032	actomyosin structure organization	-2.368550249	4.73268959
GO:0030808	regulation of nucleotide biosynthetic process	-2.362590918	6.08939394
hsa05167	kaposi sarcoma-associated herpesvirus infection	-2.359092185	4.70829428

GO:1905475	regulation of protein localization to membrane	-2.359092185	4.70829428
GO:0070201	regulation of establishment of protein localization	-2.353713797	2.86934793
GO:0009410	response to xenobiotic stimulus	-2.350631958	3.92864125
GO:1902806	regulation of cell cycle G1/S phase transition	-2.349692801	4.68414918
GO:0044257	cellular protein catabolic process	-2.341934715	2.52132999
hsa05200	Pathways in cancer	-2.339123084	2.85440341
GO:0051205	protein insertion into membrane	-2.338264232	8.98435171
GO:0016049	cell growth	-2.332336458	3.08975591
GO:0072593	reactive oxygen species metabolic process	-2.328275595	3.8868472
GO:0007498	mesoderm development	-2.324620894	5.94087214
GO:0097193	intrinsic apoptotic signaling pathway	-2.320890152	3.87311275
GO:0010573	vascular endothelial growth factor production	-2.318451174	8.83944282
GO:1901016	regulation of potassium ion transmembrane transporter activity	-2.318451174	8.83944282
ko04510	Focal adhesion	-2.312670002	4.58999543
GO:1902807	negative regulation of cell cycle G1/S phase transition	-2.312201634	5.89296188
GO:0009156	ribonucleoside monophosphate biosynthetic process	-2.303555057	4.56704545
GO:1901292	nucleoside phosphate catabolic process	-2.303555057	4.56704545
M254	PID MYC REPRESS PATHWAY	-2.298988044	8.6991342
GO:2000378	negative regulation of reactive oxygen species metabolic process	-2.298988044	8.6991342
GO:0051784	negative regulation of nuclear division	-2.279863621	8.56321023
GO:0009165	nucleotide biosynthetic process	-2.270929273	3.33013731
GO:0071826	ribonucleoprotein complex subunit organization	-2.270101379	3.77962382
GO:0019693	ribose phosphate metabolic process	-2.253781334	2.76790634
GO:1901293	nucleoside phosphate biosynthetic process	-2.252930544	3.30432229
GO:0071456	cellular response to hypoxia	-2.250004576	4.43402471
GO:0035966	response to topologically incorrect protein	-2.250004576	4.43402471
GO:0070192	chromosome organization involved in meiotic cell cycle	-2.242588642	8.30371901
GO:0030073	insulin secretion	-2.241264364	4.4126043
GO:0009124	nucleoside monophosphate biosynthetic process	-2.23257559	4.39138986
GO:0071407	cellular response to organic cyclic compound	-2.231939161	2.97043607

GO:0016311	dephosphorylation	-2.231939161	2.97043607
ko00010	Glycolysis / Gluconeogenesis	-2.224418169	8.1797829
M295	SIG PIP3 SIGNALING IN CARDIAC MYOCTES	-2.224418169	8.1797829
GO:0036498	IRE1-mediated unfolded protein response	-2.224418169	8.1797829
GO:0000956	nuclear-transcribed mRNA catabolic process	-2.215350363	4.3495671
GO:0006323	DNA packaging	-2.206812928	4.32895304
GO:0043507	positive regulation of JUN kinase activity	-2.206546516	8.05949198
hsa05131	Shigellosis	-2.206546516	8.05949198
GO:0031670	cellular response to nutrient	-2.206546516	8.05949198
GO:0090307	mitotic spindle assembly	-2.206546516	8.05949198
GO:0006282	regulation of DNA repair	-2.205409551	5.49419002
GO:1901653	cellular response to peptide	-2.200028577	3.22922406
GO:0031331	positive regulation of cellular catabolic process	-2.188490136	3.2129968
GO:0036294	cellular response to decreased oxygen levels	-2.181495608	4.26826678
hsa04510	Focal adhesion	-2.173153276	4.24841438
GO:0007568	aging	-2.173060236	3.6055622
hsa05223	Non-small cell lung cancer	-2.171664564	7.82922078
GO:0046626	regulation of insulin receptor signaling pathway	-2.171664564	7.82922078
GO:0034599	cellular response to oxidative stress	-2.159665557	3.58199643
GO:0043627	response to estrogen	-2.154637665	7.71895006
ko04910	Insulin signaling pathway	-2.149663972	5.29512516
GO:1902115	regulation of organelle assembly	-2.140255775	4.17081777
GO:0046879	hormone secretion	-2.139783862	3.54721977
ko05412	Arrhythmogenic right ventricular cardiomyopathy (ARVC)	-2.13787634	7.61174242
GO:0072401	signal transduction involved in DNA integrity checkpoint	-2.121373144	7.50747198
GO:0072422	signal transduction involved in DNA damage checkpoint	-2.121373144	7.50747198
GO:1903779	regulation of cardiac conduction	-2.121373144	7.50747198
GO:0046718	viral entry into host cell	-2.117343277	5.18246293
GO:0046034	ATP metabolic process	-2.11365985	3.50188789
GO:2001020	regulation of response to DNA damage stimulus	-2.100163151	4.07771916
hsa05412	Arrhythmogenic right ventricular cardiomyopathy	-2.089112882	7.30727273
hsa01230	Biosynthesis of amino acids	-2.089112882	7.30727273

ko01230	Biosynthesis of amino acids	-2.089112882	7.30727273
GO:0033047	regulation of mitotic sister chromatid segregation	-2.089112882	7.30727273
GO:0071900	regulation of protein serine/threonine kinase activity	-2.088629901	2.8050951
GO:0034330	cell junction organization	-2.087965334	3.45770003
GO:0051235	maintenance of location	-2.0752757	3.43602166
GO:0072395	signal transduction involved in cell cycle checkpoint	-2.073342401	7.2111244
ko05100	Bacterial invasion of epithelial cells	-2.073342401	7.2111244
GO:0051656	establishment of organelle localization	-2.065049795	2.77843069
GO:0009914	hormone transport	-2.056434528	3.40400903
GO:0071453	cellular response to oxygen levels	-2.045863527	3.95415191
GO:0006511	ubiquitin-dependent protein catabolic process	-2.043283064	2.56096007
GO:0043407	negative regulation of MAP kinase activity	-2.042489182	7.02622378
GO:1900076	regulation of cellular response to insulin stimulus	-2.042489182	7.02622378
GO:0045860	positive regulation of protein kinase activity	-2.041784864	2.75226845
GO:0006110	regulation of glycolytic process	-2.027394556	6.93728423
GO:0071375	cellular response to peptide hormone stimulus	-2.025537241	3.35195997
GO:0061337	cardiac conduction	-2.025054058	4.87151515
GO:0019941	modification-dependent protein catabolic process	-2.018583365	2.53724747
GO:0034404	nucleobase-containing small molecule biosynthetic process	-2.015749095	3.8868472
GO:0032147	activation of protein kinase activity	-2.013351301	3.33158331
GO:0030433	ubiquitin-dependent ERAD pathway	-2.012513708	6.85056818
GO:0006446	regulation of translational initiation	-2.012513708	6.85056818
GO:0010817	regulation of hormone levels	-2.009732016	2.71645826
GO:0031669	cellular response to nutrient levels	-2.008320743	3.8703775

Supplementary Table 7. siRNA sequence

Pool Catalog Number	gene	siRNAs
L-013200-00	TBX20	GGAUCAACAUGGCCAUAUA
L-013200-00	TBX20	ACAACAAGAGGUACCGCUA
L-013200-00	TBX20	UCAAACAGAUGGUGUCUUU
L-013200-00	TBX20	UCACUGACAUUGAGAGGUA

Supplementary Table 8. H3K27me3 Datasets of IMR90 from ENCODE

Version	Database	Raw data
hg38	ENCODE	https://www.encodeproject.org/files/ENCFF825OWT/@@download/ENCFF825OWT.bam
hg38	ENCODE	https://www.encodeproject.org/files/ENCFF841PLU/@@download/ENCFF841PLU.bam
hg38	ENCODE	https://www.encodeproject.org/files/ENCFF146UYU/@@download/ENCFF146UYU.bam
hg38	ENCODE	https://www.encodeproject.org/files/ENCFF841PLU/@@download/ENCFF841PLU.bam
hg38	ENCODE	https://www.encodeproject.org/files/ENCFF648MBH/@@download/ENCFF648MBH.bam
hg38	ENCODE	https://www.encodeproject.org/files/ENCFF841PLU/@@download/ENCFF841PLU.bam
hg38	ENCODE	https://www.encodeproject.org/files/ENCFF308CPT/@@download/ENCFF308CPT.bam
hg38	ENCODE	https://www.encodeproject.org/files/ENCFF249ZNY/@@download/ENCFF249ZNY.bam
hg38	ENCODE	https://www.encodeproject.org/files/ENCFF501ZHJ/@@download/ENCFF501ZHJ.bam
hg38	ENCODE	https://www.encodeproject.org/files/ENCFF841PLU/@@download/ENCFF841PLU.bam
hg38	ENCODE	https://www.encodeproject.org/files/ENCFF969ZSU/@@download/ENCFF969ZSU.bam
hg38	ENCODE	https://www.encodeproject.org/files/ENCFF841PLU/@@download/ENCFF841PLU.bam
hg38	ENCODE	https://www.encodeproject.org/files/ENCFF538AFE/@@download/ENCFF538AFE.bam
hg38	ENCODE	https://www.encodeproject.org/files/ENCFF841PLU/@@download/ENCFF841PLU.bam
hg38	ENCODE	https://www.encodeproject.org/files/ENCFF202AKQ/@@download/ENCFF202AKQ.bam
hg38	ENCODE	https://www.encodeproject.org/files/ENCFF249ZNY/@@download/ENCFF249ZNY.bam
hg38	ENCODE	https://www.encodeproject.org/files/ENCFF397CDD/@@download/ENCFF397CDD.bam
hg38	ENCODE	https://www.encodeproject.org/files/ENCFF841PLU/@@download/ENCFF841PLU.bam
hg38	ENCODE	https://www.encodeproject.org/files/ENCFF689GXL/@@download/ENCFF689GXL.bam
hg38	ENCODE	https://www.encodeproject.org/files/ENCFF249ZNY/@@download/ENCFF249ZNY.bam
hg38	ENCODE	https://www.encodeproject.org/files/ENCFF191KVC/@@download/ENCFF191KVC.bam
hg38	ENCODE	https://www.encodeproject.org/files/ENCFF841PLU/@@download/ENCFF841PLU.bam
hg38	ENCODE	https://www.encodeproject.org/files/ENCFF179FAR/@@download/ENCFF179FAR.bam
hg38	ENCODE	https://www.encodeproject.org/files/ENCFF841PLU/@@download/ENCFF841PLU.bam
hg38	ENCODE	https://www.encodeproject.org/files/ENCFF548HLC/@@download/ENCFF548HLC.bam
hg38	ENCODE	https://www.encodeproject.org/files/ENCFF249ZNY/@@download/ENCFF249ZNY.bam
hg38	ENCODE	https://www.encodeproject.org/files/ENCFF226ZSG/@@download/ENCFF226ZSG.bam
hg38	ENCODE	https://www.encodeproject.org/files/ENCFF249ZNY/@@download/ENCFF249ZNY.bam
hg38	ENCODE	https://www.encodeproject.org/files/ENCFF520KCR/@@download/ENCFF520KCR.bam
hg38	ENCODE	https://www.encodeproject.org/files/ENCFF249ZNY/@@download/ENCFF249ZNY.bam
hg38	ENCODE	https://www.encodeproject.org/files/ENCFF836QKW/@@download/ENCFF836QKW.bam
hg38	ENCODE	https://www.encodeproject.org/files/ENCFF249ZNY/@@download/ENCFF249ZNY.bam
hg38	ENCODE	https://www.encodeproject.org/files/ENCFF905LXS/@@download/ENCFF905LXS.bam
hg38	ENCODE	https://www.encodeproject.org/files/ENCFF249ZNY/@@download/ENCFF249ZNY.bam
hg38	ENCODE	https://www.encodeproject.org/files/ENCFF398YEZ/@@download/ENCFF398YEZ.bam
hg38	ENCODE	https://www.encodeproject.org/files/ENCFF209UMN/@@download/ENCFF209UMN.bam
hg38	ENCODE	https://www.encodeproject.org/files/ENCFF356SHX/@@download/ENCFF356SHX.bam

hg38	ENCODE	https://www.encodeproject.org/files/ENCFF249ZNY/@@download/ENCFF249ZNY.bam
hg38	ENCODE	https://www.encodeproject.org/files/ENCFF391ENY/@@download/ENCFF391ENY.bam
hg38	ENCODE	https://www.encodeproject.org/files/ENCFF841PLU/@@download/ENCFF841PLU.bam
hg38	ENCODE	https://www.encodeproject.org/files/ENCFF444SKB/@@download/ENCFF444SKB.bam
hg38	ENCODE	https://www.encodeproject.org/files/ENCFF249ZNY/@@download/ENCFF249ZNY.bam
hg38	ENCODE	https://www.encodeproject.org/files/ENCFF866HDR/@@download/ENCFF866HDR.bam
hg38	ENCODE	https://www.encodeproject.org/files/ENCFF249ZNY/@@download/ENCFF249ZNY.bam
hg38	ENCODE	https://www.encodeproject.org/files/ENCFF269FHA/@@download/ENCFF269FHA.bam
hg38	ENCODE	https://www.encodeproject.org/files/ENCFF841PLU/@@download/ENCFF841PLU.bam
hg38	ENCODE	https://www.encodeproject.org/files/ENCFF870UBZ/@@download/ENCFF870UBZ.bam
hg38	ENCODE	https://www.encodeproject.org/files/ENCFF249ZNY/@@download/ENCFF249ZNY.bam
hg38	ENCODE	https://www.encodeproject.org/files/ENCFF993HLD/@@download/ENCFF993HLD.bam
hg38	ENCODE	https://www.encodeproject.org/files/ENCFF249ZNY/@@download/ENCFF249ZNY.bam
hg38	ENCODE	https://www.encodeproject.org/files/ENCFF904IRM/@@download/ENCFF904IRM.bam
hg38	ENCODE	https://www.encodeproject.org/files/ENCFF249ZNY/@@download/ENCFF249ZNY.bam
hg38	ENCODE	https://www.encodeproject.org/files/ENCFF694UJM/@@download/ENCFF694UJM.bam
hg38	ENCODE	https://www.encodeproject.org/files/ENCFF249ZNY/@@download/ENCFF249ZNY.bam
hg38	ENCODE	https://www.encodeproject.org/files/ENCFF215FBW/@@download/ENCFF215FBW.bam
hg38	ENCODE	https://www.encodeproject.org/files/ENCFF249ZNY/@@download/ENCFF249ZNY.bam
hg38	ENCODE	https://www.encodeproject.org/files/ENCFF155EKC/@@download/ENCFF155EKC.fastq.gz
hg38	ENCODE	https://www.encodeproject.org/files/ENCFF963CNY/@@download/ENCFF963CNY.fastq.gz

Supplementary Table 9. H3K27me3 ChIP-Seq of multiple organism

Mark	Data source	Cell	Organism	Link
H3K27me3	GSM1673980	pachytene spermatocytes	Macaca mulatta	https://sra-downloadb.be-md.ncbi.nlm.nih.gov/sos1/sra-pub-run-5/SRR1977652/SRR1977652.1
input	GSM1673981	pachytene spermatocytes	Macaca mulatta	https://sra-downloadb.be-md.ncbi.nlm.nih.gov/sos1/sra-pub-run-5/SRR1977653/SRR1977653.1
H3K27me3	GSM1673998	pachytene spermatocytes	Mus musculus	https://sra-downloadb.be-md.ncbi.nlm.nih.gov/sos1/sra-pub-run-5/SRR954037/SRR954037.2
input	GSM1674000	pachytene spermatocytes	Mus musculus	https://sra-downloadb.be-md.ncbi.nlm.nih.gov/sos1/sra-pub-run-5/SRR1049857/SRR1049857.2
H3K27me3	GSM1674024	pachytene spermatocytes	Bos taurus	https://sra-downloadb.be-md.ncbi.nlm.nih.gov/sos1/sra-pub-run-5/SRR1977481/SRR1977481.1
input	GSM1674026	pachytene spermatocytes	Bos taurus	https://sra-downloadb.be-md.ncbi.nlm.nih.gov/sos1/sra-pub-run-5/SRR1977482/SRR1977482.1
H3K27me3	GSM1674037	pachytene spermatocytes	Monodelphis domestica	https://sra-downloadb.be-md.ncbi.nlm.nih.gov/sos1/sra-pub-run-5/SRR1977641/SRR1977641.1
input	GSM1674039	pachytene spermatocytes	Monodelphis domestica	https://sra-downloadb.be-md.ncbi.nlm.nih.gov/sos1/sra-pub-run-5/SRR1977643/SRR1977643.1
H3K27me3	GSM1674050	pachytene spermatocytes	Gallus gallus	https://sra-downloadb.be-md.ncbi.nlm.nih.gov/sos1/sra-pub-run-5/SRR1977506/SRR1977506.1
input	GSM1674052	pachytene spermatocytes	Gallus gallus	https://sra-downloadb.be-md.ncbi.nlm.nih.gov/sos1/sra-pub-run-5/SRR1977507/SRR1977507.1
H3K27me3	GSM1673957	pachytene spermatocytes	Homo sapiens	https://sra-downloadb.be-md.ncbi.nlm.nih.gov/sos1/sra-pub-run-5/SRR1977543/SRR1977543.1
input	GSM1673958	pachytene spermatocytes	Homo sapiens	https://sra-downloadb.be-md.ncbi.nlm.nih.gov/sos1/sra-pub-run-5/SRR1977545/SRR1977545.1

Supplementary Table 10. Hi-C data of IMR-90, GM12878 and GM23248

Sample id	Cell	Database	Raw data
ENCFF718AWL	GM12878	ENCODE	https://www.encodeproject.org/files/ENCFF718AWL/@@download/ENCFF718AWL.hic
ENCFF856LGA	GM12878	ENCODE	https://www.encodeproject.org/files/ENCFF856LGA/@@download/ENCFF856LGA.bedpe.gz
ENCFF339ZMJOpen file information	GM12878	ENCODE	https://www.encodeproject.org/files/ENCFF339ZMJ/@@download/ENCFF339ZMJ.bedpe.gz
ENCFF768UBD	GM23248	ENCODE	https://www.encodeproject.org/files/ENCFF768UBD/@@download/ENCFF768UBD.hic
ENCFF350KKT	GM23248	ENCODE	https://www.encodeproject.org/files/ENCFF350KKT/@@download/ENCFF350KKT.bedpe.gz
ENCFF432KUX	GM23248	ENCODE	https://www.encodeproject.org/files/ENCFF432KUX/@@download/ENCFF432KUX.bedpe.gz
ENCFF999YXX	IMR-90	ENCODE	https://www.encodeproject.org/files/ENCFF999YXX/@@download/ENCFF999YXX.hic
ENCFF464KWY	IMR-90	ENCODE	https://www.encodeproject.org/files/ENCFF464KWY/@@download/ENCFF464KWY.bedpe.gz
ENCFF307RGV	IMR-90	ENCODE	https://www.encodeproject.org/files/ENCFF307RGV/@@download/ENCFF307RGV.bedpe.gz

Supplementary Figure 1. Functional enrichment for genes along the H3K4me1, H3K4me3 and H3K27ac breadth continuum. The color and size of each point represented the -$\log_{10}$ (p-value) values and enrichment scores.

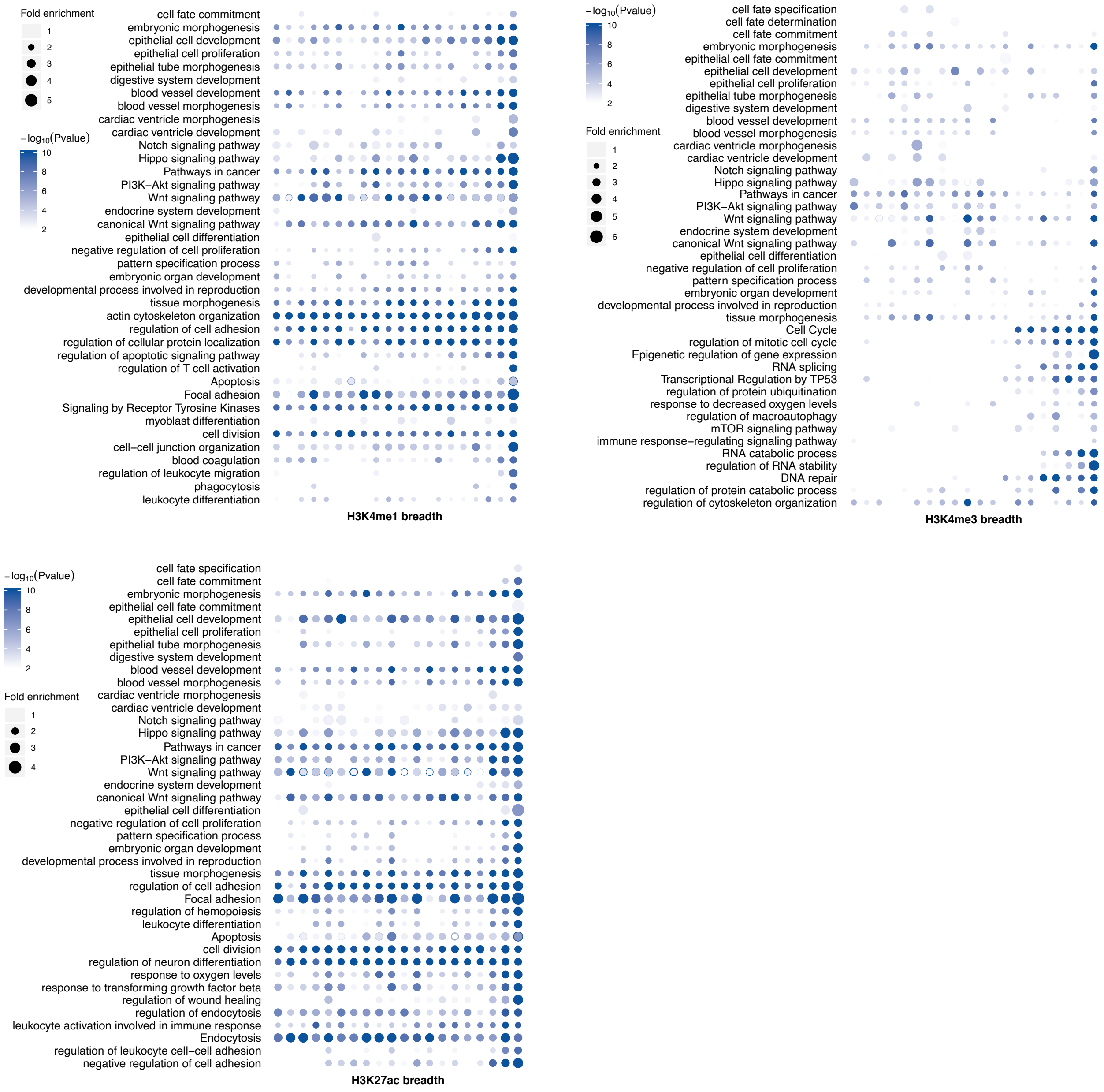

Supplementary Figure 2. The top 5% broadest H3K27me3 domains enrich for oncogene in NE2 cells. Enrichment expressed as -$\log_{10}$ (p-value) in Fisher's exact test. (dashed line) p = 0.05

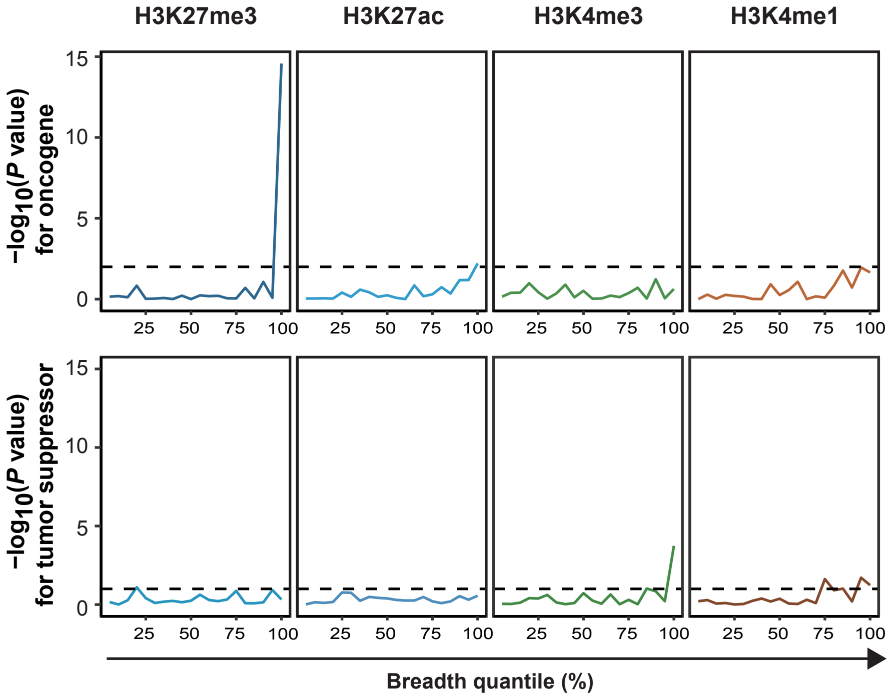

Supplementary Figure 3. H3K27me3 signal around transcription start sites. H3K27me3 ChIP signal sorted by breadth at -5 kb, +5 kb around transcription start sites (TSSs).

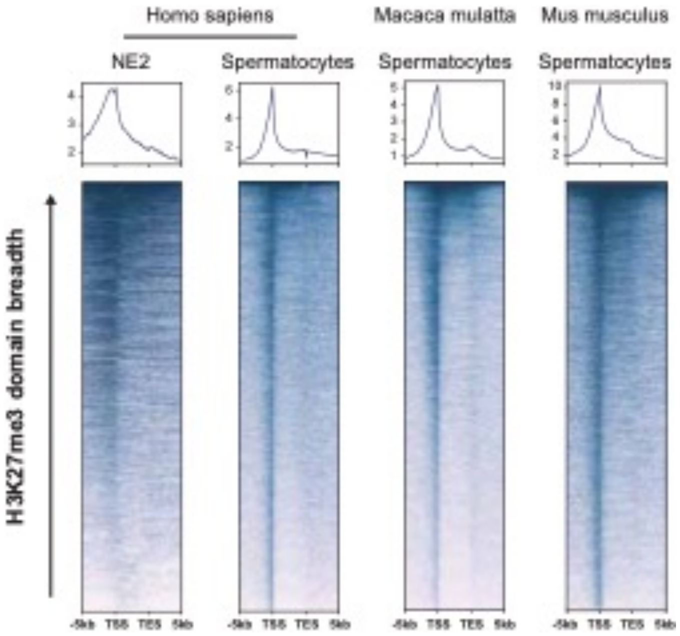

Supplementary Figure 4. Gene ontology fold enrichments and pvalue are correlated for GO terms shared between NE2 and IMR-90 GSDs related genes.

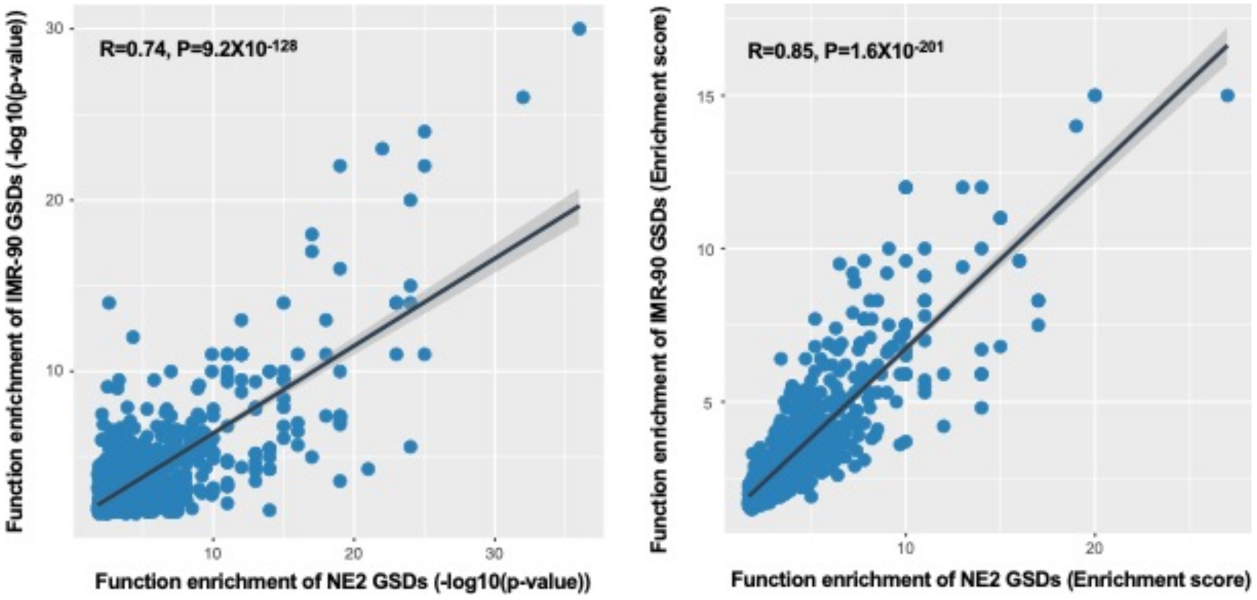

Supplementary Figure 5. Hi-C contact maps of GM23248 and GM12878. Position of peaks for grand H3K27me3 domain, narrow peaks and control peaks across TADs. TADs are normalized to position 0, representing the TAD center, and positions −1 and 1 represent the TAD boundary.

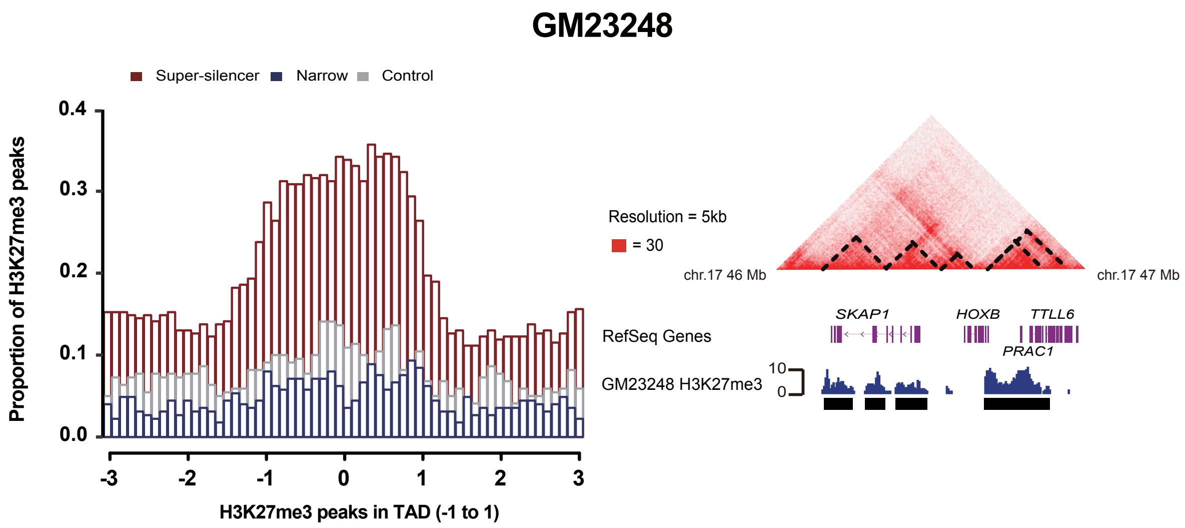

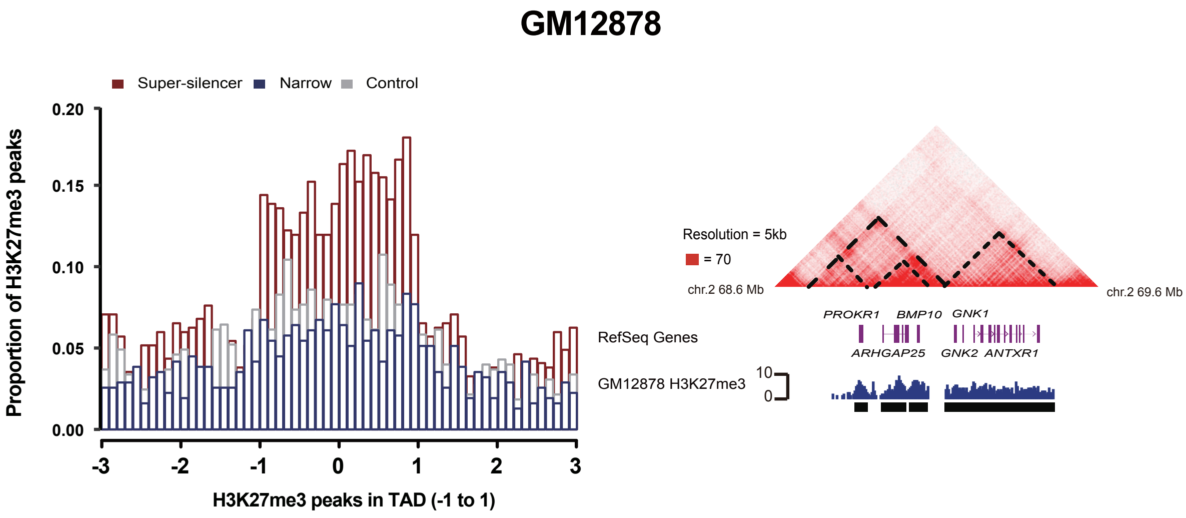

Supplementary Figure 6. Boxplot of different H3K27me3 signal between KYSE450 and NE2.

Wilcoxon rank sum test for *p*-value

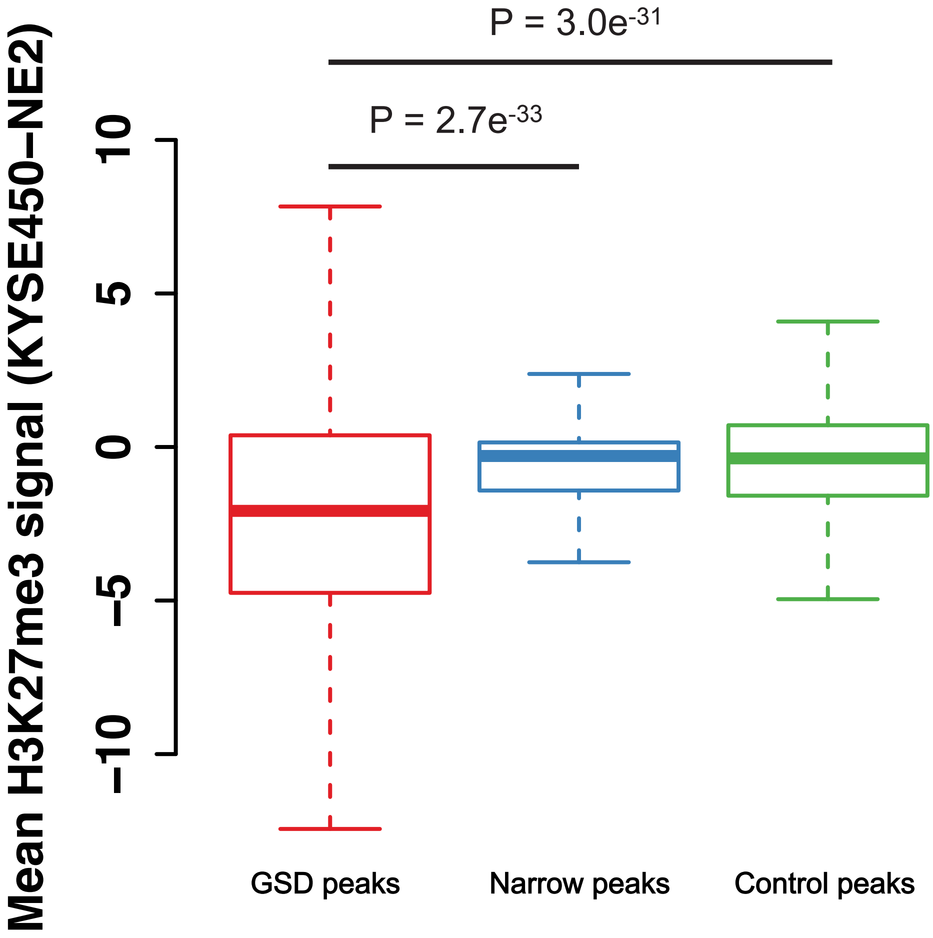

Supplementary Figure 7. Correlation between H3K27me3 difference and mRNA fold change for narrow domain and random domain marked gene. red, selected H3K27me3 losses and upregulated genes; blue, H3K27me3 gains and downregulated genes; black diagonal line, linear regression.

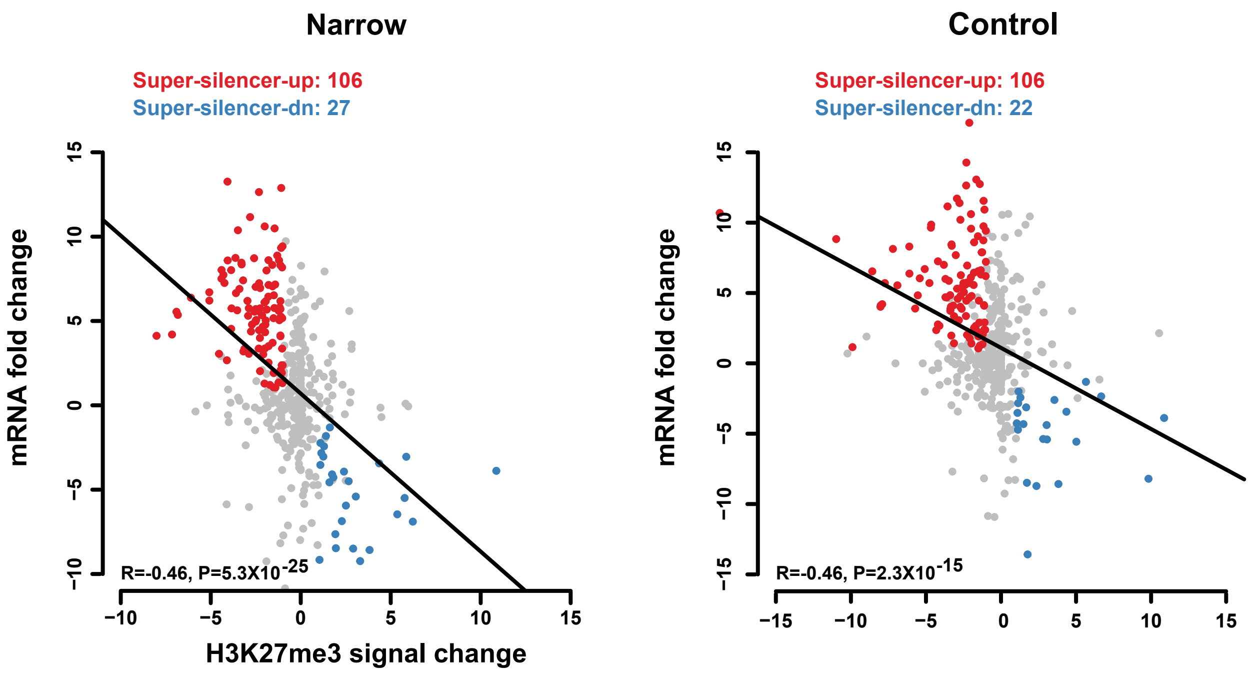

Supplementary Figure 8. Widespread loss of grand H3K27me3 silencer domains in KYSE510. In grand H3K27me3 domain marked gene, heatmaps showing H3K27me3 distribution in NE2 and KYSE510 cells. H3K27me3 on gene bodies (fifty equal-sized bins) ± 2 kb (ten equal-sized bins) is represented on the brown scale. Each row represents the same gene in NE2 and KYSE510 cells. H3K27me3 difference for GSDs-marked gene (mean values; x axis) plotted against expression of the corresponding genes (y axis; log2 values) in KYSE510 cells versus NE2 cells (along axes): red, selected H3K27me3 losses and upregulated genes; blue, H3K27me3 gains and downregulated genes; black diagonal line, linear regression.

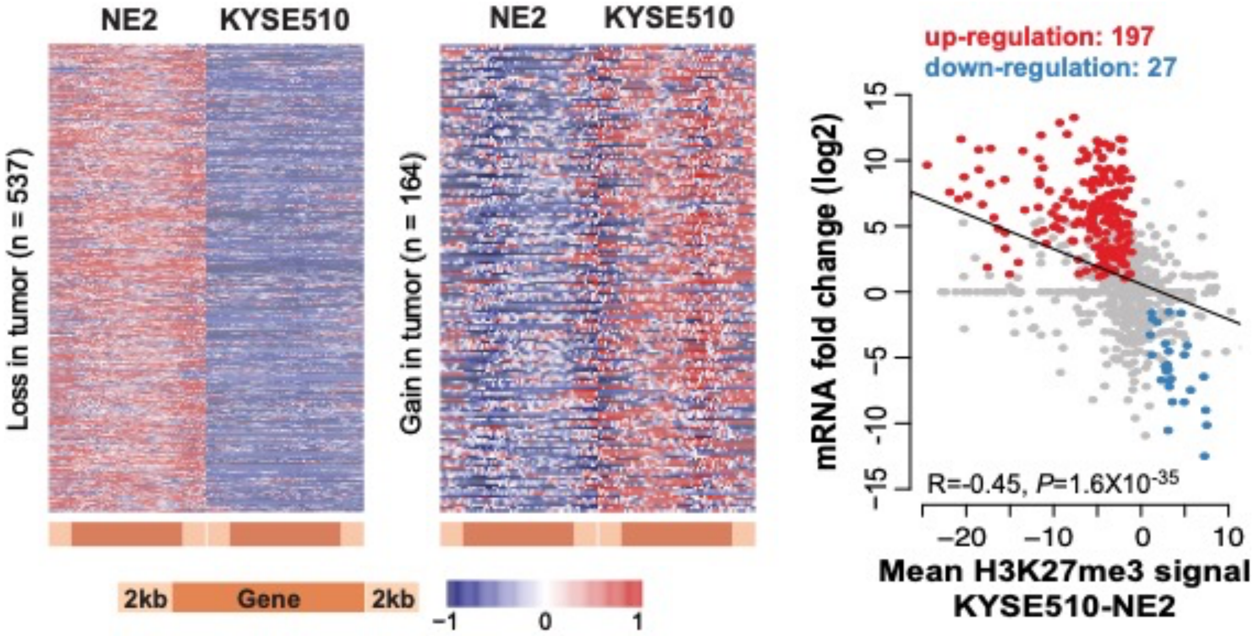

Supplementary Figure 9. Non-redundant enrichment clusters of differentially expressed GSDs associated genes. one per cluster, using a discrete color scale to represent statistical significance.

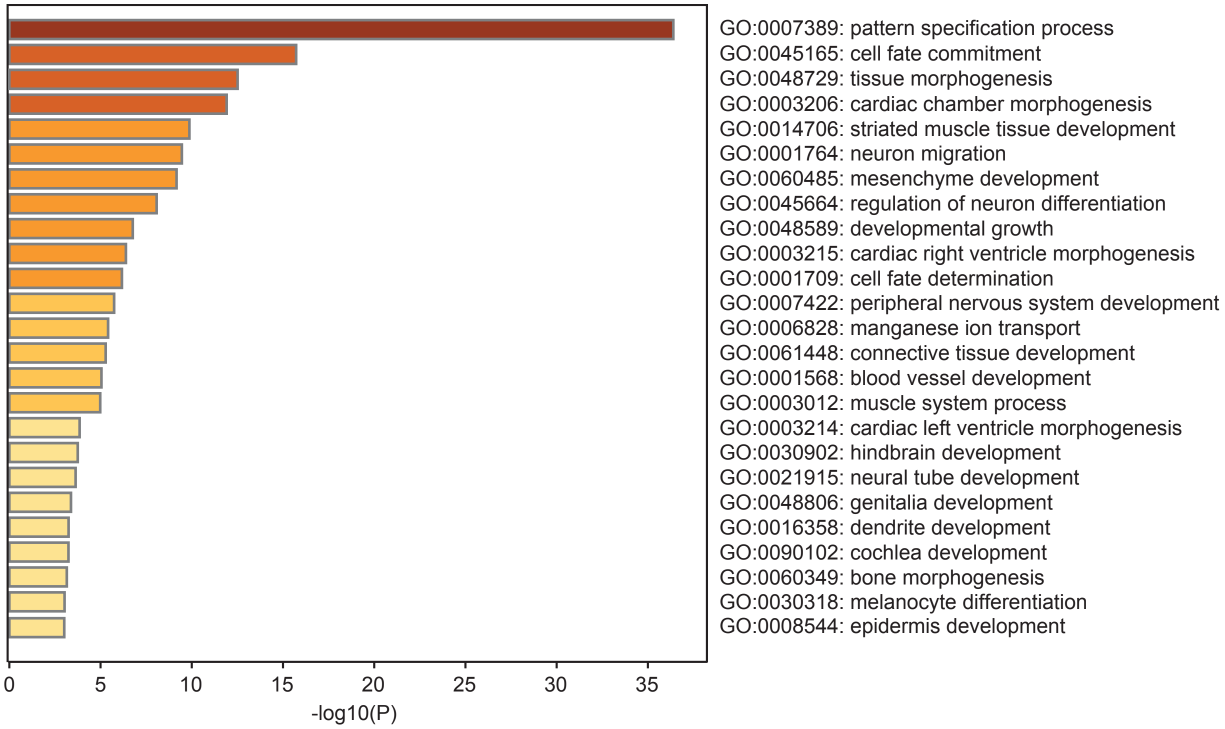

Supplementary Figure 10. GSEA of H3K27me3 losses in GSDs-marked genes between NE2 and KYSE510 normalized enrichment score (NES); false discovery rate-adjusted *P*-value (FDR).

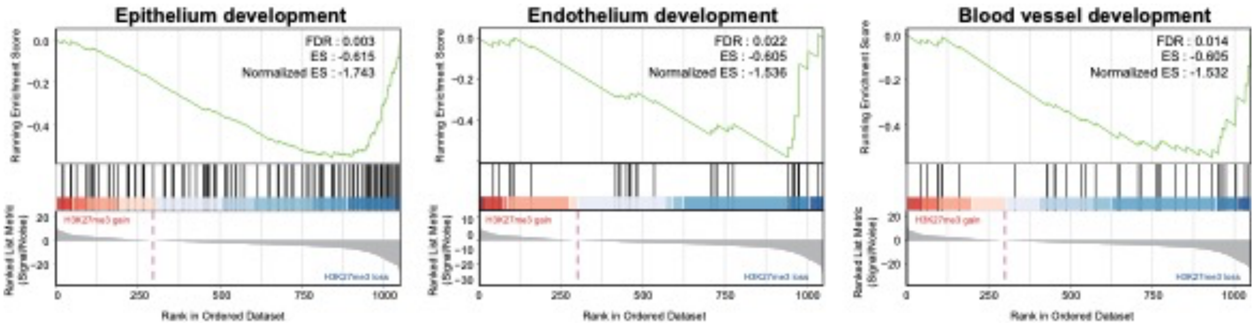

Supplementary Figure 11. Expression level of TBX20 in ESCC samples of TCGA. Wilcoxon rank sum test for *p*-value

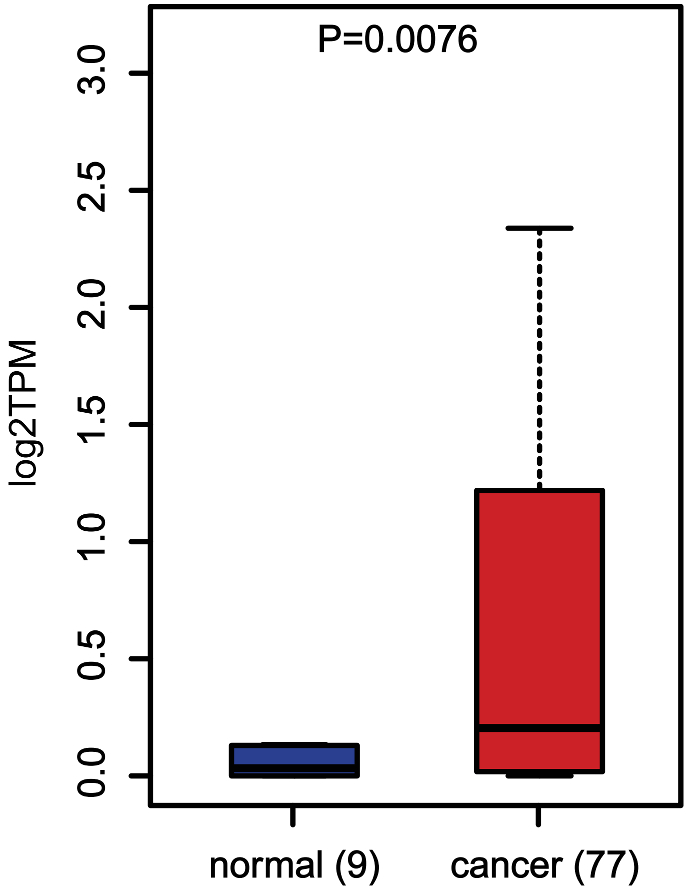

Supplementary Figure 12. Tumor weight of the solid tumors of ESCC animal model in TBX20 knockdown. *, ** or ***: significantly different from the corresponding control, $p < 0.05$, $p < 0.01$ or $p < 0.001$, respectively, by Student's t-test

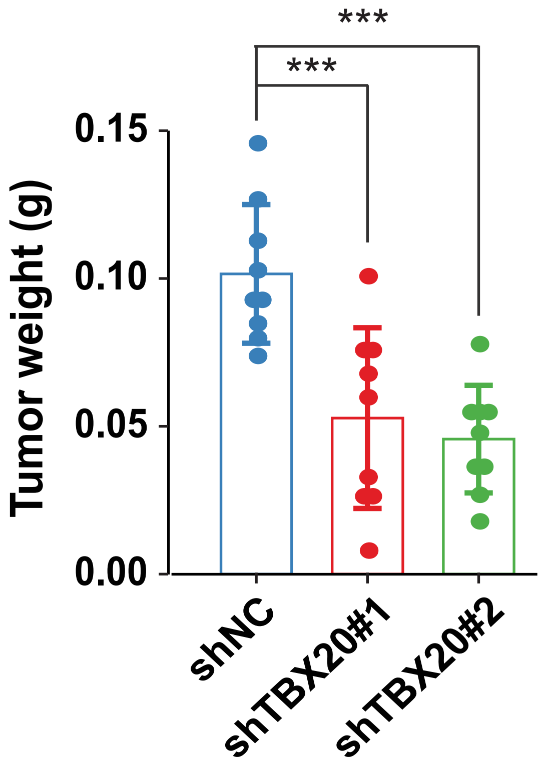

Supplementary Figure 13. Scatter plots depicting correlations in gene expression between replicates under all tested conditions. The expression of all genes was well correlated between biological replicates. “NC” represents cell lines transfected with a small RNA duplex as the negative control

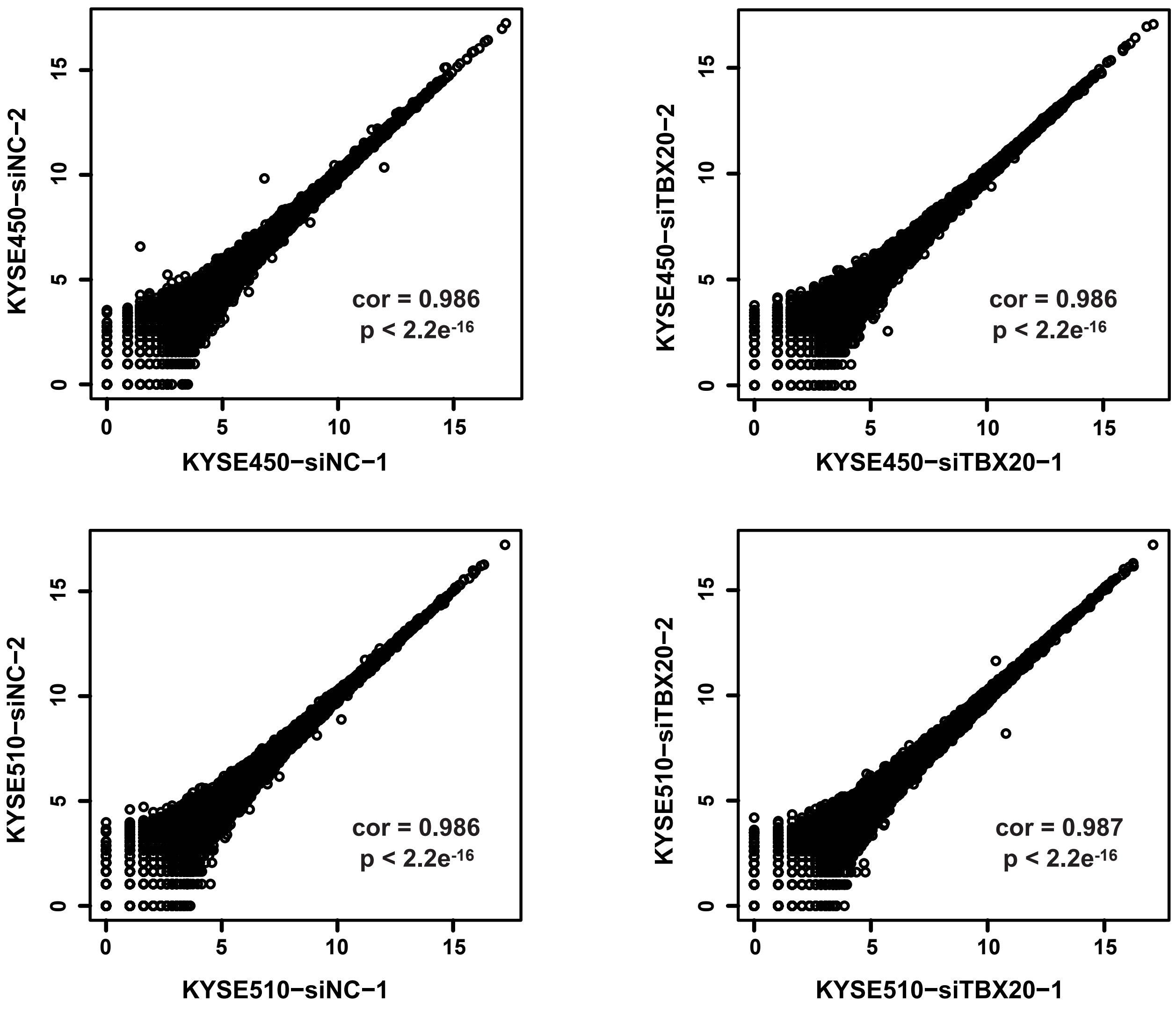

Supplementary Figure 14. Top TF binding motifs enriched in DEGs of TBX20-deletion KYSE510 cells. Motif and statistical analysis were performed in HOMER software.

Name	Motif	P-value
TBX20	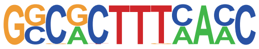	1e-10
POU4F3	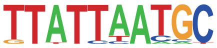	1e-10
CPHX	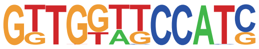	1e-9
HINFP	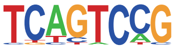	1e-8
SOX30	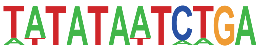	1e-7
MEIS1	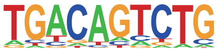	1e-6
GATA2	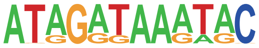	1e-6
POU6F1	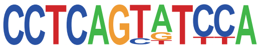	1e-5
SOX9	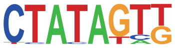	1e-4
SREBP-2	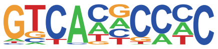	1e-4
SP2	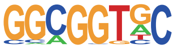	1e-3
