# Supplementary material for: Loss of grand histone H3 lysine 27 trimethylation domains mediated transcriptional activation in esophageal squamous cell carcinoma
Source: NPJ Genom Med. 2021 Aug 11;6:65. doi: 10.1038/s41525-021-00232-6 (PMC8358006; doi:10.1038/s41525-021-00232-6)
Supplement: Supplementary file 2 — Reporting Summary [file 41525_2021_232_MOESM2_ESM.pdf]

nature portfolio

Corresponding author(s): Jianzhong Su, NPJGENMEN-00962R

Last updated by author(s): Jun 9, 2021

Reporting Summary

Nature Portfolio wishes to improve the reproducibility of the work that we publish. This form provides structure for consistency and transparency in reporting. For further information on Nature Portfolio policies, see our Editorial Policies and the Editorial Policy Checklist.

Statistics

For all statistical analyses, confirm that the following items are present in the figure legend, table legend, main text, or Methods section.

n/a	Confirmed	
☐	☒	The exact sample size (*n*) for each experimental group/condition, given as a discrete number and unit of measurement
☐	☒	A statement on whether measurements were taken from distinct samples or whether the same sample was measured repeatedly
☐	☒	The statistical test(s) used AND whether they are one- or two-sided<br>*Only common tests should be described solely by name; describe more complex techniques in the Methods section.*
☐	☒	A description of all covariates tested
☐	☒	A description of any assumptions or corrections, such as tests of normality and adjustment for multiple comparisons
☐	☒	A full description of the statistical parameters including central tendency (e.g. means) or other basic estimates (e.g. regression coefficient) AND variation (e.g. standard deviation) or associated estimates of uncertainty (e.g. confidence intervals)
☐	☒	For null hypothesis testing, the test statistic (e.g. *F*, *t*, *r*) with confidence intervals, effect sizes, degrees of freedom and *P* value noted<br>*Give P values as exact values whenever suitable.*
☒	☐	For Bayesian analysis, information on the choice of priors and Markov chain Monte Carlo settings
☐	☒	For hierarchical and complex designs, identification of the appropriate level for tests and full reporting of outcomes
☒	☐	Estimates of effect sizes (e.g. Cohen's *d*, Pearson's *r*), indicating how they were calculated

Our web collection on statistics for biologists contains articles on many of the points above.

Software and code

Policy information about availability of computer code

Data collection	The accession numbers for raw data from public database or the source of processed dataset, were indicated in the Supplementary Data. Human reference genome sequence version hg38 was downloaded from the UCSC Genome Browser website.
Data analysis	the data and code used is publicly available at https://github.com/JiangQi1996/H3K27me3

For manuscripts utilizing custom algorithms or software that are central to the research but not yet described in published literature, software must be made available to editors and reviewers. We strongly encourage code deposition in a community repository (e.g. GitHub). See the Nature Portfolio guidelines for submitting code & software for further information.

Data

Policy information about availability of data

All manuscripts must include a data availability statement. This statement should provide the following information, where applicable:

- Accession codes, unique identifiers, or web links for publicly available datasets
- A description of any restrictions on data availability
- For clinical datasets or third party data, please ensure that the statement adheres to our policy

ChIP-seq and RNA-seq data are deposited in the Sequence Read Archive (SRA) database (PRJNA679744). The authors declare that all other data or information are all available from the author upon request.

Field-specific reporting

Please select the one below that is the best fit for your research. If you are not sure, read the appropriate sections before making your selection.

☒ Life sciences ☐ Behavioural & social sciences ☐ Ecological, evolutionary & environmental sciences

For a reference copy of the document with all sections, see nature.com/documents/nr-reporting-summary-flat.pdf

Life sciences study design

All studies must disclose on these points even when the disclosure is negative.

Sample size	For RNA-seq analysis, a total of 8 samples from 3 cell lines were examined. For ChIP-seq analysis, a total of 12 samples from 3 cell lines were examined.
Data exclusions	Not applicable
Replication	Two replications for RNA-Seq were provided. We confirmed that all attempts at replications were useful.
Randomization	Not applicable
Blinding	Not applicable

Reporting for specific materials, systems and methods

We require information from authors about some types of materials, experimental systems and methods used in many studies. Here, indicate whether each material, system or method listed is relevant to your study. If you are not sure if a list item applies to your research, read the appropriate section before selecting a response.

Materials & experimental systems

n/a	Involved in the study	
☐	☒	Antibodies
☐	☒	Eukaryotic cell lines
☒	☐	Palaeontology and archaeology
☐	☒	Animals and other organisms
☒	☐	Human research participants
☒	☐	Clinical data
☒	☐	Dual use research of concern

Methods

n/a	Involved in the study	
☐	☒	ChIP-seq
☒	☐	Flow cytometry
☒	☐	MRI-based neuroimaging

Antibodies

Antibodies used	2 ug of an anti-H3K27ac, anti-H3K4me3, anti-H3K27me3, anti-H3K4me1 antibody were used for each ChIP-seq experiment.
Validation	This antibody has been commercially confirmed and widely used in our and others' previous studies.

Eukaryotic cell lines

Policy information about cell lines

Cell line source(s)	The human ESCC cell lines KYSE450 and KYSE510 were provided by Dr. Yutaka Shimada (Kyoto University, Kyoto, Japan). The immorality normal esophageal epithelium NE2 cells were a gift from Dr. Enmin Li (Medical College of Shantou University, Guangdong, China). HEK293T cells were purchased from the ATCC.
Authentication	All of the cells were authenticated by short tandem repeat (STR) analysis and regularly tested for mycoplasma contamination.
Mycoplasma contamination	We confirmed that there is no mycoplasma contamination for used cells.
Commonly misidentified lines (See ICLAC register)	None

Animals and other organisms

Policy information about studies involving animals; ARRIVE guidelines recommended for reporting animal research

Laboratory animals	6-week-old female BALB/c nude mice
Wild animals	No wildtype animals were used in the present study.
Field-collected samples	No field collected samples were collected in the study.
Ethics oversight	Animal Care and Use Committee of the Chinese Academy of Medical Sciences Cancer Hospital (Beijing, China)

Note that full information on the approval of the study protocol must also be provided in the manuscript.

ChIP-seq

Data deposition

☒ Confirm that both raw and final processed data have been deposited in a public database such as GEO.

☒ Confirm that you have deposited or provided access to graph files (e.g. BED files) for the called peaks.

Data access links *May remain private before publication.*	ChIP-seq and RNA-seq data are deposited in the Sequence Read Archive (SRA) database (PRJNA679744). The authors declare that all other data are available from the author upon request. Free accession for review: https://dataview.ncbi.nlm.nih.gov/object/PRJNA679744?reviewer=b9v9apfa7sj5epvt6uvfsit9ds.
Files in database submission	KYSE450.H3K27ac_rep1.R1.fastq.gz, KYSE450.H3K27ac_rep1.R2.fastq.gz, KYSE450.H3K27me3_rep1.R1.fastq.gz, KYSE450.H3K27me3_rep1.R2.fastq.gz, KYSE450.H3K4me1_rep1.R1.fastq.gz, KYSE450.H3K4me1_rep1.R2.fastq.gz, KYSE450.H3K4me3_rep1.R1.fastq.gz, KYSE450.H3K4me3_rep1.R2.fastq.gz, KYSE450.Input_rep1.R1.fastq.gz, KYSE450.Input_rep1.R2.fastq.gz, KYSE450.RNA_rep1.R1.fastq.gz, KYSE450.RNA_rep1.R2.fastq.gz, KYSE450.RNA_rep2.R1.fastq.gz, KYSE450.RNA_rep2.R2.fastq.gz, KYSE510.H3K27ac_rep1.R1.fastq.gz, KYSE510.H3K27ac_rep1.R2.fastq.gz, KYSE510.H3K27me3_rep1.R1.fastq.gz, KYSE510.H3K27me3_rep1.R2.fastq.gz, KYSE510.H3K4me1_rep1.R1.fastq.gz, KYSE510.H3K4me1_rep1.R2.fastq.gz, KYSE510.H3K4me3_rep1.R1.fastq.gz, KYSE510.H3K4me3_rep1.R2.fastq.gz, KYSE510.Input_rep1.R1.fastq.gz, KYSE510.Input_rep1.R2.fastq.gz, KYSE510.RNA_rep1.R1.fastq.gz, KYSE510.RNA_rep1.R2.fastq.gz, KYSE510.RNA_rep2.R1.fastq.gz, KYSE510.RNA_rep2.R2.fastq.gz, KYSE510.siNC_rep1.R1.fastq.gz, KYSE510.siNC_rep1.R2.fastq.gz, KYSE510.siNC_rep2.R1.fastq.gz, KYSE510.siNC_rep2.R2.fastq.gz, KYSE510.siTBX20_rep1.R1.fastq.gz, KYSE510.siTBX20_rep1.R2.fastq.gz, KYSE510.siTBX20_rep2.R1.fastq.gz, KYSE510.siTBX20_rep2.R2.fastq.gz, NE2.H3K27ac_rep1.R1.fastq.gz, NE2.H3K27ac_rep1.R2.fastq.gz, NE2.H3K27me3_rep1.R1.fastq.gz, NE2.H3K27me3_rep1.R2.fastq.gz, NE2.H3K4me1-Rep.R1.fastq.gz, NE2.H3K4me1-Rep1.R2.fastq.gz, NE2.H3K4me3_Rep1.R1.fastq.gz, NE2.H3K4me3_Rep1.R2.fastq.gz, NE2.Input_rep1.R1.fastq.gz, NE2.Input_rep1.R2.fastq.gz, NE2.RNA_rep1.R1.fastq.gz, NE2.RNA_rep1.R2.fastq.gz, NE2.RNA_rep2.R1.fastq.gz, NE2.RNA_rep2.R2.fastq.gz
Genome browser session (e.g. UCSC)	Not applicable

Methodology

Replicates	Two replications for RNA-Seq were provided.
Sequencing depth	Each library was sequenced to an average of 30 million raw reads on HiSeq X-Ten sequencing platform. Sequencing was performed using the 150 bp pair-end read platform.
Antibodies	Anti-H3K27ac, anti-H3K4me3, anti-H3K27me3, anti-H3K4me1 antibody were used for each ChIP-seq experiment.
Peak calling parameters	Significant H3K27me3 peaks were called by using SICER2 with default parameter values, while H3K27ac, H3K4me3 and H3K4me1 were analyzed with all default parameters except -w 200 and –g 400. H3K27me3 peaks were merged within a distance of 2kb.
Data quality	Quality control for the alignment BAM files was performed with SAMtools, enabling only uniquely mapped reads to be retained, and PCR duplicates were removed by Picard ("Picard Toolkit" 2019) for subsequent analyses.
Software	ChIP-Seq raw reads were aligned to the human reference genome sequence version hg38 using Bowtie2 (version 2.3.4). Significant H3K27me3 peaks were called by using SICER2 with default parameter values, while H3K27ac, H3K4me3 and H3K4me1 were analyzed with all default parameters except -w 200 and –g 400. Identified peaks were annotated by intersection with the reference using BEDTools v2.25.0. We then used the deepTools bamCompare function to calculate ChIP-Seq signal, subtract corresponding background input signal, normalize the number of reads per bin in RPKM method and generate BigWig format files. The input-subtracted peak signal within a region was measured as a RPKM value using bigWigAverageOverBed.
